# Chalasoergodimers A–E, heterodimers with multiple polymerization modes from a marine-derived *Chaetomium* sp. fungus

**DOI:** 10.1007/s13659-025-00544-5

**Published:** 2025-09-11

**Authors:** Ze-Hong Lin, Han-Wen Shan, Li-Kun Yang, Tian-Tian Sun, Li-Ying He, Hui-Fang Du, Ya-Hui Zhang, Shan Liu, Xu Wang, Du-Qiang Luo, Fei Cao

**Affiliations:** 1 College of Pharmaceutical Sciences, Key Laboratory of Medicinal Chemistry and Molecular Diagnostics of Education Ministry of China, State Key Laboratory of New Pharmaceutical Preparations and Excipients, Hebei University, Baoding, 071002 People’s Republic of China; 2 Zhejiang Fonow Medicine Co., Ltd, Dongyang, 322106 People’s Republic of China; 3 College of Life Sciences, Hebei University, Baoding, 071002 People’s Republic of China

**Keywords:** Marine-derived fungus, *Chaetomium* sp., Chaetoglobosin, Cytotoxic activity

## Abstract

Five new heterodimers, chalasoergodimers A–E (**1**–**5**), and three known heterodimers (**6**–**8**), along with four chaetoglobosin monomers (**9**–**12**), were isolated from a marine-derived *Chaetomium* sp. fungus. The structures of new compounds **1**–**5** were elucidated by HRESIMS, NMR, chemical calculated ^13^C NMR and ECD methods. Among them, compound **1** was derived from C-2′ substitution of chaetoglobosin Fex (**9**) with ergosta-4,6,8(14),22-tetraen-3*β*-ol, representing a new dimerization mode among chaetoglobosin-ergosterol derivative hybrids. Compound **2** featured substitution at NH-1′ and constituted the first example of this dimeric type bearing an *R*-configuration at C-3′′. Compounds **3**–**5** were formed via a Diels–Alder cycloaddition between chaetoglobosins and 14-dehydroergosterol. Furthermore, it was revealed that compound **9**–**12** exhibited the significant cytotoxic activity against the human non-small cell lung cancer cell (A549), with compound **12** showing the most potent effect at an IC_50_ of 5.14 μM.

**Graphical Abstract:**

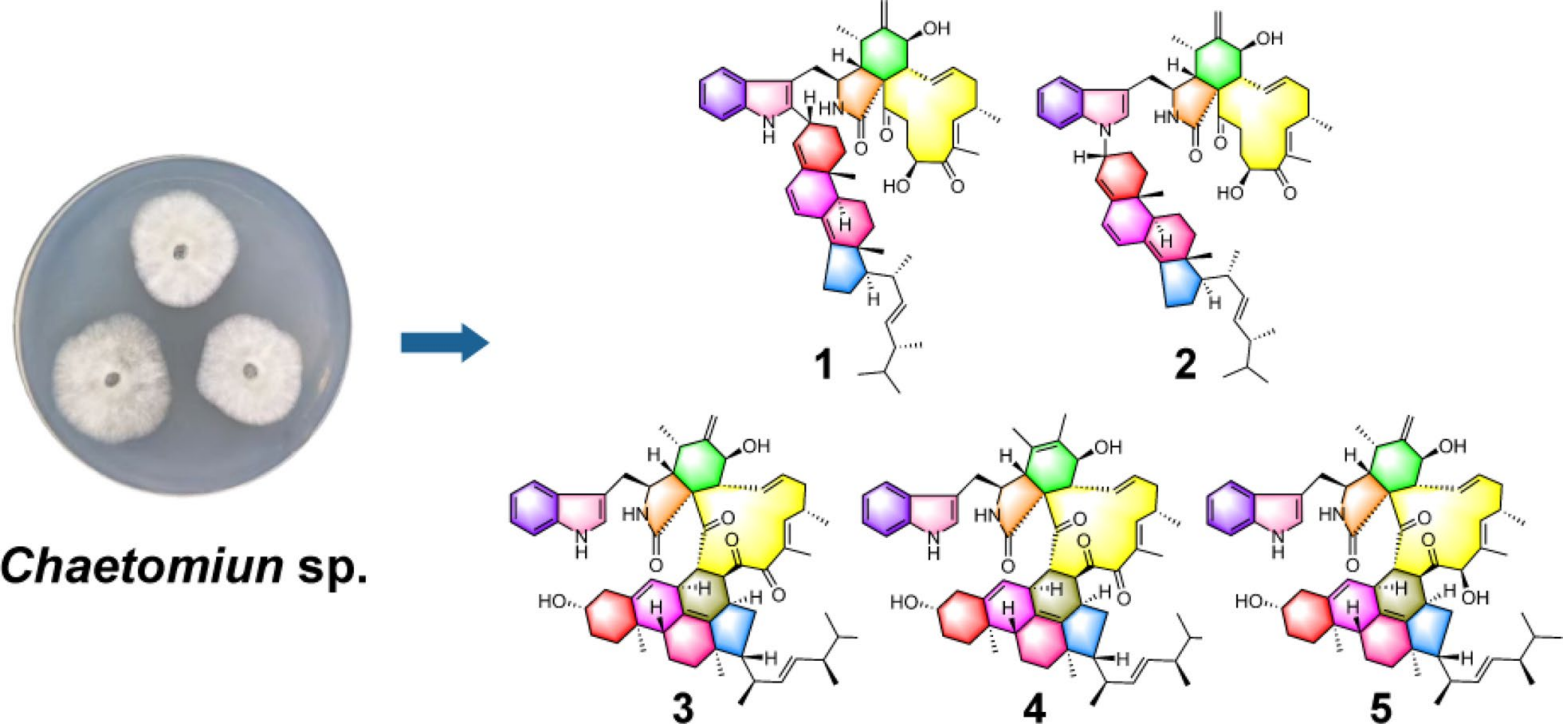

**Supplementary Information:**

The online version contains supplementary material available at 10.1007/s13659-025-00544-5.

## Introduction

Complex biosynthetic mechanisms underlay the structural diversity and complexity of natural products (NPs) [1]. Extensive exploration of NPs resources contributed to the identification of candidates with promising pharmacological activity, facilitating the discovery of lead compounds with high target specificity and potent biological efficacy [2–4]. The discovery of NPs with new scaffolds and exceptional bioactivities continued to captivate chemists and biologists [5]. In organisms, enzymatic reactions constituted the foundation of NPs biosynthesis: primary metabolites underwent enzymatic transformations into intermediates, which were further processed into final products by multi-enzyme complexes and tailoring enzymes [6, 7]. Additionally, non-enzymatic pathways, which was driven by chemical reactivity, evolutionary pressure, and the acidic or basic nature of the medium, functioned independently of enzymes and complemented enzymatic routes in contributing to NP biosynthesis [8].

Guided by their structural chemical properties, natural products underwent dimerization of secondary metabolites into homodimers or heterodimers through enzymatic and non-enzymatic polymerization reactions [9]. Reported dimeric NPs included coumarins [10], terpenoids [11], flavonoids [12], alkaloids [13], and others. Enzymatic dimerization typically involved cyclases and condensing enzymes [14]. Cyclases catalyzed cyclization reactions between monomers to form dimers. For instance, the FAD-dependent enzyme MaDA was identified as a monofunctional enzyme that catalyzed Diels–Alder reactions [15]. Condensing enzymes catalyze monomer condensation to form dimers. For example, AsuC3 and AsuC4 catalyzed condensation reactions to form polyketide side chains, which were critical biosynthetic steps for dimer formation [16]. In addition, certain oxidases catalyzed radical generation to participate in dimer formation [17]. Non-enzymatic reactions, guided by chemical properties, also occurred through spontaneous cycloaddition or condensation processes under physiological conditions [18].

Polymerization of two monomers altered the structural framework, enabling certain dimers to exhibit enhanced or even novel bioactivities compared to monomers [19]. Dibohemamine B, a methylene‑bridged dimer of two bohemamine monomers, demonstrated potent anti-non-small cell (A549) activity, whereas the bohemamine monomer was inactive [20]. Similarly, the terpene-nonadride heterodimer bipoterpride B showed notably enhanced biological activity compared to its monomers [21]. Enhanced bioactivity may arise from structural and conformational changes following dimerization that improved target binding [22]. Alternatively, modifications in chemical properties, such as increased structural stability and altered polarity, boosted bioavailability and efficacy in vivo [23, 24]. Overall, the identification of dimers with novel skeletons significantly advanced our comprehension of the polymerization mechanisms of natural product monomers and facilitated the screening of lead compounds exhibiting promising bioactivities.

**Fig. 1:**
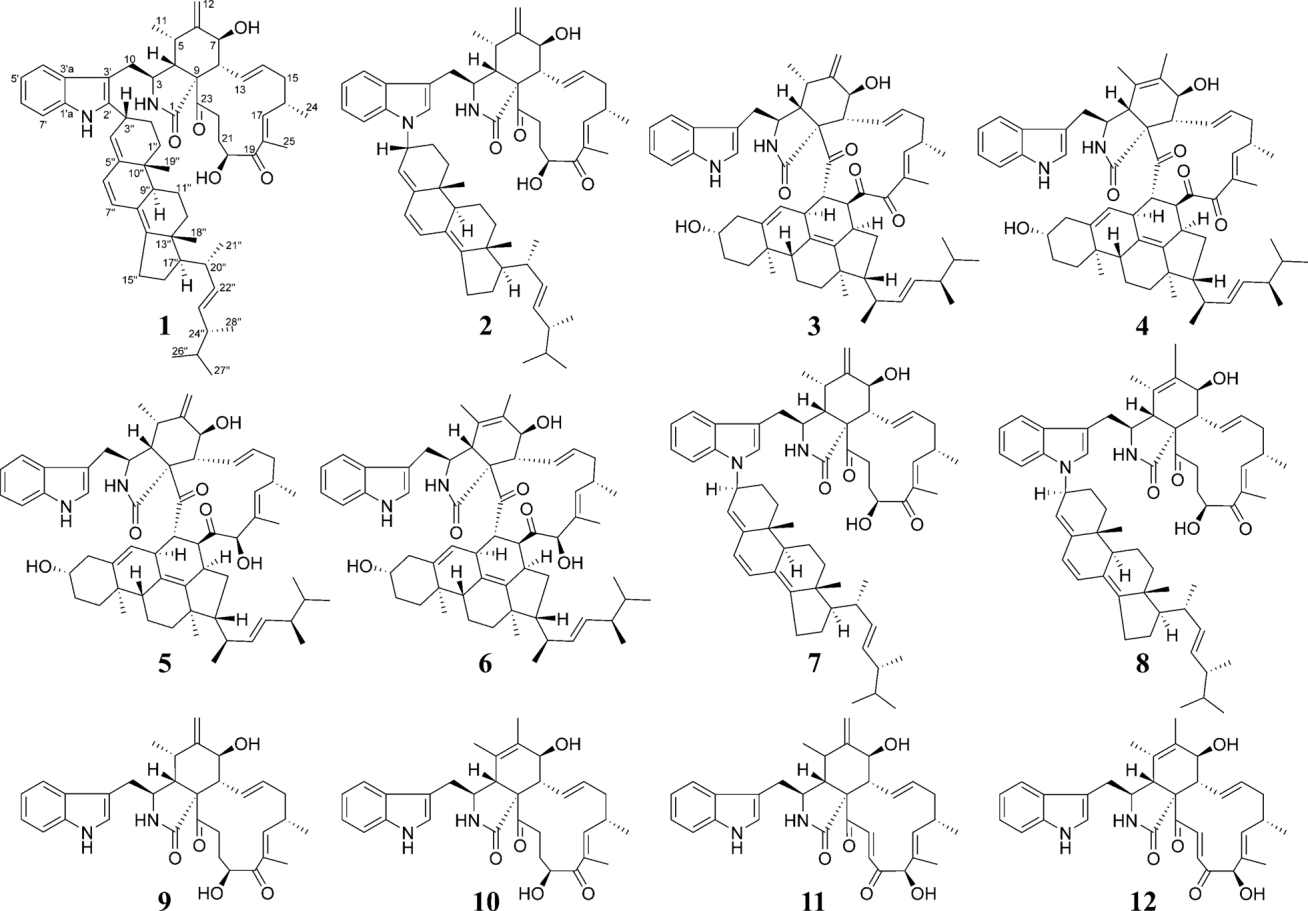
Chemical structures of compounds **1**–**12**

The fungi of *Chaetomium* species were reported to be capable of producing a variety of heterodimeric compounds [25, 26]. In this work, a marine-derived fungus *Chaetomium* sp. was chosen as the research subject. As a result, five new heterodimeric compounds (**1**–**5**), three known heterodimers, including ergochaeglobosin A (**6**) [27], ergochaeglobosin E (**7**) [27], ergochaeglobosin D (**8**) [27], and four known chaetoglobosin monomers, including chaetoglobosin Fex (**9**) [28], chaetoglobosin E (**10**) [29], chaetoglobosin D (**11**) [29], and chaetoglobosin B (**12**) [29], were obtained from this fungal strain (Fig. 1). Notably, the polymerization pattern of chaetoglobosin and ergosterol derivative in **1** marked the first report of this specific dimer type. Subsequently, the cytotoxicities of these compounds were also assessed.

## Results and discussion

**Fig. 2:**
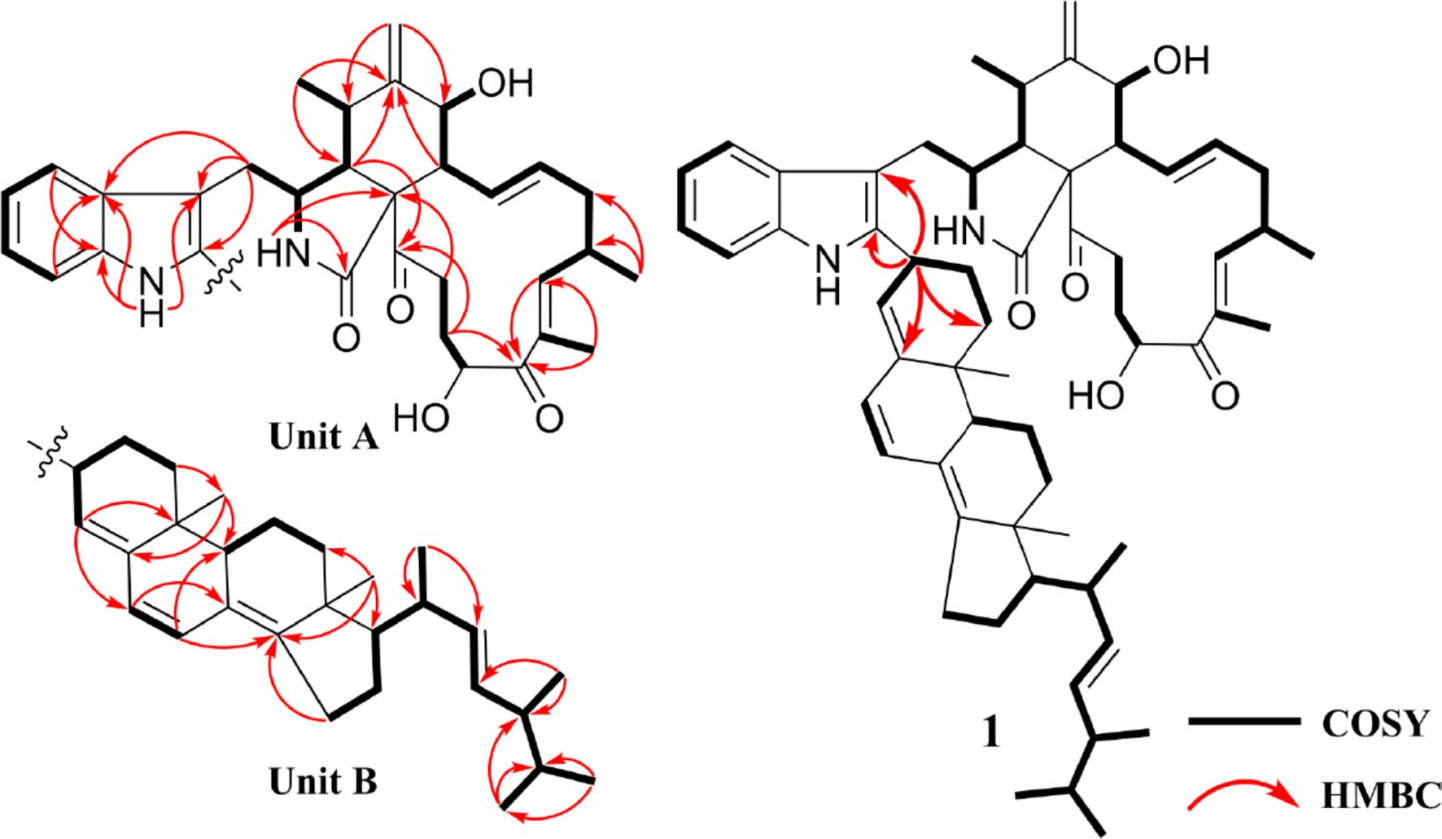
The ^1^H-^1^H COSY and key HMBC correlations of **1**

Chalasoergodimer A (**1**) was isolated as a brown powder. Its molecular formula was determined to be C_60_H_78_N_2_O_5_, indicating 23 degrees of unsaturation, based on the HRESIMS ion peak at *m*/*z* 929.5818 [M + Na]⁺ (calcd for C₆₀H₇₈N₂O₅Na^+^, 929.5808). The ^1^H NMR and HSQC spectra (Table 1 and Fig. S7) revealed the presence of an indolyl moiety, characterized by four coupled aromatic protons at *δ*_H_ 7.40 (d, 1H, *J* = 7.8 Hz), 7.28 (d, 1H, *J* = 7.8 Hz), 7.14 (dd, 1H, *J* = 7.8, 7.8 Hz), and 7.11 (dd, 1H, *J* = 7.8, 7.8 Hz). Eight non-terminal olefinic protons were observed at *δ*_H_ 6.31 (dd, 1H, *J* = 13.8, 9.6 Hz), 6.26 (d, 1H, *J* = 9.6 Hz), 6.19 (d, 1H, *J* = 9.6 Hz), 5.98 (d, 1H, *J* = 9.6 Hz), 5.51 (d, 1H, *J* = 4.8 Hz), 5.39 (ddd, 1H, *J* = 14.4, 10.8, 2.4 Hz), and two signals at *δ*_H_ 5.24 (dd, 1H, *J* = 15.6, 7.2 Hz). Two terminal olefinic protons appeared at *δ*_H_ 5.32 (s, 1H) and 5.13 (s, 1H). Nine methyl groups were detected at *δ*_H_ 1.87 (s, 3H), 1.10 (d, 3H, *J* = 6.6 Hz), 1.06 (d, 3H, *J* = 6.6 Hz), 1.06 (d, 3H, *J* = 6.6 Hz), 0.96 (s, 3H), 0.95 (s, 3H), 0.94 (d, 3H, *J* = 7.8 Hz), 0.85 (d, 3H, *J* = 6.6 Hz), and 0.84 (d, 3H, *J* = 6.6 Hz). The ^13^C NMR (Table 1) and HSQC spectra displayed 60 carbon signals, including two ketone carbonyls (*δ*_C_ 208.0 and 203.7), one amide carbonyl (*δ*_C_ 173.6), 22 olefinic carbons, two oxygenated carbons (*δ*_C_ 71.5 and 70.2), and 33 aliphatic carbons. The planar structure of **1** was further confirmed by analysis of ^1^H-^1^H COSY and HMBC spectra (Fig. 2). The ^1^H-^1^H COSY correlations of H-4′/H-5′/H-6′/H-7′, together with HMBC correlations from NH-1′ to C-1′a, C-3′, C-3′a, from H-4′ to C-1′a, and from H-7′ to C-3′a, suggested that the indole moiety contained only five hydrogen atoms. The ^1^H-^1^H COSY correlations of H-2/H-3/H-4/H-5/H-11, H-7/H-8/H-13/H-14/H-15/H-16/H-17, and H-20/H-21/H-22, along with the HMBC correlations from NH-2 to C-1, C-9, from H-4 to C-6, C-23, from H-12 to C-5, C-7, from H-17 to C-19, C-25, and from H-21 to C-19, C-23, further established **unit A** as the C-2′ dehydrogenated derivative of chaetoglobosin Fex (**9**). The characteristic structure included amide group at C-1, carbonyl groups at C-19 and C-23, a terminal alkene at C-12, and a hydroxyl group at C-20. In the structure of **unit B**, ^1^H-^1^H COSY correlations of H-1′′/H-2′′/H-3′′/H-4′′, H-6′′/H-7′′, H-9′′/H-11′′/H-12′′, H-15′′/H-16′′/H-17′′/H-20′′/H-22′′/H-23′′/H-24′′/H-25′′/H-26′′, together with HMBC correlations from H-3′′ to C-5′′, from H-4′′ to C-6′′, C-10′′, from H-6′′ to C-8′′, from H-7′′ to C-9′′, C-14′′, and from H-18′′ to C-12′′, C-14′′, C-17′′, indicated that **unit B** possessed a ergosta-4,6,8(14),22-tetraen-3*β*-ol skeleton [30] substituted at C-3′′. Moreover, the key HMBC correlations from H-3′′ to C-2′, C-3′, along with the absence of a proton signal at C-2′ in the HSQC spectrum, suggested that the planar structure of **1** consisted of chaetoglobosin Fex and ergosta-4,6,8(14),22-tetraen-3*β*-ol moiety, connected through a C–C single bond between C-2′ and C-3′′, with the hydroxyl group at C-3′′ eliminated.

**Table 1:** ^1^H (600 MHz) and ^13^C (150 MHz) NMR data of compounds **1** and **2** in CDCl_3_

No	**1**		**2**		No	**1**		**2**	
	*δ*_H_ (*J* in Hz)	*δ*_C_	*δ*_H_ (*J* in Hz)	*δ*_C_		*δ*_H_ (*J* in Hz)	*δ*_C_	*δ*_H_ (*J* in Hz)	*δ*_C_
1		173.6		173.8	5′	7.11, dd (7.8, 7.8)	120.1	7.13, dd (7.8, 7.8)	119.6
2	5.53, brs		5.64, brs		6′	7.14, dd (7.8, 7.8)	121.7	7.20, dd (7.8, 7.8)	121.9
3	3.50, m	52.5	3.42, m	52.7	7′	7.28, d (7.8)	111.1	7.40, d (7.8)	110.3
4	2.71, m	48.2	2.67, m	47.9	1′′a	1.67, m	31.7	1.87, m	34.4
5	2.85, m	32.2	2.85, m	32.1	1′′b	1.40, m		1.64, m	
6		148.1		148.2	2′′a	2.24, m	26.3	2.18, m	27.1
7	3.94, d (10.8)	70.2	3.93, d (10.2)	70.0	2′′b	1.77, m		2.01, m	
8	2.66, m	49.3	2.66, m	49.5	3′′	3.77, m	32.2	5.07, m	54.2
9		62.8		62.9	4′′	5.51, d (4.8)	121.7	5.47, brs	122.5
10a	2.85, dd (14.4, 4.8)	33.3	2.92, dd (14.4, 4.2)	34.3	5′′		146.8		146.9
10b	2.69, dd (14.4, 9.6)		2.62, dd (14.4, 9.6)		6′′	5.98, d (9.6)	125.9	5.93, d (9.6)	125.4
11	1.10, d (6.6)	14.0	1.15, d (6.6)	14.1	7′′	6.26, d (9.6)	126.2	6.25, d (9.6)	126.7
12a	5.32, s	114.7	5.32, s	114.6	8′′		124.7		124.8
12b	5.13, s		5.13, s		9′′	2.12, m	46.1	2.10, m	45.6
13	6.31, dd (14.4, 9.6)	128.8	6.25, dd (14.4, 9.6)	128.7	10′′		35.9		35.9
14	5.39, ddd (14.4, 10.8, 2.4)	135.8	5.37, ddd (14.4, 11.4, 2.4)	135.8	11′′a	1.64, m	19.7	1.68, m	19.3
15a	2.49, m	41.1	2.48, m	41.2	11′′b	1.57, m		1.60, m	
15b	2.12, m		2.12, m		12′′a	2.05, m	36.7	2.07, m	36.5
16	2.80, m	33.7	2.80, m	33.7	12′′b	1.31, m		1.31, m	
17	6.19, d (9.6)	149.4	6.18, d (9.6)	149.3	13′′		43.9		43.8
18		134.9		134.7	14′′		150.6		150.7
19		203.7		203.6	15′′a	2.44, m	25.3	2.44, m	25.2
20	4.78, m	71.5	4.75, m	71.6	15′′b	2.35, m		2.34, m	
20-OH	3.74		3.72		16′′a	1.80, m	28.0	1.80, m	28.0
21a	1.91, m	31.6	1.88, m	31.6	16′′b	1.48, m		1.48, m	
21b	1.91, m		1.85, m		17′′	1.24, m	56.2	1.24, m	56.1
22a	2.92, m	37.6	2.86, m	37.5	18′′	0.96, s	19.4	1.05, s	18.6
22b	2.70, m		2.53, m		19′′	0.95, s	18.3	0.97, s	19.4
23		208.0		208.1	20′′	2.14, m	39.6	2.14, m	39.5
24	1.06, d (6.6)	20.1	1.06, d (6.6)	20.1	21′′	1.06, d (6.6)	21.4	1.06, d (6.6)	21.4
25	1.87, s	12.4	1.86, s	12.4	22′′	5.24, dd (15.6, 7.2)	135.4	5.23, dd (15.0, 7.2)	135.4
1′	7.89, brs				23′′	5.24, dd (15.6, 7.2)	132.4	5.24, dd (15.0, 7.2)	132.4
1′a		134.8		136.2	24′′	1.88, m	43.0	1.88, m	43.0
2′		140.2	6.98, s	124.6	25′′	1.49, m	33.3	1.49, m	33.3
3′		105.6		110.0	26′′	0.84, d (6.6)	19.8	0.84, d (6.6)	19.8
3′a		128.5		127.9	27′′	0.85, d (6.6)	20.1	0.85, d (6.6)	20.1
4′	7.40, d (7.8)	118.1	7.46, d (7.8)	118.8	28′′	0.94, d (6.6)	17.8	0.94, d (6.6)	17.8

**Fig. 3:**
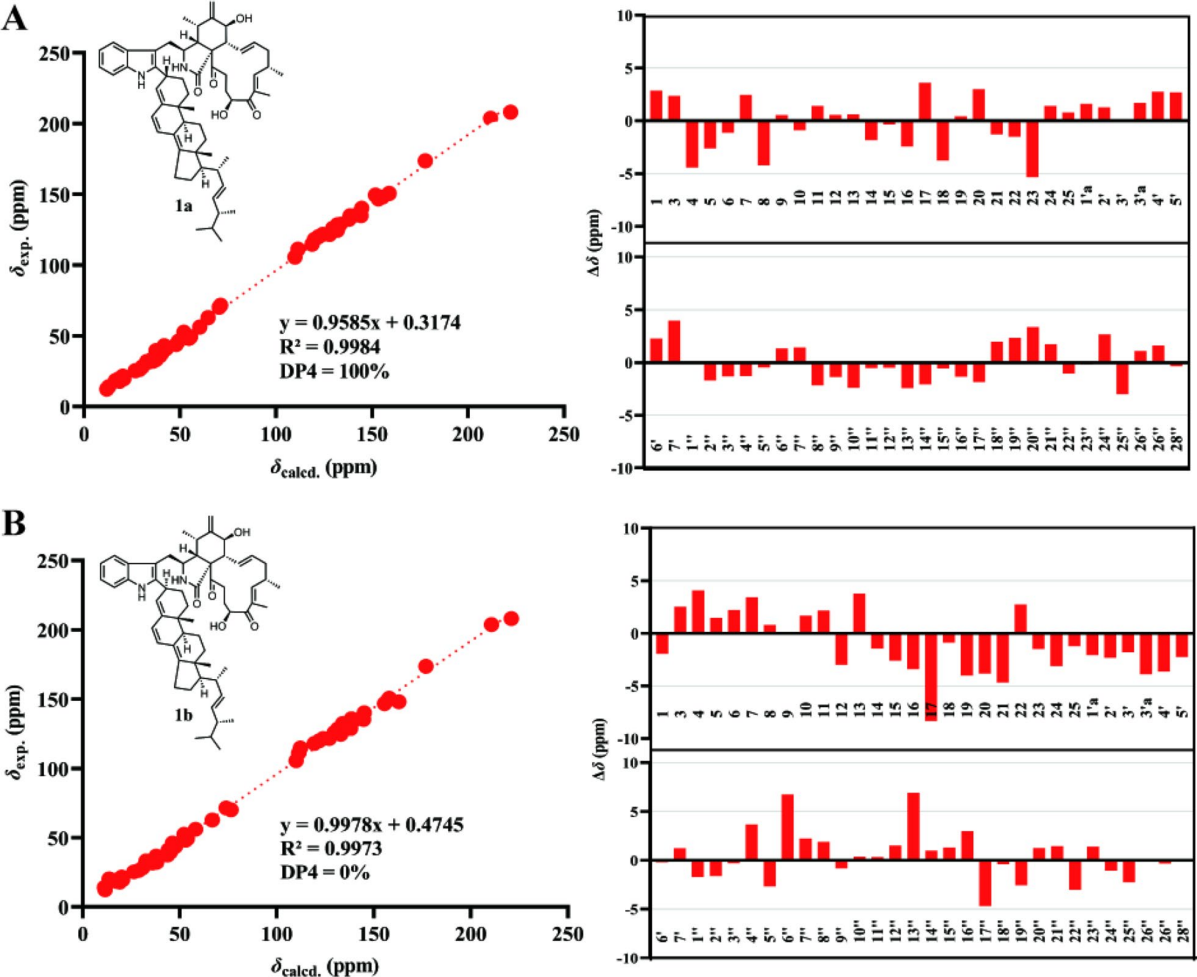
**A** Linear Regression analysis, DP4 analysis and subtraction of the experimental and calculated ^13^C NMR chemical shifts of **1a**. **B** Linear Regression analysis, DP4 analysis and subtraction of the experimental and calculated ^13^C NMR chemical shifts of **1b**

To further evaluate the rationality of the planar structure of **1**, *gauge*-independent atomic orbital (GIAO) calculations were performed to predict the ^13^C NMR chemical shifts of a model featuring a linkage between C-2′ of chaetoglobosin Fex and C-3′′ of ergosta-4,6,8(14),22-tetraen-3*β*-ol. As C-3′′ was a stereogenic center, two configurations were considered, and the ^13^C NMR chemical shifts of both were calculated. Linear regression analysis of the calculated versus experimental ^13^C chemical shifts, together with deviation analysis (Fig. 3), indicated that the C-2′/C-3′′ linkage pattern determined by 1D and 2D NMR data was reasonable. In this mode, the calculated ^13^C NMR data showed good agreement with the experimental results. For **1a**, the correlation coefficient (*R*^2^) was 0.9984, and all absolute deviations (|∆*δ*|) were within 5.40 ppm. Moreover, the key positions C-2′ and C-3′′ exhibited minimal deviations, with |∆*δ*| values below 1.30 ppm, suggesting that **1** was more likely to adopt the **1a** structure.

The relative configuration of **unit A** was comprehensively assessed through coupling constants, NOESY correlations, and chemical shifts. The coupling constant between H-13 and H-14 in **unit A** exceeded 12 Hz, supporting an *E*-configuration for the *Δ*^13^ double bond. The NOESY correlation between H-8 and H-14 further supported this assignment. The NOESY correlation between H-16 and H_3_-25 indicated that the *Δ*^17^ double bond also adopted an *E*-configuration. A series of NOESY correlations involving H-3/H_3_-11, H_3_-11/H-7, H-7/H-13, H-13/H-22a, and H-22a/H-20 suggested that these protons shared the same spatial orientation. Likewise, NOESY cross-peaks observed between H-4/H-8, H-8/H-14, and H-14/H-16 indicated that these protons also adopted a common orientation. To further assign the relative configuration at C-16 and C-20, the comparison of the NMR data (Table S1) between **1** and **9** revealed that the chemical shifts of H-20, C-16, and C-20 were generally similar (|Δ*δ*_H_|≤ 0.1 ppm, |Δ*δ*_C_|≤ 0.3 ppm). Comparison with **7** showed that the chemical shifts of H-16, H-20, C-16, and C-20 in **1** were similar to those in **7** (|Δ*δ*_H_|≤ 0.06 ppm, |Δ*δ*_C_|≤ 0.3 ppm). These similar chemical shifts suggested that **1**, **7**, and **9** shared closely related structures and configurations. Therefore, the relative configuration of **unit A** was assigned as 3S*, 4R*, 5S*, 7S*, 8R*, 16S*, and 20S*. The configuration of **unit B** in **1** was assessed based on coupling constant analysis and NOESY data. The large coupling constant between H-22′′ and H-23′′ suggested that the *Δ*^22′′^ double bond adopted an *E*-configuration. NOESY correlations between H-11′′b and both H_3_-18′′ and H_3_-19′′ implied that the methyl groups at C-10′′ and C-13′′ were located on the same face. The NOESY correlations of H-9″/H-12″b and H-12″a/H_3_-18″ indicated that H-9″ and H_3_-18″ were oriented on opposite faces. The NOESY correlations of H-15″a with H-17″, and H-15″b with H_3_-18″ indicated that H-17″ and H_3_-18″ were oriented on opposite faces. Due to the conformational flexibility of the side chain in **unit B**, the relative configurations at C-20′′ and C-24′′ could not be accurately determined based on NOESY data alone. Due to the lack of key NOESY correlations for C-3″, its relative configuration could not be reliably determined. Except for C-3″, C-20″, and C-24″, the relative configuration of **unit B** was determined as 9″R*, 10″R*, 13″R*, and 17″R*.

Among the reported chaetoglobosins derived from *Chaetomium* species [31–34], the configurations at C-3, C-4, C-8, C-9, and C-16 were relatively conserved as 3*S*, 4*R*, 8*R*, 9*R*, and 16*S*, respectively. When a methyl group was attached at C-5, it typically exhibited the *S*-configuration. Similarly, the presence of a hydroxyl group at C-7 was generally associated with the *S*-configuration. In most cases, the hydroxyl substitution at C-20 displayed an *S*-configuration. Only a few reports described an *R*-configuration at C-20, which notably affected the chemical shifts of C-20 and its neighboring atoms.

Based on the relative configuration of **1**, as well as the characteristic structural analogy to related compounds derived from *Chaetomium* species, the absolute configuration of **unit A** was confirmed as 3*S*, 4*R*, 5*S*, 7*S*, 8*R*, 9*R*, 16*S*, and 20*S*. To determine the absolute configuration of **unit B**, its chemical shifts were compared (Table S2) with those of the monomer ergosta-4,6,8(14),22-tetraen-3*β*-ol [30]. The side chain from C-20′′ to C-28′′ exhibited closely matching chemical shifts (|Δ*δ*_H_|≤ 0.1 ppm), indicating that the stereocenters at C-20′′ and C-24′′ likely possessed the same configurations. A similar comparison between **1** and **7** also showed comparable chemical shifts for the side chain in **unit B** (|Δ*δ*_H_|≤ 0.1 ppm). Furthermore, a structural feature comparison of ergosterol and its derivatives derived from *Chaetomium* species revealed that those bearing chemically identical side chains consistently adopted *R*-configurations at C-20 and C-24, while *S*-configured analogues have not been widely reported in the literature [31–34]. Thus, both of the absolute configurations at C-20′′ and C-24′′ in **unit B** were tentatively assigned as *R*-configuration. Based on the relative configuration of **unit B** and structural analogies of the chiral centers in ergosterol and its derivatives derived from *Chaetomium* species, the absolute configuration of **unit B**, excluding C-3″, was assigned as 9′′*R*, 10′′*R*, 13′′*R*, 17′′*R*, 20′′*R*, and 24′′*R*.

**Fig. 4:**
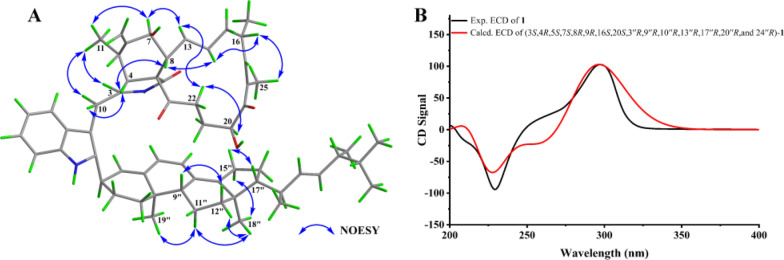
**A** Key NOESY correlations and **B** experimental versus calculated ECD spectra of **1**

To further evaluate the configuration at C-3′′, DP4 analysis was performed for the two possible isomers. The DP4 results (Fig. 3) indicated that the population of **1a** was 100%, whereas that of **1b** was 0%, suggesting that the configuration at C-3′′ in **1** was *R* configuration. Subsequently, density functional theory (DFT) calculations at the B3LYP/6–311 + G(d,p) level in methanol were carried out for (3*S*, 4*R*, 5*S*, 7*S*, 8*R*, 9*R*, 16*S*, 20*S*, 3′′*R*, 9′′*R*, 10′′*R*, 13′′*R*, 17′′*R*, 20′′*R*, and 24′′*R*)-**1**, and the calculated ECD spectrum was compared with the experimental one. The results (Fig. 4) showed that the calculated and experimental ECD spectra exhibited good agreement. The absolute configuration of **1** was thus assigned as 3*S*, 4*R*, 5*S*, 7*S*, 8*R*, 9*R*, 16*S*, 20*S*, 3′′*R*, 9′′*R*, 10′′*R*, 13′′*R*, 17′′*R*, 20′′*R*, and 24′′*R*.

**Fig. 5:**
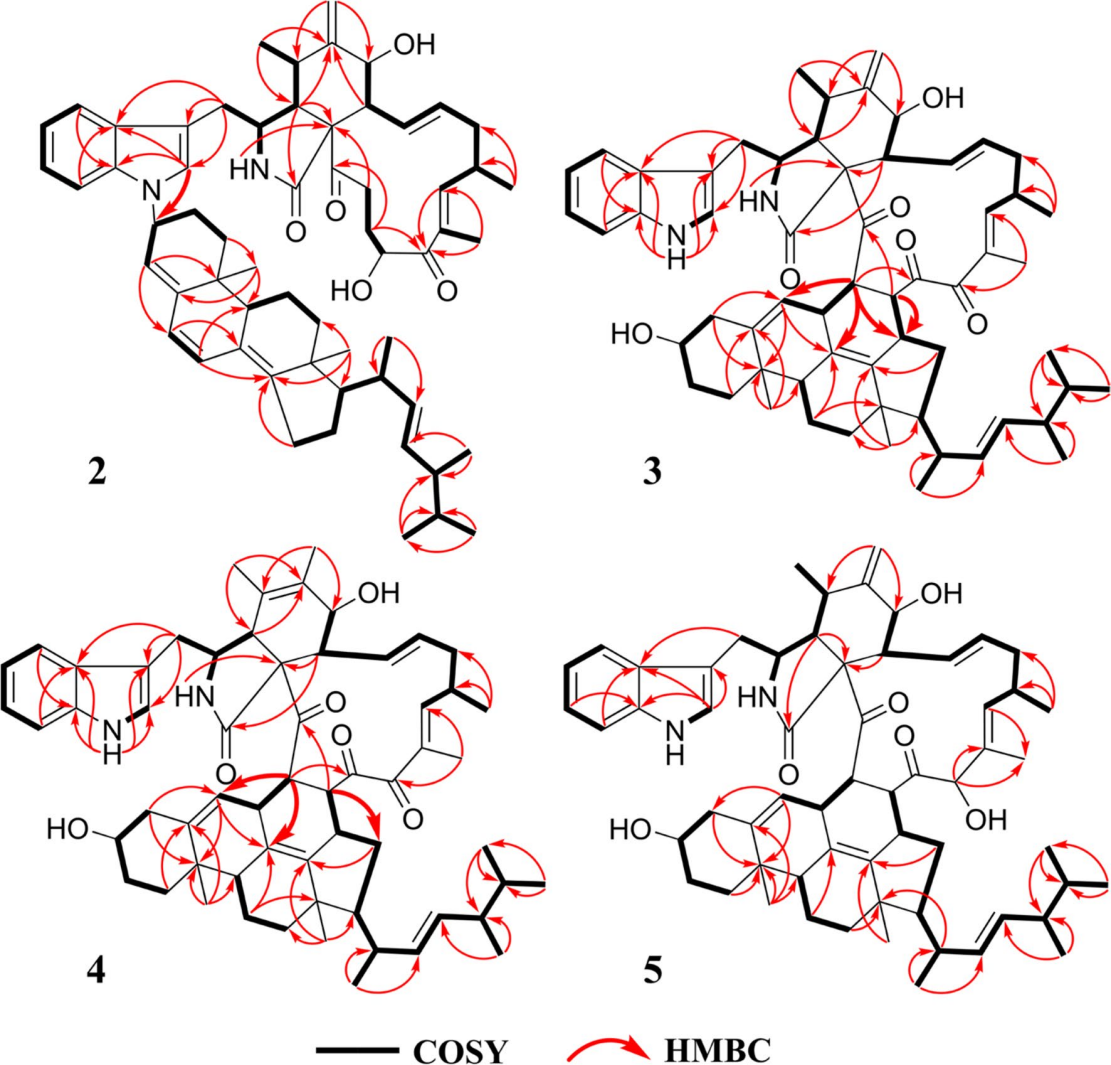
The ^1^H-^1^H COSY and key HMBC correlations of **2**–**5**

Chalasoergodimer B (**2**) was isolated as a brown powder. The molecular formula was determined to be C_60_H_78_N_2_O_5_, indicating 23 degrees of unsaturation, based on the HRESIMS ion peak at *m/z* 929.5811 [M + Na]^+^ (C_60_H_78_N_2_O_5_Na^+^, calcd. for 929.5808). The NMR data of **2** (Table 1) closely resembled those of **1**, except for differences in the indolyl moiety. Other signals, including two carbonyl groups, one amide group, two hydroxyl groups, and seven double bonds (including one terminal double bond), were consistent with those of **1**. These findings suggested that **2** was also formed from chaetoglobosin Fex and ergosta-4,6,8(14),22-tetraen-3*β*-ol, but exhibited a distinct substitution pattern compared to **1**. Comparison of the indole proton signals revealed an additional signal at *δ*_H_ 6.98 (s, 1H) in **2**, and lacked the proton signal for NH-1′, indicating that the substitution occurred at N-1′ rather than C-2′. This was further supported by the key HMBC correlation from H-2′ to C-3′′ (Fig. 5), which confirmed that the substitution took place at the NH-1′ position of the indolyl unit. Except for the connecting site and its neighboring region, compounds **1** and **2** generally exhibited similar 1D and 2D NMR signals in other regions.

**Fig. 6:**
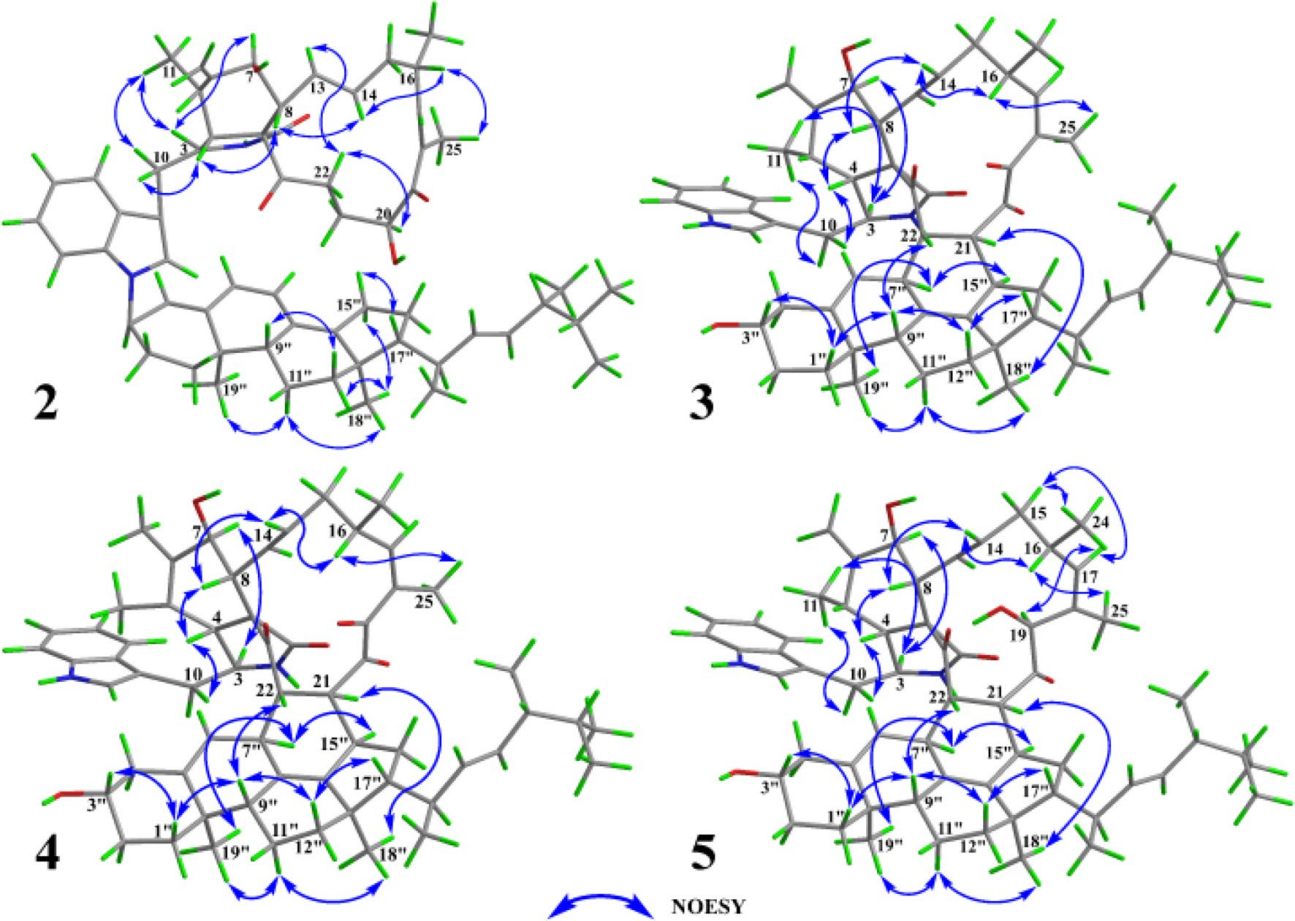
Key NOESY correlations of **2**–**5**

**Fig. 7:**
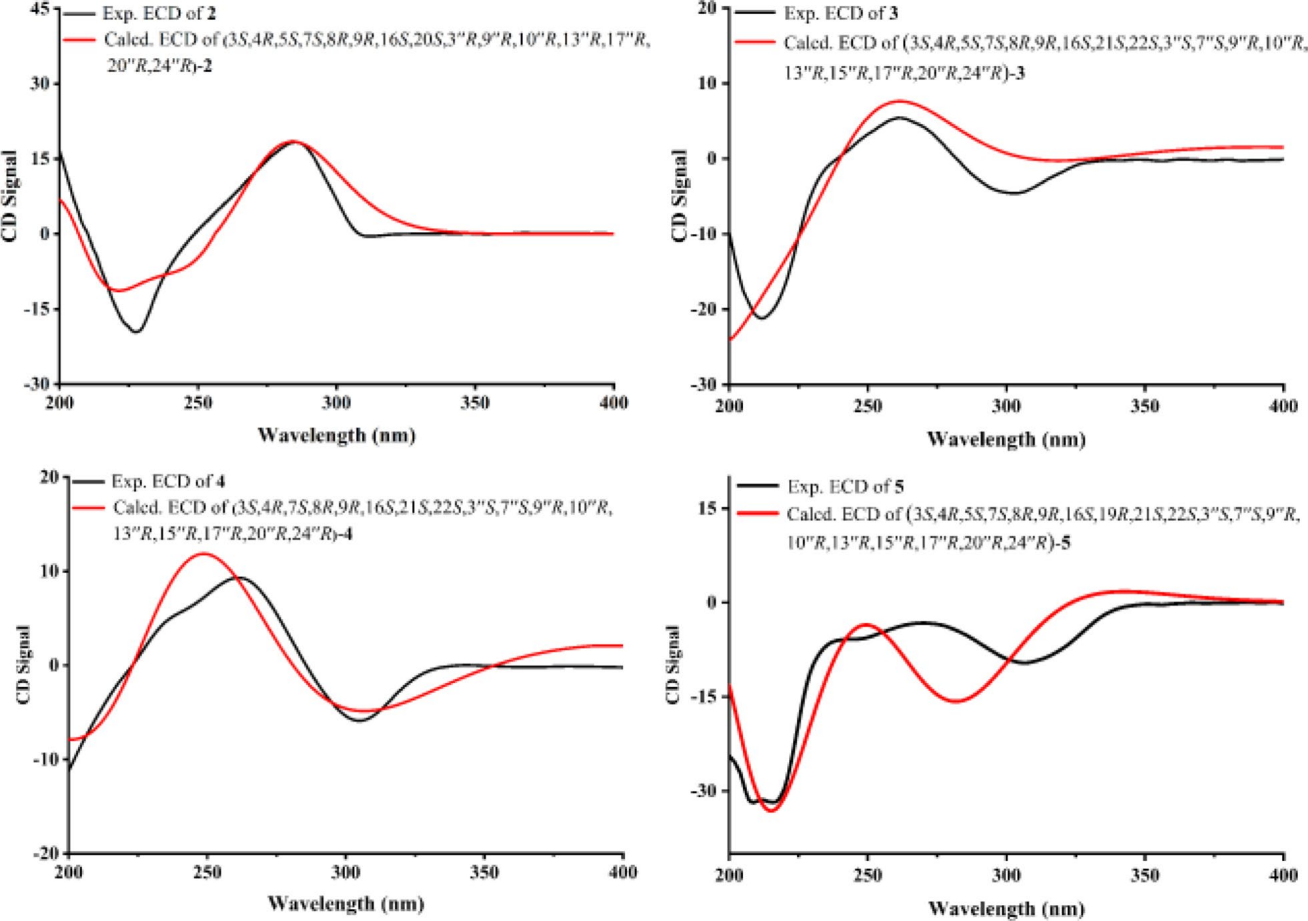
Experimental versus calculated ECD spectra of **2**–**5**

Compound **2** was suggested to possess the same monomeric composition as **1**, but with a different dimerization pattern. Given that **2** exhibited several identical NOESY correlations (Fig. 6), closely similar chemical shifts, and comparable coupling constants to those of **1**, it was considered reasonable to infer that the difference in dimerization mode did not significantly affect the relative or absolute configurations of the structure, except for C-3″. Therefore, **2** was considered to share the same relative and absolute configurations as **1** except the dimerization sites. The absolute configuration of **2**, excluding C-3″, was suggested as 3*S*, 4*R*, 5*S*, 7*S*, 8*R*, 9*R*, 16*S*, 20*S*, 9′′*R*, 10′′*R*, 13′′*R*, 17′′*R*, 20′′*R*, and 24′′*R*. The C-3′′ configuration of **2** was determined using the same approach as for **1**. DP4 analysis was performed to evaluate the distribution of the two possible configurations at the C-3′′ stereocenter. The results (Fig. S31) indicated that **2** was assigned 100% to the 3′′*R* configuration and 0% to the 3′′*S* configuration. Subsequently, theoretical ECD spectra of (3*S*, 4*R*, 5*S*, 7*S*, 8*R*, 9*R*, 16*S*, 20*S*, 3′′*R*, 9′′*R*, 10′′*R*, 13′′*R*, 17′′*R*, 20′′*R*, and 24′′*R*)-**2** was calculated and compared with the experimental ECD spectrum of **2**. As shown in Fig. 7, the calculated spectrum of the (3*S*, 4*R*, 5*S*, 7*S*, 8*R*, 9*R*, 16*S*, 20*S*, 3′′*R*, 9′′*R*, 10′′*R*, 13′′*R*, 17′′*R*, 20′′*R*, and 24′′*R*)-**2** showed better agreement with the experimental data, supporting the assignment of the absolute configuration of **2** as 3*S*, 4*R*, 5*S*, 7*S*, 8*R*, 9*R*, 16*S*, 20*S*, 3′′*R*, 9′′*R*, 10′′*R*, 13′′*R*, 17′′*R*, 20′′*R*, and 24′′*R*.

Chalasoergodimer C (**3**) was obtained as a white powder. The molecular formula was determined as C_60_H_76_N_2_O_6_, corresponding to 24 degrees of unsaturation, based on the HRESIMS ion peak at *m*/*z* 943.5598 [M + Na]^+^ (C_60_H_76_N_2_O_6_Na^+^, calcd. for 943.5601). The ^1^H, ^13^C, and HSQC spectra (Table 2 and Fig. S38) exhibited features characteristic of chaetoglobosin-ergosterol derivative hybrid. The NMR data revealed the presence of two terminal olefinic protons at *δ*_H_ 5.20 (s, 1H) and 5.42 (s, 1H), three carbonyl carbons at *δ*_C_ 198.4, 206.6, and 214.3, one amide carbonyl at *δ*_C_ 172.7. Compared to chaetoglobosin D (**11**), compound **3** lacked the C-21 double bond and the hydroxyl group at C-19 was replaced by a carbonyl group. Key HMBC correlations from H-21 to C-15′′ and from H-22 to C-6′′, C-8′′, and C-15′′, along with critical ^1^H-^1^H COSY cross-peaks between H-21/H-15′′ and H-22/H-7′′ (Fig. 5), supported a dimeric structure comprising chaetoglobosin D and 14-dehydroergosterol [35], connected via C-21 to C-15′′ and C-22 to C-7′′.

**Table 2:** ^1^H (600 MHz) and ^13^C (150 MHz) NMR data of compounds **3** and **4** in CDCl_3_

No	3		4		No	3		4	
	*δ*_H_ (*J* in Hz)	*δ*_C_	*δ*_H_ (*J* in Hz)	*δ*_C_		*δ*_H_ (*J* in Hz)	*δ*_C_	*δ*_H_ (*J* in Hz)	*δ*_C_
1		172.7		173.4	7′	7.41, d (7.8)	111.7	7.40, d (7.8)	111.6
2	5.63, brs		5.73, brs		1′′a	1.70, m	35.4	1.71, m	35.5
3	3.40, dt (11.4, 4.2)	52.5	3.44, m	58.6	1′′b	1.33, m		1.33, m	
4	2.43, dd (4.2, 4.2)	50.7	3.00, m	52.4	2′′a	1.88, m	32.1	1.88, m	32.1
5	3.08, m	32.9		127.1	2′′b	1.44, m		1.46, m	
6		148.5		131.7	3′′	3.69, m	70.4	3.67, m	70.4
7	4.08, d (10.8)	69.0	3.98, d (9.6)	67.8	4′′a	2.37, m	42.2	2.39, m	42.2
8	3.06, dd (10.8, 9.0)	49.5	2.47, dd (9.6, 9.6)	53.2	4′′b	2.20, m		2.21, m	
9		62.8		62.4	5′′		144.5		144.2
10a	3.02, dd (15.0, 6.0)	35.8	3.04, dd (13.8, 4.8)	34.5	6′′	5.19, m	120.7	5.20, m	121.0
10b	2.69, dd (15.0, 11.4)		2.84, dd (13.8, 9.6)		7′′	2.98, m	37.1	3.08, m	36.2
11	1.24, d (6.6)	14.7	1.59, s	17.9	8′′		125.8		125.5
12a	5.42, s	114.9	1.74, s	14.0	9′′	1.92, m	46.1	1.95, m	46.1
12b	5.20, s				10′′		36.5		36.4
13	5.92, dd (14.4, 9.0)	129.1	6.12, dd (14.4, 9.0)	129.3	11′′a	1.57, m	19.6	1.56, m	19.7
14	5.60, ddd (14.4, 7.8, 4.2)	132.9	5.64, ddd (14.4, 7.8, 4.2)	134.5	11′′b	1.49, m		1.48, m	
15a	2.34, m	40.9	2.36, m	41.1	12′′a	1.94, m	38.7	1.96, m	38.7
15b	2.23, m		2.22, m		12′′b	1.48, m		1.47, m	
16	2.79, m	32.9	2.79, m	32.8	13′′		42.5		42.5
17	6.61, d (7.8)	153.9	6.43, d (7.8)	154.3	14′′		145.8		145.6
18		134.1		134.1	15′′	2.54, m	35.0	2.59, m	34.9
19		198.4		198.4	16′′a	1.95, m	38.5	1.89, m	38.9
20		206.6		206.9	16′′b	1.48, m		1.67, m	
21	3.64, dd (10.8, 4.8)	51.0	3.72, dd (8.4, 4.2)	51.0	17′′	1.70, m	54.6	1.67, m	54.4
22	3.67, dd (10.8, 4.8)	52.2	3.66, dd (8.4, 4.2)	52.1	18′′	0.97, s	20.1	0.94, s	20.3
23		214.3		214.1	19′′	0.84, s	18.7	0.84, s	18.6
24	1.02, d (6.6)	19.0	1.07, d (6.6)	18.9	20′′	2.05, m	40.1	1.99, m	40.2
25	1.82, s	11.4	1.82, s	11.3	21′′	1.03, d (6.6)	21.5	1.01, d (6.0)	21.4
1′	8.20, brs		8.18, brs		22′′	5.19, m	136.1	5.20, dd (15.0, 10.2)	136.0
1′a		136.7		136.7	23′′	5.02, dd (15.0, 9.0)	132.3	5.03, dd (15.0, 8.4)	132.2
2′	7.10, d (1.8)	122.5	7.10, d (1.8)	122.5	24′′	1.57, m	43.1	1.64, m	43.0
3′		112.1		111.8	25′′	1.26, m	33.2	1.32, m	33.2
3′a		126.9		126.9	26′′	0.67, d (6.6)	19.8	0.73, d (6.6)	19.8
4′	7.53, d (7.8)	118.7	7.52, d (7.8)	118.6	27′′	0.68, d (6.6)	20.1	0.73, d (6.6)	20.2
5′	7.19, dd (7.8, 7.8)	120.2	7.16, dd (7.8, 7.8)	120.1	28′′	0.68, d (6.6)	18.5	0.72, d (6.6)	18.3
6′	7.24, dd (7.8, 7.8)	122.8	7.23, dd (7.8, 7.8)	122.8					

Based on the coupling constant between H-13 and H-14 and the NOESY correlation between H-8 and H-14, the double bond at C-13 was assigned as *E*-configuration. The NOESY correlation between H-16 and the methyl group H_3_-25 indicated that the double bond at C-16 was also *E*-configuration. The NOESY spectrum of **3** (Fig. 6) revealed key correlations, including H-3/H-7 and H-3/H-11, indicating that protons H-3, H-7, and H-11 were co-facial. Similarly, correlations of H-4/H-8, H-8/H-14, and H-14/H-16 supported the same orientation for protons H-4, H-8, and H-16. NOESY cross-peaks of H-1′′a/H-3′′, H-1′′a/H-9′′, H-22/H-9′′, H-9′′/H-12′′b, and H-12′′b/H-17′′ suggested that H-22, H-3′′, H-9′′, and H-17′′ shared the same spatial orientation. Additional correlations of H-7′′/H-15′′, H-7′′/H-19′′, H-11′′b/H-19′′, H-11′′b/H-18′′, and H-21/H-18′′ indicated that the protons at H-21, H-7′′, H-15′′, H-18′′, and H-19′′ were positioned on the same side. The coupling constants and NOESY correlations both indicated that **3** and **6** shared a highly similar configuration. Based on the established NOESY correlations and the similar chemical characteristics between **3** and **6**, the relative configuration of **3** was assigned as 3S*, 4R*, 5S*, 7S*, 8R*, 9R*, 16S*, 21S*, 22S*, 3′′S*, 7′′S*, 9′′R*, 10′′R*, 13′′R*, 15′′R*, 17′′R*, 20′′R*, and 24′′R*.

Based on the relative configurations, and in combination with structural feature comparisons of chaetoglobosins and ergosterol derivatives derived from *Chaetomium* species, the absolute configuration of **3** was determined as 3*S*, 4*R*, 5*S*, 7*S*, 8*R*, 9*R*, 16*S*, 21*S*, 22*S*, 3′′*S*, 7′′*S*, 9′′*R*, 10′′*R*, 13′′*R*, 15′′*R*, 17′′*R*, 20′′*R*, and 24′′*R*. Subsequently, ECD calculations were performed on (3*S*, 4*R*, 5*S*, 7*S*, 8*R*, 9*R*, 16*S*, 21*S*, 22*S*, 3′′*S*, 7′′*S*, 9′′*R*, 10′′*R*, 13′′*R*, 15′′*R*, 17′′*R*, 20′′*R*, and 24′′*R*)-**3**, and the results were compared with the experimental ECD spectrum of **3**. The comparison (Fig. 7) showed good agreement between the calculated and experimental data, supporting the assignment of the absolute configuration of **3** as 3*S*, 4*R*, 5*S*, 7*S*, 8*R*, 9*R*, 16*S*, 21*S*, 22*S*, 3′′*S*, 7′′*S*, 9′′*R*, 10′′*R*, 13′′*R*, 15′′*R*, 17′′*R*, 20′′*R*, and 24′′*R*.

Chalasoergodimer D (**4**) was isolated as a white powder. The molecular formula was determined as C_60_H_76_N_2_O_6_, corresponding to 24 degrees of unsaturation, based on the HRESIMS ion peak at *m*/*z* 943.5579 [M + Na]^+^ (C_60_H_76_N_2_O_6_Na^+^, calcd. for 943.5601). The ^1^H and ^13^C NMR signals (Table 2) of **4** were similar to **3**. By comparison, the structure of **4** lacked the terminal alkene signal but displayed an additional methyl resonance at *δ*_H_ 1.74 (s, 3H). Key HMBC correlations from H-11 to C-4, C-5, and C-6, and from H-12 to C-5, C-6, and C-7 (Fig. 5), supported the presence of a double bond between C-5 and C-6, each substituted with a methyl group. Given the absence of significant differences in other structural elements, the planar structure of **4** was determined accordingly. Compound **4** exhibited highly similar NOESY correlations and coupling constants to those of **3**. Since they shared the similar planar structure and chemical shifts overall, this suggested that they possessed similar configurations. Therefore, except for C-5, compound **4** was assigned the same relative configuration as **3**. Based on the relative configurations, and in combination with structural feature comparisons of chaetoglobosins and ergosterol derivatives derived from *Chaetomium* species, the absolute configuration of **4** was suggested as 3*S*, 4*R*, 7*S*, 8*R*, 9*R*, 16*S*, 21*S*, 22*S*, 3′′*S*, 7′′*S*, 9′′*R*, 10′′*R*, 13′′*R*, 15′′*R*, 17′′*R*, 20′′*R*, and 24′′*R*. Moreover, the excellent agreement between the calculated and experimental ECD spectra (Fig. 7) supported the assignment of the absolute configuration of **4** as 3*S*, 4*R*, 7*S*, 8*R*, 9*R*, 16*S*, 21*S*, 22*S*, 3′′*S*, 7′′*S*, 9′′*R*, 10′′*R*, 13′′*R*, 15′′*R*, 17′′*R*, 20′′*R*, and 24′′*R*.

Chalasoergodimer E (**5**) was isolated as a white powder. The molecular formula was determined as C_60_H_78_N_2_O_6_, corresponding to 23 degrees of unsaturation, based on the HRESIMS ion peak at *m*/*z* 945.5745 [M + Na]^+^ (C_60_H_78_N_2_O_6_Na^+^, calcd. for 945.5758). Analysis of the 1D (Table 3) and 2D NMR data of **5** (Fig. 5), in comparison with **3**, revealed that **5** and **3** shared highly similar structures, with the only difference being that **5** bore a hydroxyl group at C-19. Comparing of NOESY correlations (Fig. 6) and coupling constants confirmed that, except for C-19, the relative configuration of **5** was identical to that of **3**. The NOESY correlations of H-19 with H-17, H-17 with H-15b, and H-15b with H_3_-24 indicated that H-19 and H_3_-24 were oriented on same side. In addition, the similar chemical shifts of H-19 in **5** and **6** suggested comparable chemical environments, which further supported the evaluation that the relative configuration of C-19 was likely the same in **5** and **6**. Based on the relative configurations, and in combination with structural feature comparisons of chaetoglobosins and ergosterol derivatives derived from *Chaetomium* species, the absolute configuration of **5** was determined as 3*S*, 4*R*, 5*S*, 7*S*, 8*R*, 9*R*, 16*S*, 19*S*, 21*S*, 22*S*, 3′′*S*, 7′′*S*, 9′′*R*, 10′′*R*, 13′′*R*, 15′′*R*, 17′′*R*, 20′′*R*, and 24′′*R*. Moreover, the calculated ECD spectrum (Fig. 7) of (3*S*, 4*R*, 5*S*, 7*S*, 8*R*, 9*R*, 16*S*, 19*R*, 21*S*, 22*S*, 3′′*S*, 7′′*S*, 9′′*R*, 10′′*R*, 13′′*R*, 15′′*R*, 17′′*R*, 20′′*R*)-**5** exhibited good agreement with the experimental data of **5**, thereby confirmed its absolute configuration as 3*S*, 4*R*, 5*S*, 7*S*, 8*R*, 9*R*, 16*S*, 19*R*, 21*S*, 22*S*, 3′′*S*, 7′′*S*, 9′′*R*, 10′′*R*, 13′′*R*, 15′′*R*, 17′′*R*, 20′′*R*, and 24′′*R*.

**Table 3:** ^1^H (600 MHz) and ^13^C (150 MHz) NMR data of compound **5** in CDCl_3_

**No**	*δ*_H_ (*J* in Hz)	*δ*_C_	**No**	*δ*_H_ (*J* in Hz)	*δ*_C_
1		173.2	7′	7.36, d (7.8)	111.8
2	5.85, s		1′′a	1.77, dt (13.8, 3.0)	35.7
3	3.40, m	54.3	1′′b	1.33, m	
4	2.45, m	49.4	2′′a	1.91, m	32.0
5	3.01, m	32.7	2′′b	1.47, m	
6		148.0	3′′	3.65, m	70.2
7	3.81, d (11.4)	68.0	4′′a	2.56, m	41.9
8	2.69, m	49.0	4′′b	2.27, m	
9		62.7	5′′		146.0
10a	2.87, dd (12.6, 4.2)	33.9	6′′	5.39, m	122.3
10b	2.69, dd (12.6, 9.6)		7′′	2.98, m	33.2
11	1.24, m	14.2	8′′		129.0
12a	5.46, s	114.0	9′′	2.08, m	47.2
12b	5.17, s		10′′		36.6
13	5.85, m	126.5	11′′a	1.66, m	20.0
14	5.49, m	137.8	11′′b	1.66, m	
15a	2.26, m	41.9	12′′a	1.98, m	37.8
15b	1.95, m		12′′b	1.38, m	
16	2.53, m	33.2	13′′		42.4
17	5.26, d (7.8)	139.4	14′′		146.9
18		131.5	15′′	3.20, m	37.6
19	4.61, s	87.1	16′′a	1.87, m	33.9
20		211.9	16′′b	1.84, m	
21	3.20, m	47.6	17′′	0.78, m	54.3
22	2.87, m	48.9	18′′	0.99, s	20.8
23		214.9	19′′	0.96, s	17.8
24	1.02, d (6.6)	21.6	20′′	2.01, m	39.7
25	1.26, s	11.1	21′′	0.97, d (6.6)	20.8
1′	8.24, brs		22′′	5.12, dd (15.0, 9.0)	135.4
1′a		136.6	23′′	5.27,dd (15.0, 8.4)	132.8
2′	6.88, d (1.8)	122.7	24′′	1.90, m	42.7
3′		111.7	25′′	1.47, m	33.2
3′a		126.8	26′′	0.85, d (6.6)	20.0
4′	7.39, d (7.8)	118.6	27′′	0.86, d (6.6)	20.1
5′	7.10, dd (7.8, 7.8)	120.1	28′′	0.96, d (6.6)	17.8
6′	7.20, dd (7.8, 7.8)	122.7			

**Scheme 1.**
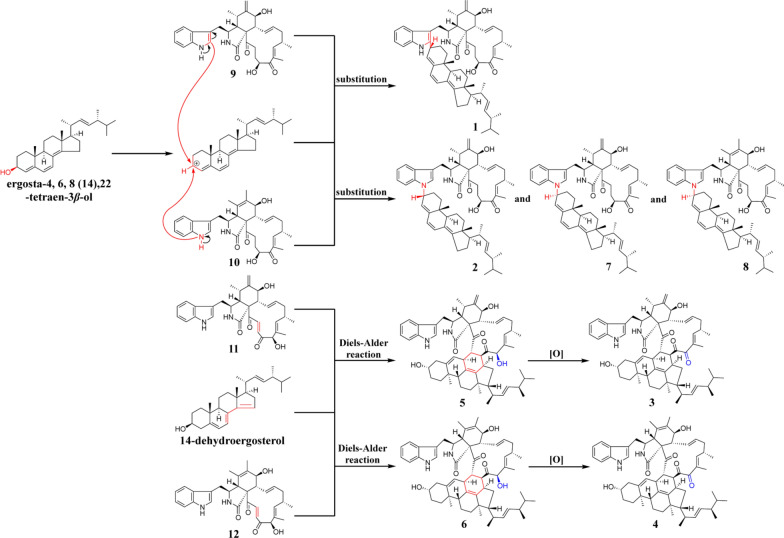
The proposed biosynthetic pathways of **1**–**12**

Based on the structures of compounds **1**–**12** and the currently reported studies on chaetoglobosin heterodimers [27, 36, 37], a possible biosynthetic pathway was proposed (Scheme 1). Compound **1** was likely formed via a substitution reaction between chaetoglobosin Fex (**9**) and ergosta-4,6,8(14),22-tetraen-3*β*-ol. The electron-rich C-2′ position of the indole ring rendered it particularly susceptible to nucleophilic substitution by electron-deficient species. Compounds **2** and **7** were presumably generated through substitution at the HN-1′ position of the indole moiety. Notably, compounds **1** and **2,** as well as **7,** exhibited opposite configurations at C-3′, suggesting a possible unimolecular nucleophilic substitution mechanism. Given the structural similarity between **7** and **8**, their dimerization was assumed to proceed via similar mechanisms. Compounds **5** and **6** likely arose from Diels–Alder cycloaddition reactions at C-21 and C-22 between chaetoglobosin D or B and 14-dehydroergosterol. In contrast, compounds **3** and **4** may originate from post-cyclization oxidation of **5** and **6**.

The possibility of an artificial reaction between chaetoglobosins and ergosterol analogues was evaluated. In these model experiments, cholesterol, which shared a similar structure with ergosta-4,6,8(14),22-tetraen-3*β*-ol, and chaetoglobosin Fex were used. Equimolar mixtures of the two compounds were stirred at 55 °C for 12 h in methanol:dichloromethane solvents (1:0, 1:1, and 0:1, v/v). Thin-layer chromatography (TLC) analysis of the resulting mixtures revealed no significant formation of new products. Due to the lack of available ergosterol derivatives structurally similar to 14-dehydroergosterol, further experimental evaluation of the Diels–Alder cycloaddition pathway could not be conducted. Given the rarity of enzymatic nucleophilic substitution involving alcohols at the C-1′ or C-2′ positions of indole rings, compounds **1**, **2**, **7** and **8** were more likely biosynthesized through non-enzymatic pathways. Although the Diels–Alder cycloaddition represents a well-known class of natural product cyclizations, only a limited number of enzymes capable of catalyzing this reaction have been identified. Thus, the formation of **3**, **4**, **5**, and **6** were also presumed to proceed via non-enzymatic mechanisms.

Chaetoglobosins have been reported to exhibit antitumor activity [38]. Accordingly, the cytotoxic activities of **1**–**12** against A549 cells were evaluated (Table S10). Compounds **9**–**12** showed notable inhibitory effects, with IC_50_ values of 9.26, 9.14, 14.89, and 5.14 μM. Cisplatin was selected as a positive control, with the IC₅₀ of 2.39 μM, slightly lower than that of compound **12**. In contrast, compounds **1**–**8** displayed no significant cytotoxicity, possibly due to the increased molecular weight and reduced polarity resulting from polymerization with ergosterol derivatives, which may have hindered their membrane permeability and subsequent bioactivity.

## Conclusion

New dimeric compounds with distinct polymerization patterns were identified, offering structure for active compound screening and advancing the understanding of potential dimerization modes between monomers. In this study, five new heterodimers (**1**–**5**), three known heterodimers (**6**–**8**), and four known chaetoglobosins (**9**–**12**) were isolated from the marine-derived fungus *Chaetomium* sp. Compound **1** featured a previously unreported polymerization mode between chaetoglobosin and ergosterol derivative, expanding the dimerization strategies of chaetoglobosin. Compound **2** possessed an *R*-configuration at the polymerization site, a structural feature not documented in related dimers. Cytotoxicity assays revealed that compounds **9**–**12** exhibited marked antitumor activity, with compound **12** showing the highest potency (IC_50_ = 5.14 μM).

## Materials and methods

### General experimental procedures

The OR results were measured using a JASCO P-2000 polarimeter. The ECD data were obtained with a MOS-450/SFM 300 instrument. The 1D and 2D NMR spectra were recorded on a Bruker Avance-III 600 MHz spectrometer. The HRESIMS spectra were acquired using a Thermo Scientific LTQ Orbitrap XL mass spectrometer. The analysis and preparation of compounds were carried out using a Shimadzu LC-60AD semi-preparative HPLC, connected to an SPD-20A photodiode array detector and a Waters C18 column (10 mm × 250 mm, 5 μm). Other column chromatographies included silica gel columns (200–300 mesh) and Sephadex LH-20 (18–110 μm).

### Fungal material and cultivation

The fungal strain used in this research was isolated from marine sediments of the Bohai Sea, China. It was identified as *Chaetomium* sp. based on ITS region amplification and sequencing (GenBank accession no. OR429403), and was preserved at Hebei University, China. The strain was initially cultured on potato dextrose agar (PDA) medium at 28°C for 5 days. Subsequently, the fungal mycelia were cut into small pieces and transferred into sterilized 1L Erlenmeyer flasks, each containing 200 g of rice and 170 mL of water, and further incubated at 28 °C for 30 days. A total of 255 fungal fermentation batches were successfully obtained through screening and statistical analysis.

### Isolation and purification

The fermentation product was extracted with a 1:1 (v/v) mixture of dichloromethane and methanol, yielding 2,491 g of crude extract. The extract was partitioned between ethyl acetate and water (1:1, v/v), and the organic phase was collected. The ethyl acetate phase was concentrated and dried to yield 1,259 g of crude extract. The ethyl acetate phase underwent silica gel column chromatography (CC), eluted stepwise with petroleum ether–ethyl acetate (80:20, 65:35, and 30:60, v/v), affording three primary fractions (Fr.1–Fr.3). Fraction 2 (Fr.2) was further purified over Sephadex LH-20 using petroleum ether–dichloromethane–methanol (2:1:1, v/v/v) to give subfractions Fr.2.1–Fr.2.4. Subfraction Fr.2.1 was subjected to silica gel CC and then semi-preparative HPLC on a Waters C18 column (100% MeOH) to afford compounds **1** (7.4 mg, 20.3 min), **2** (3.6 mg, 32.4 min), **7** (13.5 mg, 27.0 min), and **8** (9.1mg, 25.2 min). Subfraction Fr.2.2 was purified by Sephadex LH-20, followed by semi-preparative HPLC (100% MeOH) to yield compounds **3** (4.1 mg, 11.8 min), **4** (6.6 mg, 10.5 min), **5** (4.2 mg, 10.3 min), and **6** (5.4 mg, 12.5 min). Fraction 3 (Fr.3) was subjected to Sephadex LH-20 chromatography using a methanol–dichloromethane solvent system (1:1, v/v) to afford five subfractions (Fr.3.1–Fr.3.5). Subfraction Fr.3.2 was further purified by semi-preparative HPLC (55% MeCN/H_2_O) to yield compounds **9** (41.4 mg, 11.2 min) and **10** (31.9 mg, 12.9 min). Subfraction Fr.3.3 was purified by semi-preparative HPLC (50% MeCN/H_2_O) to afford compounds **11** (25.4 mg, 19.1 min) and **12** (35.3 mg, 20.5 min).

*Chalasoergodimer A* (**1**): brown power; $[\rm\alpha]^{25}_\text{D}$ + 269.9 (*c* 0.05, MeOH); UV (MeOH) *λ*_max_ (log *ε*) 226 (4.60), 291 (4.46) nm; ECD (0.55 mM, MeOH) *λ*_max_ (Δ*ε*) 229 (− 48.64), 297 (52.93) nm; ^1^H and ^13^C NMR data see Table 1; HRESIMS *m*/*z* 929.5818 [M + Na]^+^ (C_60_H_78_N_2_O_5_Na^+^, calcd. for 929.5808).

*Chalasoergodimer B* (**2**): brown power; $[\rm\alpha]^{25}_\text{D}$ + 64.6 (*c* 0.10, MeOH); UV (MeOH) *λ*_max_ (log *ε*) 226 (4.48), 291 (4.40) nm; ECD (0.55 mM, MeOH) *λ*_max_ (Δ*ε*) 227 (− 10.76), 285 (10.08) nm; ^1^H and ^13^C NMR data see Table 1; HRESIMS *m*/*z* 929.5811 [M + Na]^+^ (C_60_H_78_N_2_O_5_Na^+^, calcd. for 929.5808).

*Chalasoergodimer C* (**3**): white power; $[\rm\alpha]^{25}_\text{D}$ + 40.8 (*c* 0.10, MeOH); UV (MeOH) *λ*_max_ (log *ε*) 219 (4.56), 261 (3.91) nm; ECD (0.27 mM, MeOH) *λ*_max_ (Δ*ε*) 212 (− 21.21), 261 (5.41), 303 (− 4.60) nm; ^1^H and ^13^C NMR data see Table 2; HRESIMS *m*/*z* 943.5598 [M + Na]^+^ (C_60_H_76_N_2_O_6_Na^+^, calcd. for 943.5601).

*Chalasoergodimer D* (**4**): white power; $[\rm\alpha]^{25}_\text{D}$ + 104.06 (*c* 0.10, MeOH); UV (MeOH) *λ*_max_ (log *ε*) 220 (4.68), 260 (4.00) nm; ECD (0.27 mg/mL, MeOH) *λ*_max_ (Δ*ε*) 260 (10.46), 305 (− 6.73) nm; ^1^H and ^13^C NMR data see Table 2; HRESIMS *m*/*z* 943.5579 [M + Na]^+^ (C_60_H_76_N_2_O_6_Na^+^, calcd. for 943.5601).

*Chalasoergodimer E* (**5**): white power; $[\rm\alpha]^{25}_\text{D}$ − 126.37 (*c* 0.10, MeOH); UV (MeOH) *λ*_max_ (log *ε*) 220 (4.64), 282 (3.84) nm; ECD (0.17 mM, MeOH) *λ*_max_ (Δ*ε*) 209 (− 31.83), 216 (− 31.81), 307 (− 9.61) nm; ^1^H and ^13^C NMR data see Table 3; HRESIMS *m*/*z* 945.5745 [M + Na]^+^ (C_60_H_78_N_2_O_6_Na^+^, calcd. for 945.5758).

### Chemical calculations

The stereostructures of (3*S*, 4*R*, 5*S*, 7*S*, 8*R*, 9*R*, 16*S*, 20*S*, 3′′*R*, 9′′*R*, 10′′*R*, 13′′*R*, 17′′*R*, 20′′*R*, and 24′′*R*)-**1**, (3*S*, 4*R*, 5*S*, 7*S*, 8*R*, 9*R*, 16*S*, 20*S*, 3′′*S*, 9′′*R*, 10′′*R*, 13′′*R*, 17′′*R*, 20′′*R*, and 24′′*R*)-**1**, (3*S*, 4*R*, 5*S*, 7*S*, 8*R*, 9*R*, 16*S*, 20*S*, 3′′*R*, 9′′*R*, 10′′*R*, 13′′*R*, 17′′*R*, 20′′*R*, and 24′′*R*)-**2**, (3*S*, 4*R*, 5*S*, 7*S*, 8*R*, 9*R*, 16*S*, 20*S*, 3′′*S*, 9′′*R*, 10′′*R*, 13′′*R*, 17′′*R*, 20′′*R*, and 24′′*R*)-**2**, (3*S*, 4*R*, 5*S*, 7*S*, 8*R*, 9*R*, 16*S*, 21*S*, 22*S*, 3′′*S*, 7′′*S*, 9′′*R*, 10′′*R*, 13′′*R*, 15′′*R*, 17′′*R*, 20′′*R*, and 24′′*R*)-**3**, (3*S*, 4*R*, 7*S*, 8*R*, 9*R*, 16*S*, 21*S*, 22*S*, 3′′*S*, 7′′*S*, 9′′*R*, 10′′*R*, 13′′*R*, 15′′*R*, 17′′*R*, 20′′*R*, and 24′′*R*)-**4**, and (3*S*, 4*R*, 5*S*, 7*S*, 8*R*, 9*R*, 16*S*, 19*R*, 21*S*, 22*S*, 3′′*S*, 7′′*S*, 9′′*R*, 10′′*R*, 13′′*R*, 15′′*R*, 17′′*R*, 20′′*R*, and 24′′*R*)-**5** were constructed using GaussView software. Conformational searches were constructed with the MMFF94s force field in Compute VOA software, and conformers with relative energies within 5.0 kcal/mol were selected. Geometry optimizations were performed at the B3LYP/6-31G(d) level in Gaussian 09 [39]. Subsequently, ECD spectra and ^13^C NMR chemical shifts were calculated at the B3LYP/6-311 + G(d,p) level. The calculated results were averaged using Boltzmann-weighted populations and visualized with SpecDis 164 software [40].

### Biological activity evaluation

The cytotoxicity of compounds **1**–**12** against A549 cells was evaluated using the MTT assay [41]. Cells were seeded in 96-well plates at a density of 5 × 10^4^ cells/mL and incubated for 24 h. The test compounds were then added at various concentrations according to the experimental conditions, followed by further incubation. After 48 h, 10 μL of MTT solution (5 mg/mL, Biyotime, China) was added to each well, and the plates were incubated at 37 °C. After an additional 4 h, the culture medium was removed, and 100 μL of DMSO was added to each well. The absorbance was measured at 490 nm using a Multiskan FC microplate reader (Thermo Fisher Scientific, USA).

## Supplementary Information

Additional file 1. 1D and 2D NMR, HRESIMS of compounds **1–5**; The Cartesian coordinates of the lowest-energy conformers used in the computational studies of compounds **1–5**, **7**; Biological activity data of compounds **1–12** (PDF)

## Data Availability

The data generated in this study were included in the article and its supplementary materials. The NMR data of compounds **1**–**5** have been deposited in the Natural Products Magnetic Resonance Database (NP-MRD; www.np-mrd.org) and can be found at NP0351258 (Chalasoergodimer A), NP0351259 (Chalasoergodimer B), NP0351260 (Chalasoergodimer C), NP0351261 (Chalasoergodimer D), NP0351262 (Chalasoergodimer E).
