# Supplementary material for: Chalasoergodimers A–E, heterodimers with multiple polymerization modes from a marine-derived Chaetomium sp. fungus
Source: Nat Prod Bioprospect. 2025 Sep 11;15(1):62. doi: 10.1007/s13659-025-00544-5 (PMC12423002; doi:10.1007/s13659-025-00544-5)
Supplement: Supplementary file 1 — Additional file 1. 1D and 2D NMR, HRESIMS of compounds 1–5; The Cartesian coordinates of the lowest-energy conformers used in the computational studies of compounds 1–5, 7; Biological activity data of compounds 1–12 (PDF) [file 13659_2025_544_MOESM1_ESM.pdf]

Supplementary Material

Chalasoergodimers A–E, heterodimers with multiple polymerization modes from a marine-derived *Chaetomium* sp. fungus

Ze-Hong Lin[1], Han-Wen Shan[1,2], Li-Kun Yang[3*], Tian-Tian Sun[1], Li-Ying He[1,2], Hui-Fang Du[1], Ya-Hui Zhang[3], Shan Liu[1], Xu Wang[1], Du-Qiang Luo[3*] and Fei Cao[1*]

[1]College of Pharmaceutical Sciences, Key Laboratory of Medicinal Chemistry and Molecular Diagnostics of Education Ministry of China, State Key Laboratory of New Pharmaceutical Preparations and Excipients, Hebei University, Baoding 071002, People's Republic of China.
[2]Zhejiang Fonow Medicine Co., Ltd, Dongyang 322106, People's Republic of China.
[3]College of Life Sciences, Hebei University, Baoding 071002, People's Republic of China.

*Correspondence and requests for materials should be addressed to F. C. (caofei542927001@163.com), D.-Q. L. (duqiangluo@hbu.edu.cn) or L.-K. Y. (yanglikun@hbu.edu.cn)

List of Supporting Information

Figure S1. ^{1}H NMR (600 MHz, $CDCl_3$) spectrum of compound **1**
Figure S2. Partial enlarged ^{1}H NMR (600 MHz, $CDCl_3$) spectrum of compound **1**
Figure S3. Partial enlarged ^{1}H NMR (600 MHz, $CDCl_3$) spectrum of compound **1**
Figure S4. ^{13}C NMR (150 MHz, $CDCl_3$) spectrum of compound **1**
Figure S5. Partial enlarged ^{13}C NMR (150 MHz, $CDCl_3$) spectrum of compound **1**
Figure S6. Partial enlarged ^{13}C NMR (150 MHz, $CDCl_3$) spectrum of compound **1**
Figure S7. HSQC (600 MHz, $CDCl_3$) spectrum of compound **1**
Figure S8. Partial enlarged HSQC (600 MHz, $CDCl_3$) spectrum of compound **1**
Figure S9. ^{1}H-^{1}H COSY (600 MHz, $CDCl_3$) spectrum of compound **1**
Figure S10. Partial enlarged ^{1}H-^{1}H COSY (600 MHz, $CDCl_3$) spectrum of compound **1**
Figure S11. HMBC (600 MHz, $CDCl_3$) spectrum of compound **1**
Figure S12. Partial enlarged HMBC (600 MHz, $CDCl_3$) spectrum of compound **1**
Figure S13. NOESY (600 MHz, $CDCl_3$) spectrum of compound **1**
Figure S14. Partial enlarged NOESY (600 MHz, $CDCl_3$) spectrum of compound **1**
Figure S15. HRESIMS spectrum of compound **1**
Figure S16. ^{1}H NMR (600 MHz, $CDCl_3$) spectrum of compound **2**
Figure S17. Partial enlarged ^{1}H NMR (600 MHz, $CDCl_3$) spectrum of compound **2**
Figure S18. Partial enlarged ^{1}H NMR (600 MHz, $CDCl_3$) spectrum of compound **2**
Figure S19. ^{13}C NMR (150 MHz, $CDCl_3$) spectrum of compound **2**
Figure S20. Partial enlarged ^{13}C NMR (150 MHz, $CDCl_3$) spectrum of compound **2**
Figure S21. Partial enlarged ^{13}C NMR (150 MHz, $CDCl_3$) spectrum of compound **2**
Figure S22. HSQC (600 MHz, $CDCl_3$) spectrum of compound **2**
Figure S23. Partial enlarged HSQC (600 MHz, $CDCl_3$) spectrum of compound **2**
Figure S24. ^{1}H-^{1}H COSY (600 MHz, $CDCl_3$) spectrum of compound **2**
Figure S25. Partial enlarged ^{1}H-^{1}H COSY (600 MHz, $CDCl_3$) spectrum of compound **2**
Figure S26. HMBC (600 MHz, $CDCl_3$) spectrum of compound **2**
Figure S27. Partial enlarged HMBC (600 MHz, $CDCl_3$) spectrum of compound **2**
Figure S28. NOESY (600 MHz, $CDCl_3$) spectrum of compound **2**
Figure S29. Partial enlarged NOESY (600 MHz, $CDCl_3$) spectrum of compound **2**
Figure S30. HRESIMS spectrum of compound **2**
Figure S31. Linear Regression analysis and DP4 analysis of the experimental and calculated ^{13}C NMR chemical shifts of **2a** and **2b**
Figure S32. ^{1}H NMR (600 MHz, $CDCl_3$) spectrum of compound **3**
Figure S33. Partial enlarged ^{1}H NMR (600 MHz, $CDCl_3$) spectrum of compound **3**
Figure S34. Partial enlarged ^{1}H NMR (600 MHz, $CDCl_3$) spectrum of compound **3**
Figure S35. ^{13}C NMR (150 MHz, $CDCl_3$) spectrum of compound **3**
Figure S36. Partial enlarged ^{13}C NMR (150 MHz, $CDCl_3$) spectrum of compound **3**
Figure S37. Partial enlarged ^{13}C NMR (150 MHz, $CDCl_3$) spectrum of compound **3**
Figure S38. HSQC (600 MHz, $CDCl_3$) spectrum of compound **3**
Figure S39. Partial enlarged HSQC (600 MHz, $CDCl_3$) spectrum of compound **3**
Figure S40. ^{1}H-^{1}H COSY (600 MHz, $CDCl_3$) spectrum of compound **3**

Figure S41 Partial enlarged ^{1}H-^{1}H COSY (600 MHz, $CDCl_3$) spectrum of compound **3**
Figure S42. HMBC (600 MHz, $CDCl_3$) spectrum of compound **3**
Figure S43. Partial enlarged HMBC (600 MHz, $CDCl_3$) spectrum of compound **3**
Figure S44. NOESY (600 MHz, $CDCl_3$) spectrum of compound **3**
Figure S45. Partial enlarged NOESY (600 MHz, $CDCl_3$) spectrum of compound **3**
Figure S46. HRESIMS spectrum of compound **3**
Figure S47. ^{1}H NMR (600 MHz, $CDCl_3$) spectrum of compound **4**
Figure S48. Partial enlarged ^{1}H NMR (600 MHz, $CDCl_3$) spectrum of compound **4**
Figure S49. Partial enlarged ^{1}H NMR (600 MHz, $CDCl_3$) spectrum of compound **4**
Figure S50. ^{13}C NMR (150 MHz, $CDCl_3$) spectrum of compound **4**
Figure S51. Partial enlarged ^{13}C NMR (150 MHz, $CDCl_3$) spectrum of compound **4**
Figure S52. Partial enlarged ^{13}C NMR (150 MHz, $CDCl_3$) spectrum of compound **4**
Figure S53. HSQC (600 MHz, $CDCl_3$) spectrum of compound **4**
Figure S54. Partial enlarged HSQC (600 MHz, $CDCl_3$) spectrum of compound **4**
Figure S55. ^{1}H-^{1}H COSY (600 MHz, $CDCl_3$) spectrum of compound **4**
Figure S56. Partial enlarged ^{1}H-^{1}H COSY (600 MHz, $CDCl_3$) spectrum of compound **4**
Figure S57. HMBC (600 MHz, $CDCl_3$) spectrum of compound **4**
Figure S58. Partial enlarged HMBC (600 MHz, $CDCl_3$) spectrum of compound **4**
Figure S59. NOESY (600 MHz, $CDCl_3$) spectrum of compound **4**
Figure S60. Partial enlarged NOESY (600 MHz, $CDCl_3$) spectrum of compound **4**
Figure S61. HRESIMS spectrum of compound **4**
Figure S62. ^{1}H NMR (600 MHz, $CDCl_3$) spectrum of compound **5**
Figure S63. Partial enlarged ^{1}H NMR (600 MHz, $CDCl_3$) spectrum of compound **5**
Figure S64. Partial enlarged ^{1}H NMR (600 MHz, $CDCl_3$) spectrum of compound **5**
Figure S65. ^{13}C NMR (150 MHz, $CDCl_3$) spectrum of compound **5**
Figure S66. Partial enlarged ^{13}C NMR (150 MHz, $CDCl_3$) spectrum of compound **5**
Figure S67. Partial enlarged ^{13}C NMR (150 MHz, $CDCl_3$) spectrum of compound **5**
Figure S68. HSQC (600 MHz, $CDCl_3$) spectrum of compound **5**
Figure S69. Partial enlarged HSQC (600 MHz, $CDCl_3$) spectrum of compound **5**
Figure S70. ^{1}H-^{1}H COSY (600 MHz, $CDCl_3$) spectrum of compound **5**
Figure S71. Partial enlarged ^{1}H-^{1}H COSY (600 MHz, $CDCl_3$) spectrum of compound **5**
Figure S72. HMBC (600 MHz, $CDCl_3$) spectrum of compound **5**
Figure S73. Partial enlarged HMBC (600 MHz, $CDCl_3$) spectrum of compound **5**
Figure S74. NOESY (600 MHz, $CDCl_3$) spectrum of compound **5**
Figure S75. Partial enlarged NOESY (600 MHz, $CDCl_3$) spectrum of compound **5**
Figure S76. HRESIMS spectrum of compound **5**
Table S1. Comparison of ^{1}H and ^{13}C NMR data of the stereocenters and adjacent atoms of **1** with ergochaeglobosin E and chaetoglobosin Fex
Table S2. Comparison of ^{1}H and ^{13}C NMR data of the stereocenters and adjacent atoms of **1** with ergochaeglobosin E and ergosta-4,6,8(14),22-tetraen-3*β*-ol
Table S3. The coordinates for the lowest-energy conformer of **(**3″*R*)-**1** in calculation
Table S4. The coordinates for the lowest-energy conformer of **(**3″*S*)-**1** in calculation
Table S5. The coordinates for the lowest-energy conformer of **(**3″*R*)-**2** in calculation
Table S6. The coordinates for the lowest-energy conformer of **(**3″*S*)-**2** in calculation

Table S7. The coordinates for the lowest-energy conformer of (21*S*,22*S*)-**3** in calculation
Table S8. The coordinates for the lowest-energy conformer of (21*S*,22*S*)-**4** in calculation
Table S9. The coordinates for the lowest-energy conformer of (21*S*,22*S*)-**5** in calculation
Table S10. The cytotoxic activities of compounds **1–12** against human non-small cell lung cancer A549 cells

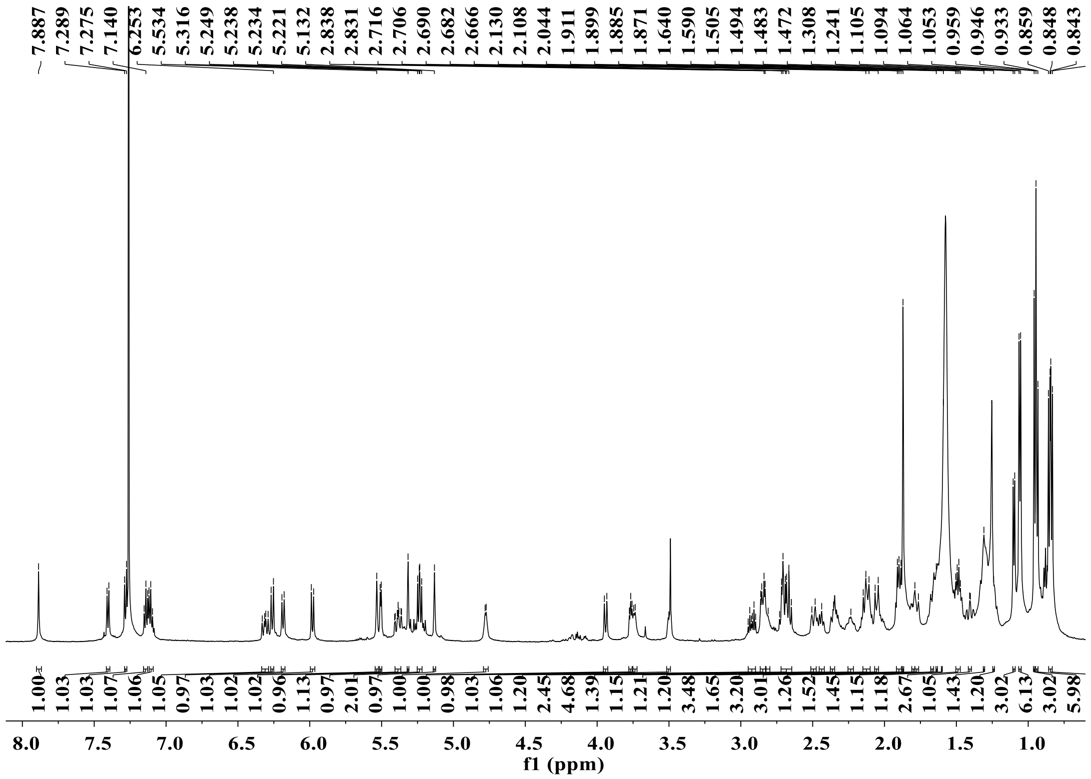

Figure S1. 1H NMR (600 MHz, $CDCl_3$) spectrum of compound **1**

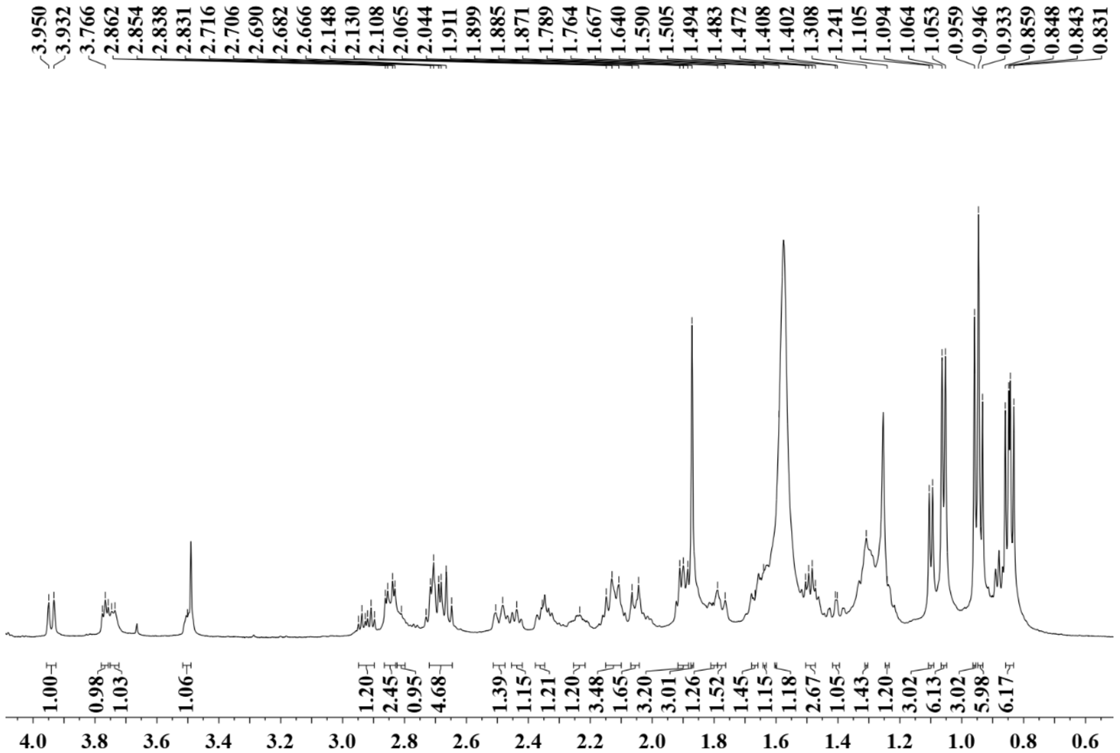

Figure S2. Partial enlarged ^{1}H NMR (600 MHz, $CDCl_3$) spectrum of compound **1**

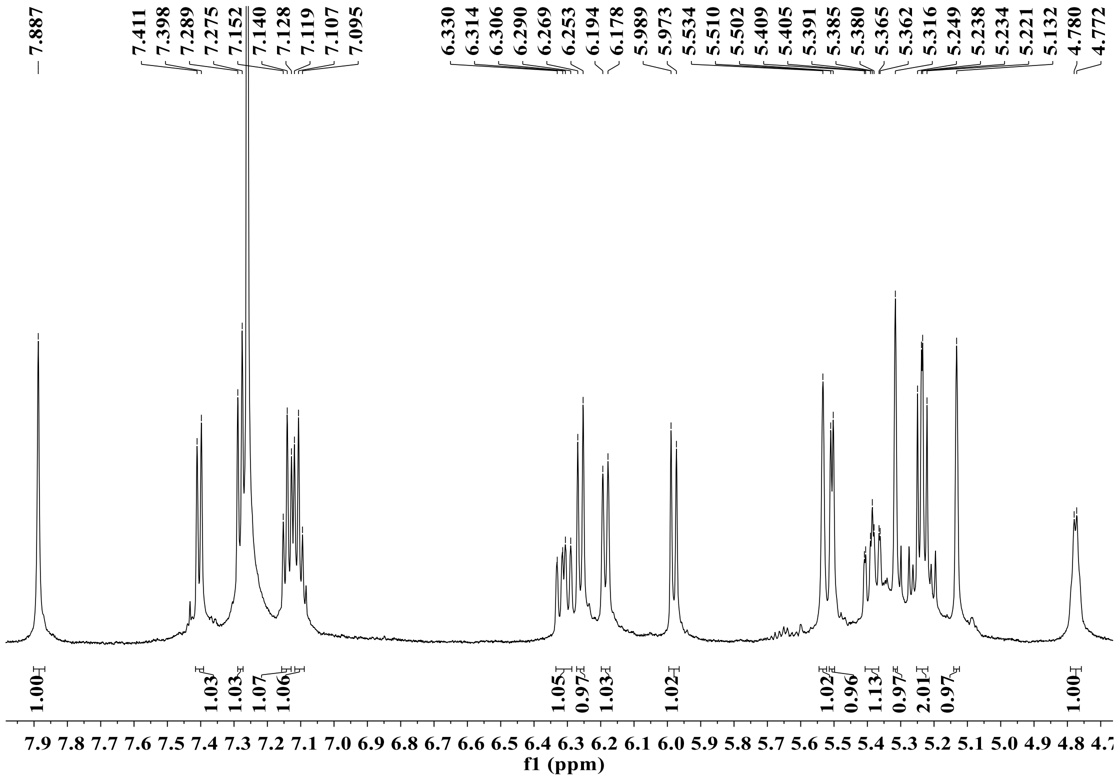

Figure S3. Partial enlarged ^{1}H NMR (600 MHz, $CDCl_3$) spectrum of compound **1**

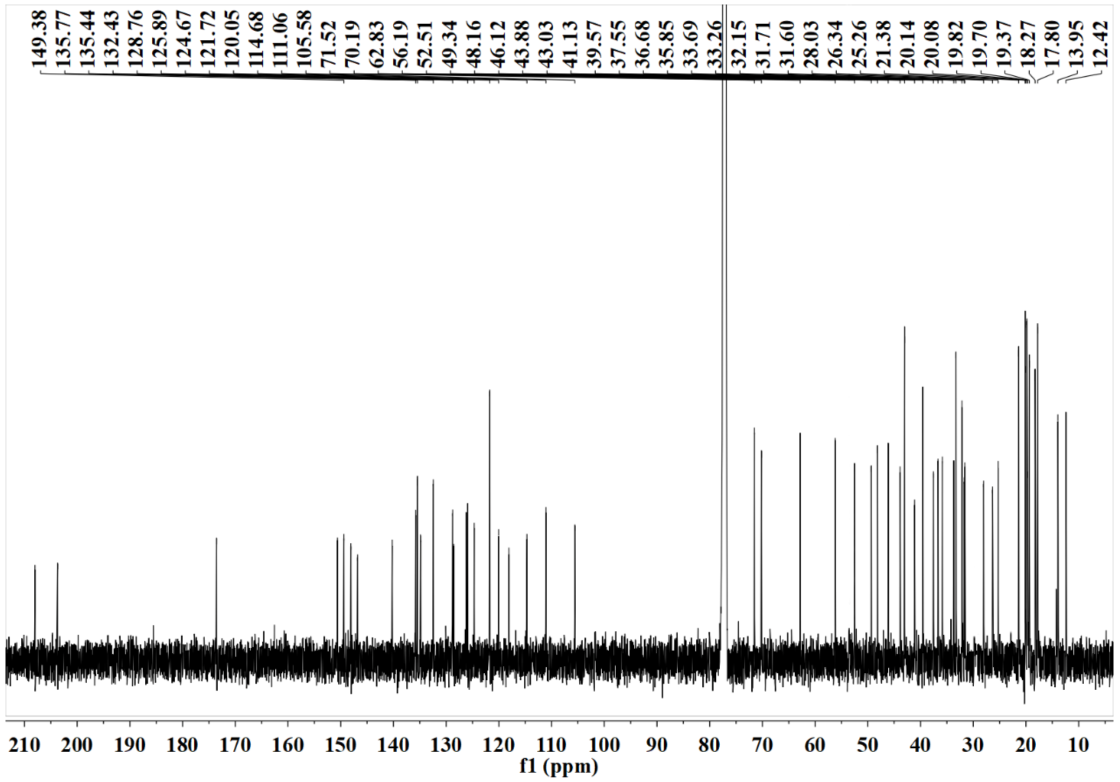

Figure S4. ^{13}C NMR (150 MHz, $CDCl_3$) spectrum of compound **1**

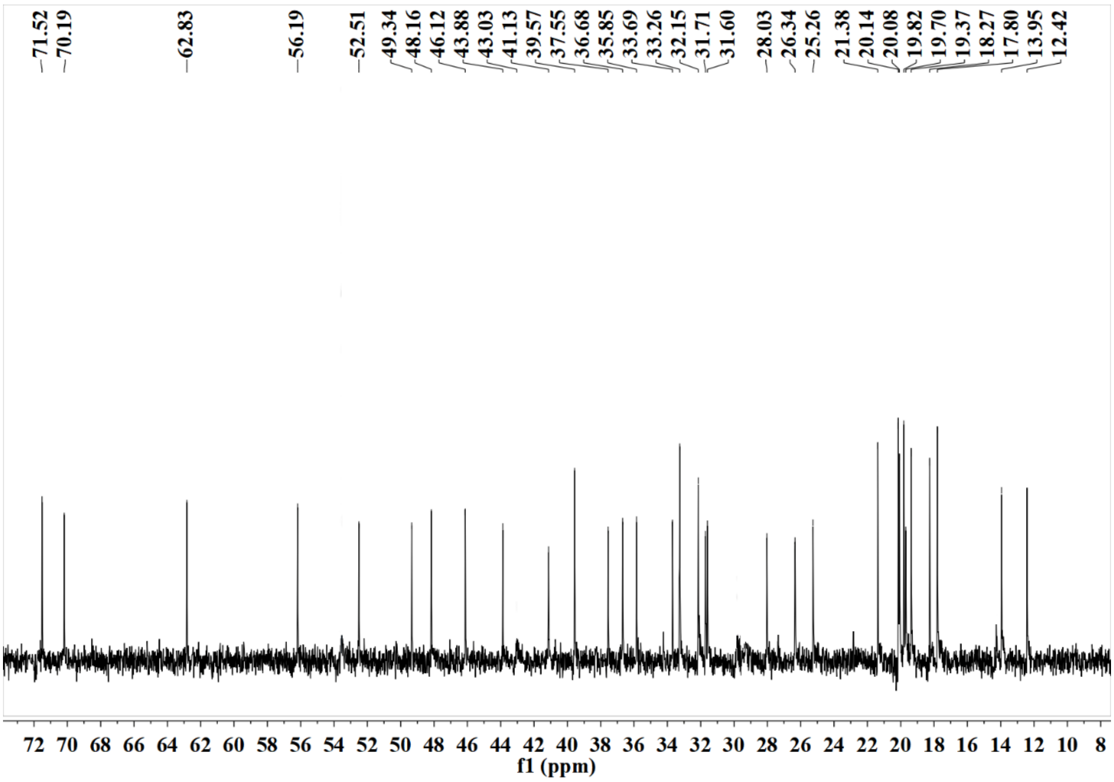

Figure S5. Partial enlarged ^{13}C NMR (150 MHz, $CDCl_3$) spectrum of compound **1**

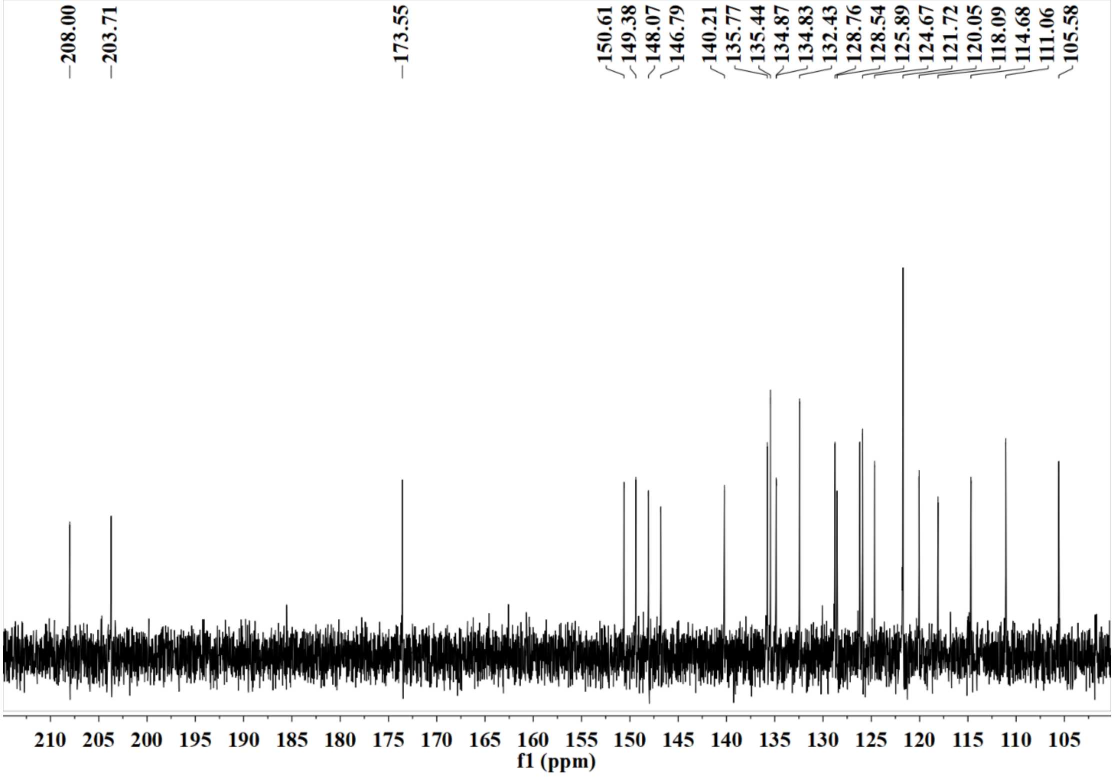

Figure S6. Partial enlarged ^{13}C NMR (150 MHz, $CDCl_3$) spectrum of compound **1**

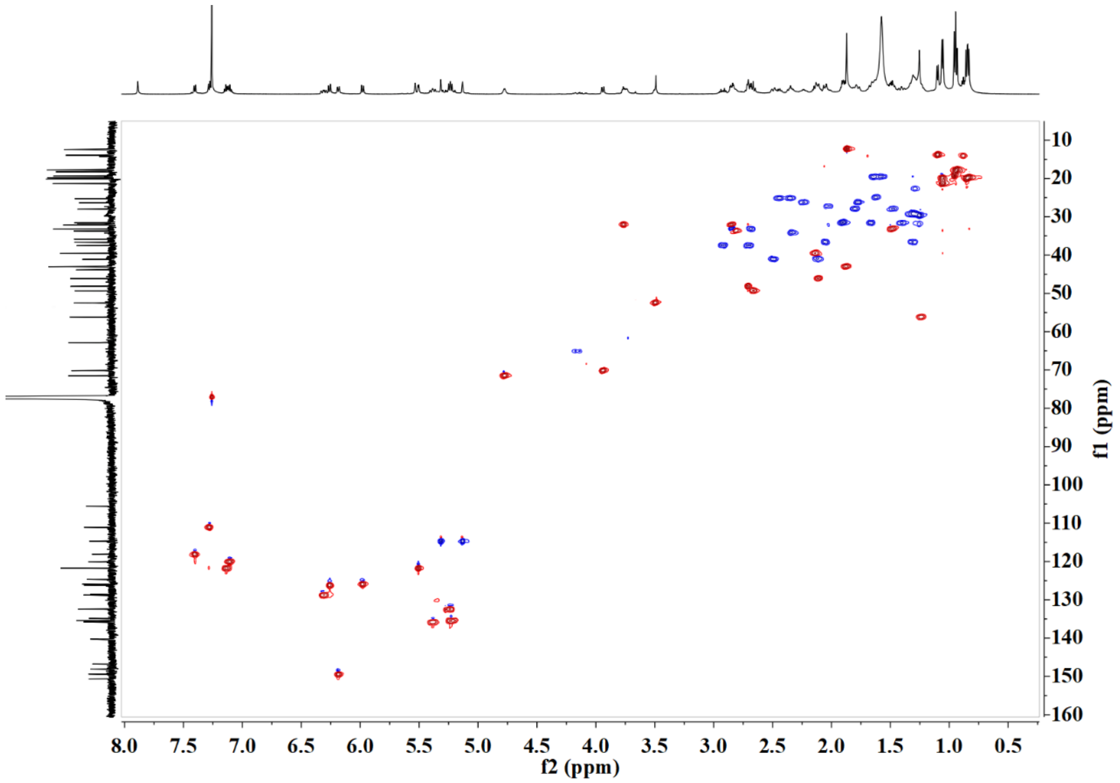

Figure S7. HSQC (600 MHz, $CDCl_3$) spectrum of compound **1**

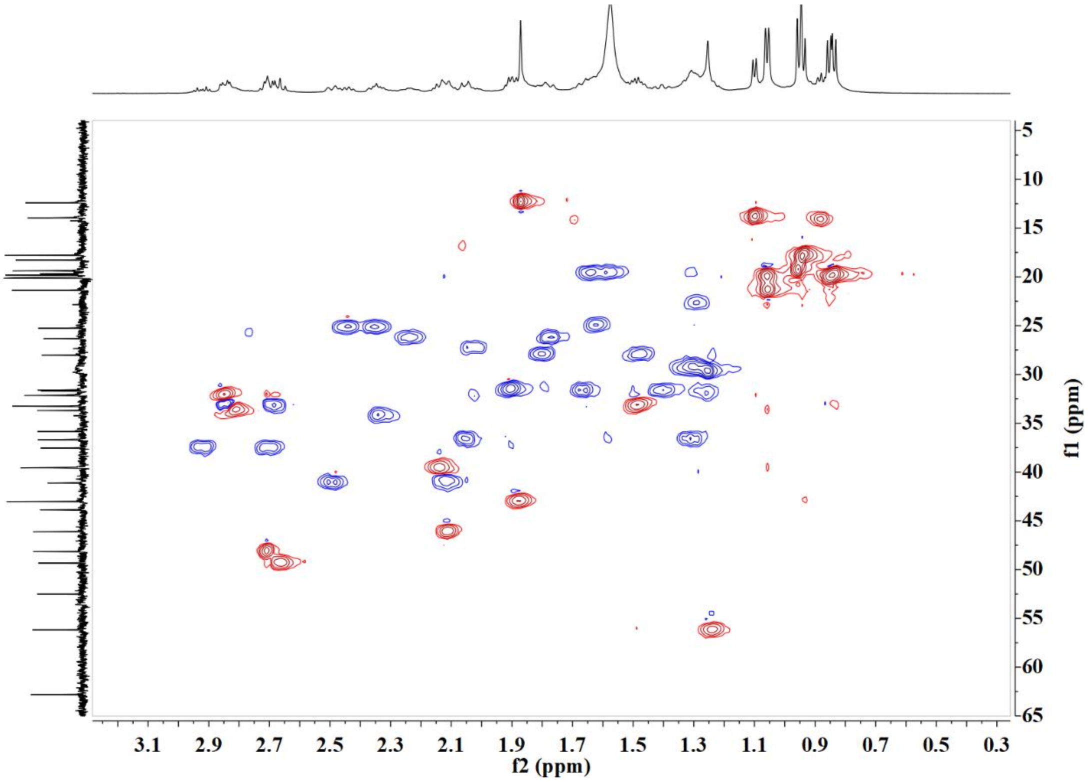

Figure S8. Partial enlarged HSQC (600 MHz, $CDCl_3$) spectrum of compound **1**

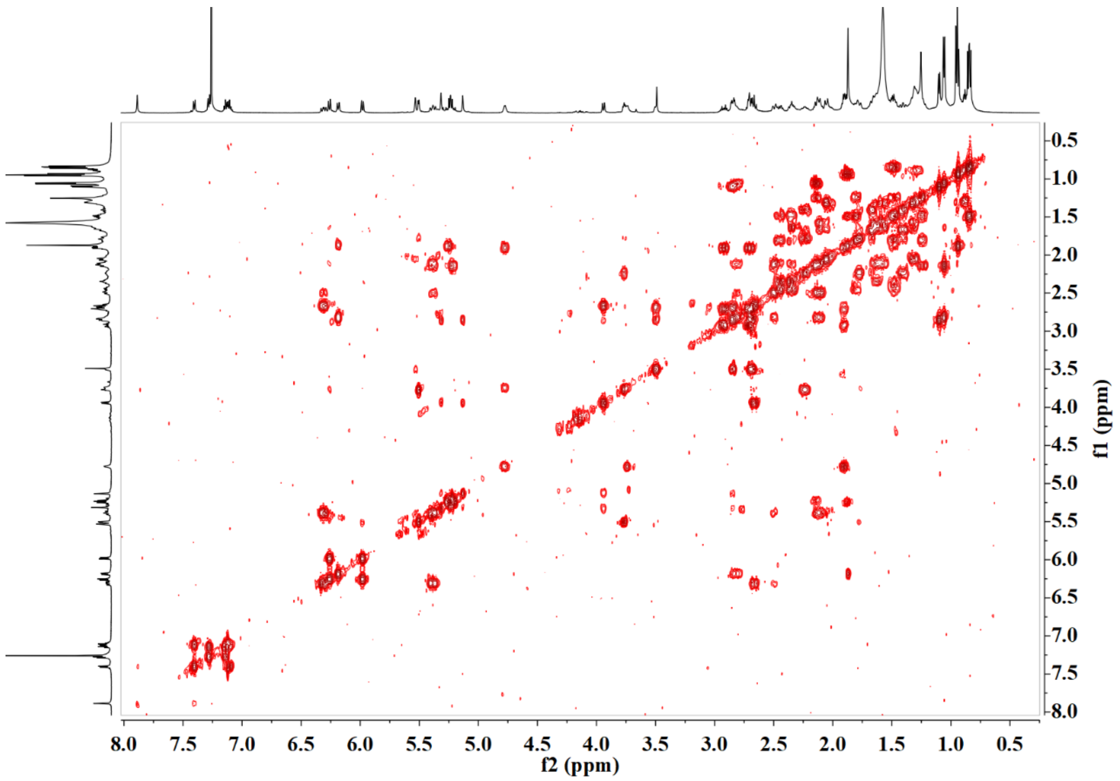

Figure S9. ^{1}H-^{1}H COSY (600 MHz, $CDCl_3$) spectrum of compound **1**

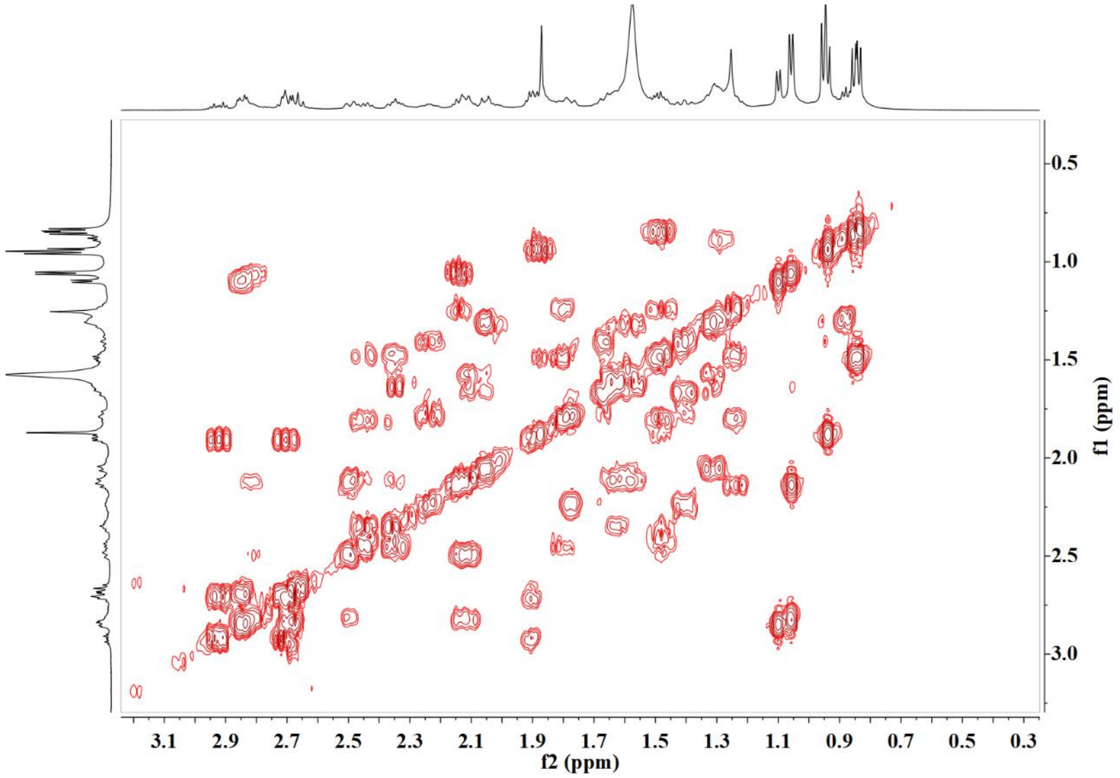

Figure S10. Partial enlarged ^{1}H-^{1}H COSY (600 MHz, $CDCl_3$) spectrum of compound **1**

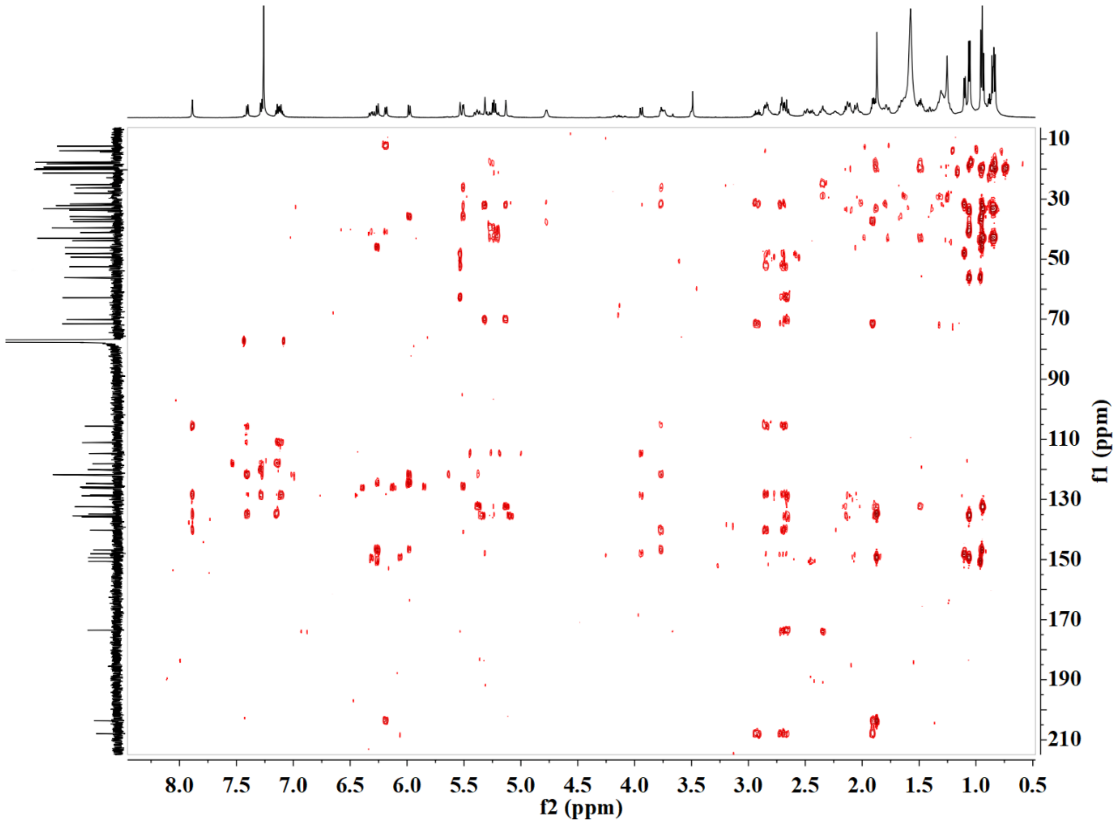

Figure S11. HMBC (600 MHz, $CDCl_3$) spectrum of compound **1**

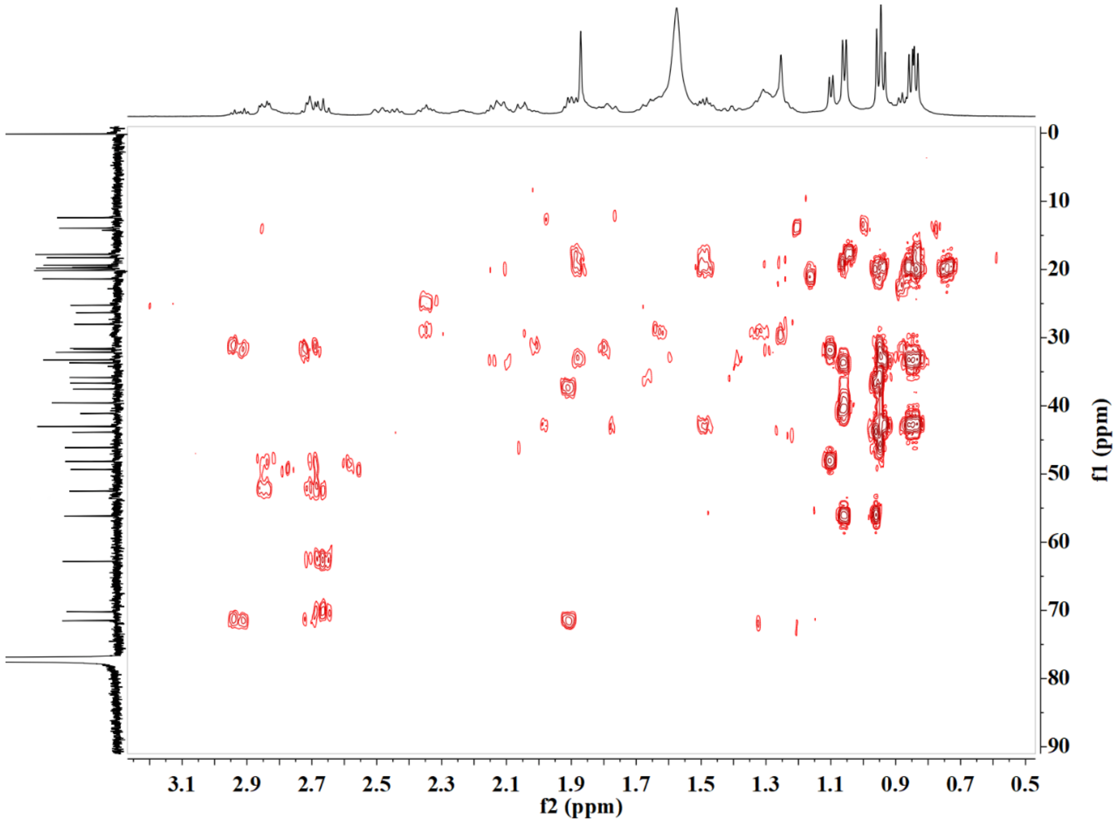

Figure S12. Partial enlarged HMBC (600 MHz, $CDCl_3$) spectrum of compound **1**

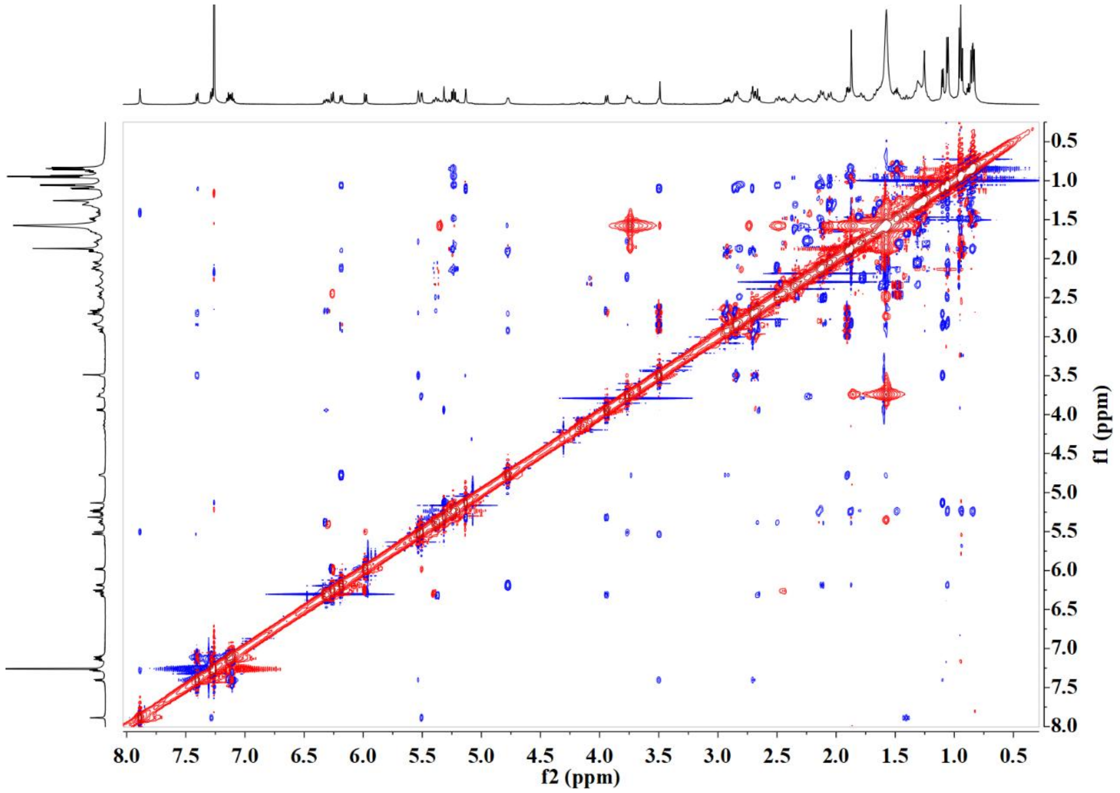

Figure S13. NOESY (600 MHz, $CDCl_3$) spectrum of compound **1**

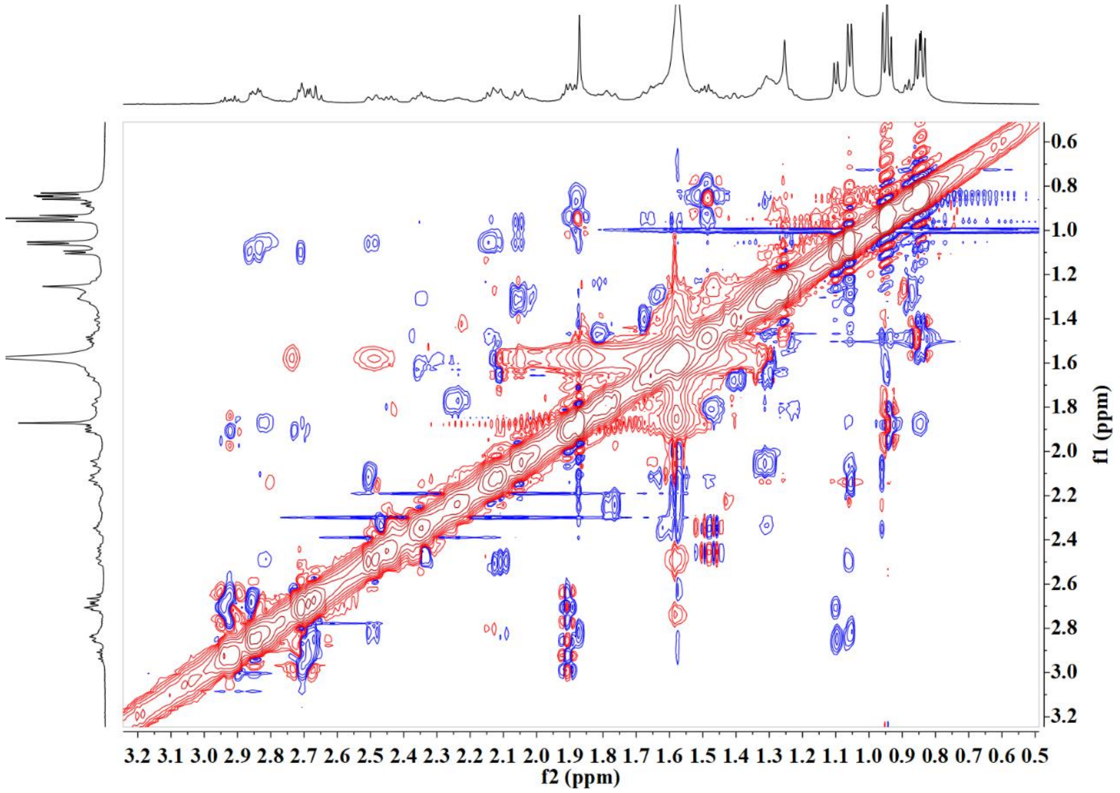

Figure S14. Partial enlarged NOESY (600 MHz, $CDCl_3$) spectrum of compound **1**

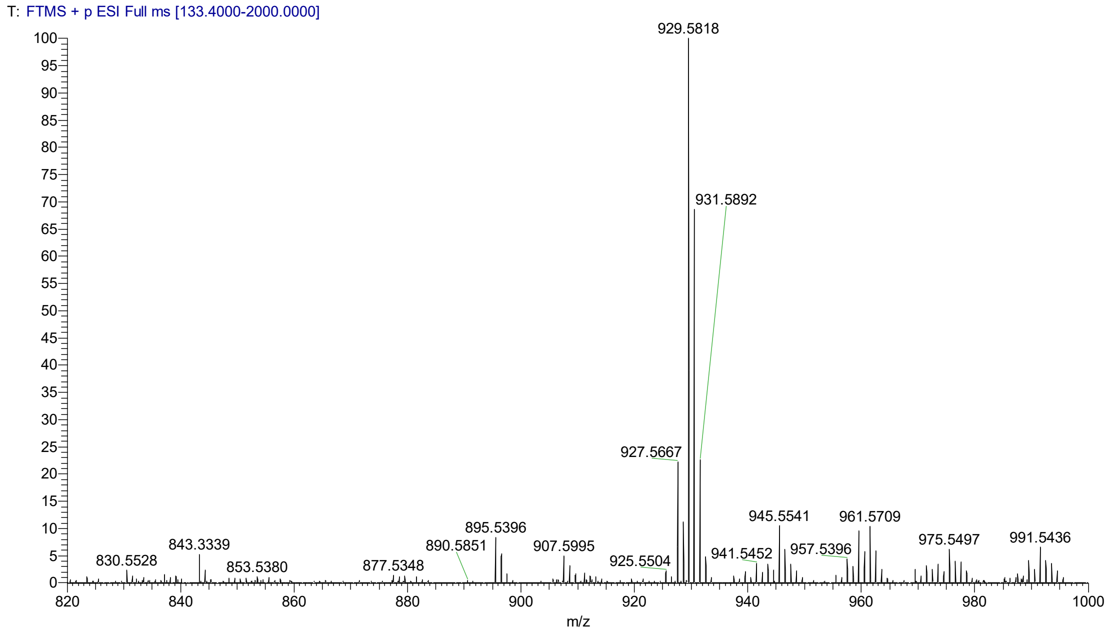

Figure S15. HRESIMS spectrum of compound **1**

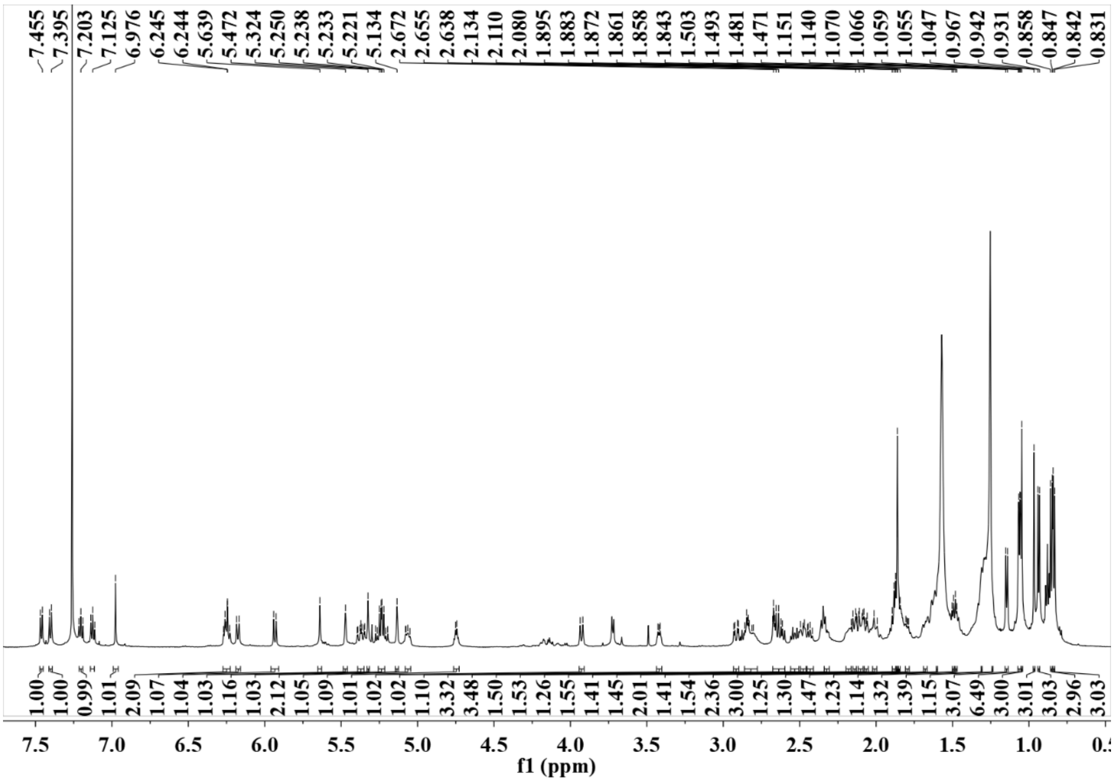

Figure S16. ^{1}H NMR (600 MHz, $CDCl_3$) spectrum of compound **2**

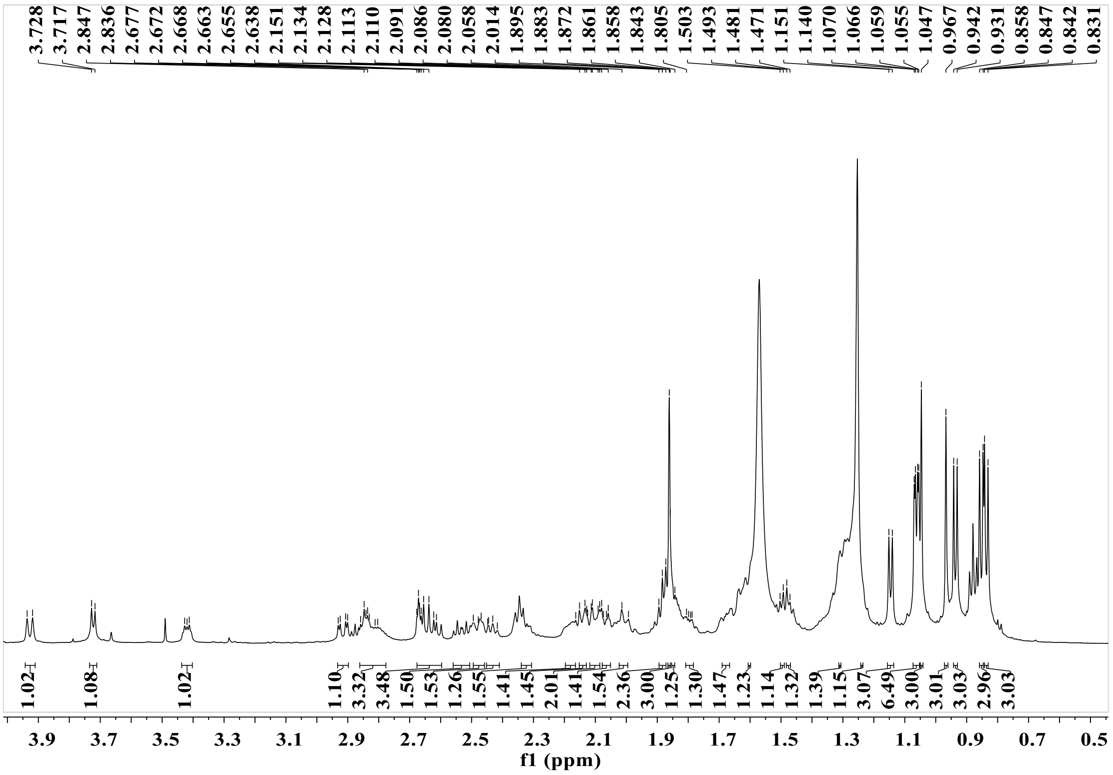

Figure S17. Partial enlarged ^{1}H NMR (600 MHz, $CDCl_3$) spectrum of compound **2**

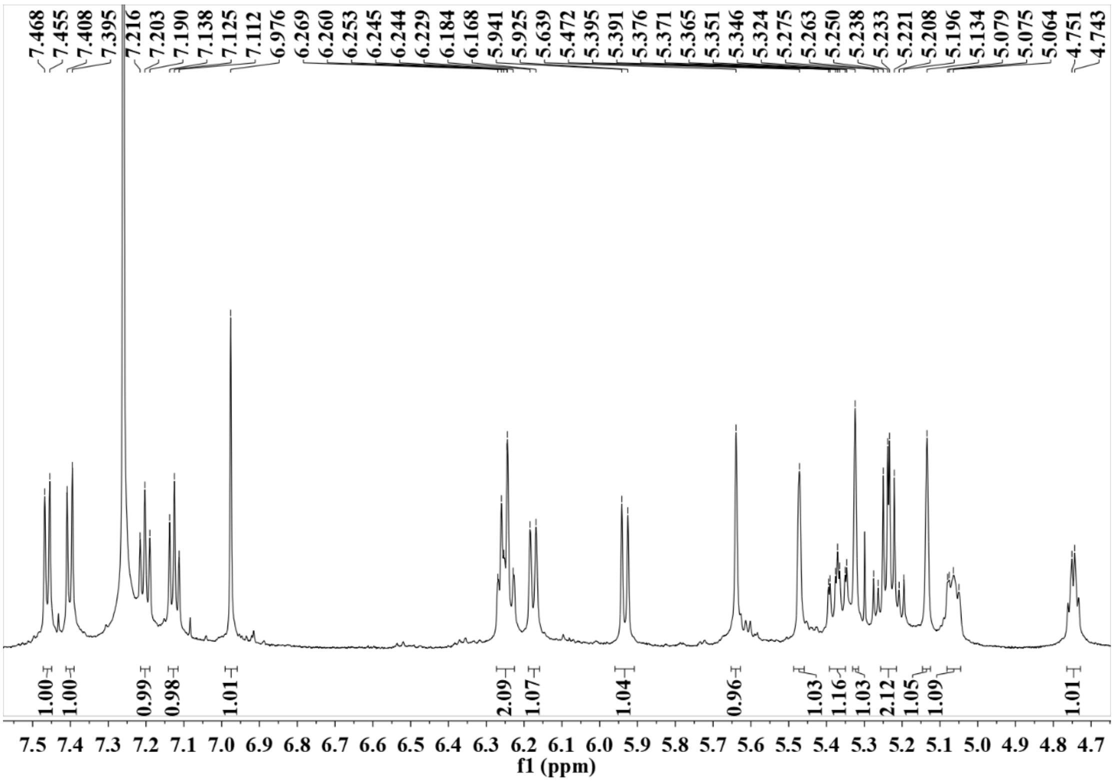

Figure S18. Partial enlarged ^{1}H NMR (600 MHz, $CDCl_3$) spectrum of compound **2**

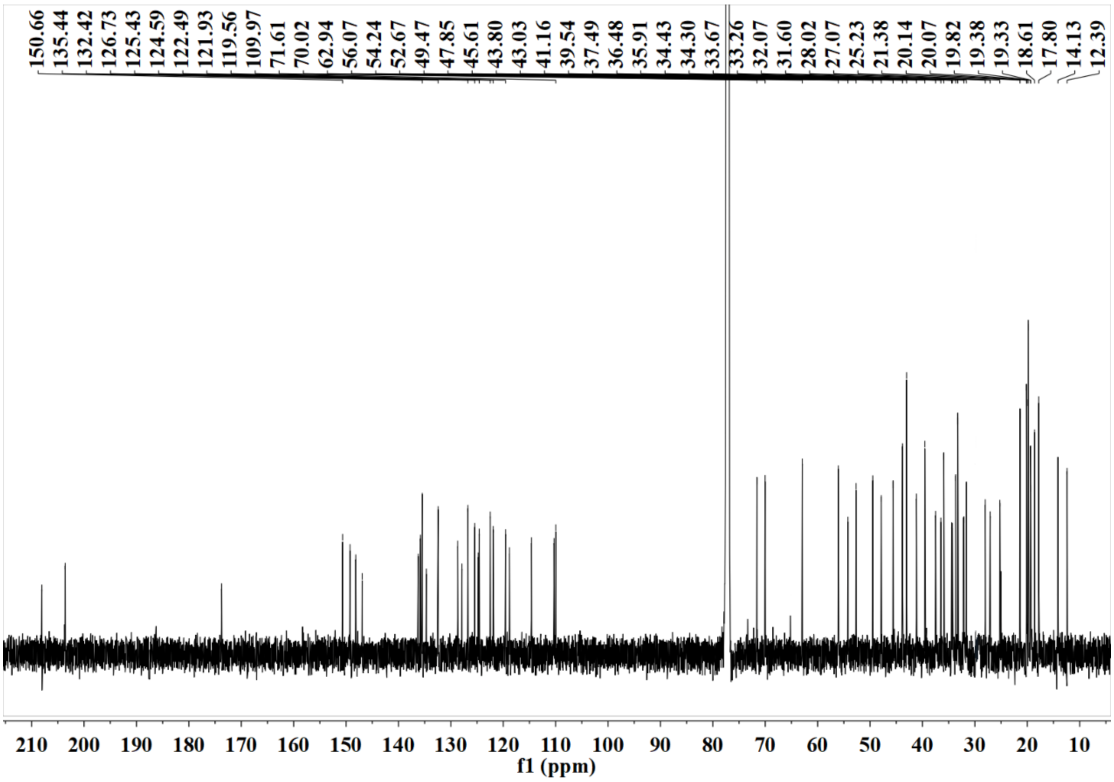

Figure S19. ^{13}C NMR (150 MHz, $CDCl_3$) spectrum of compound **2**

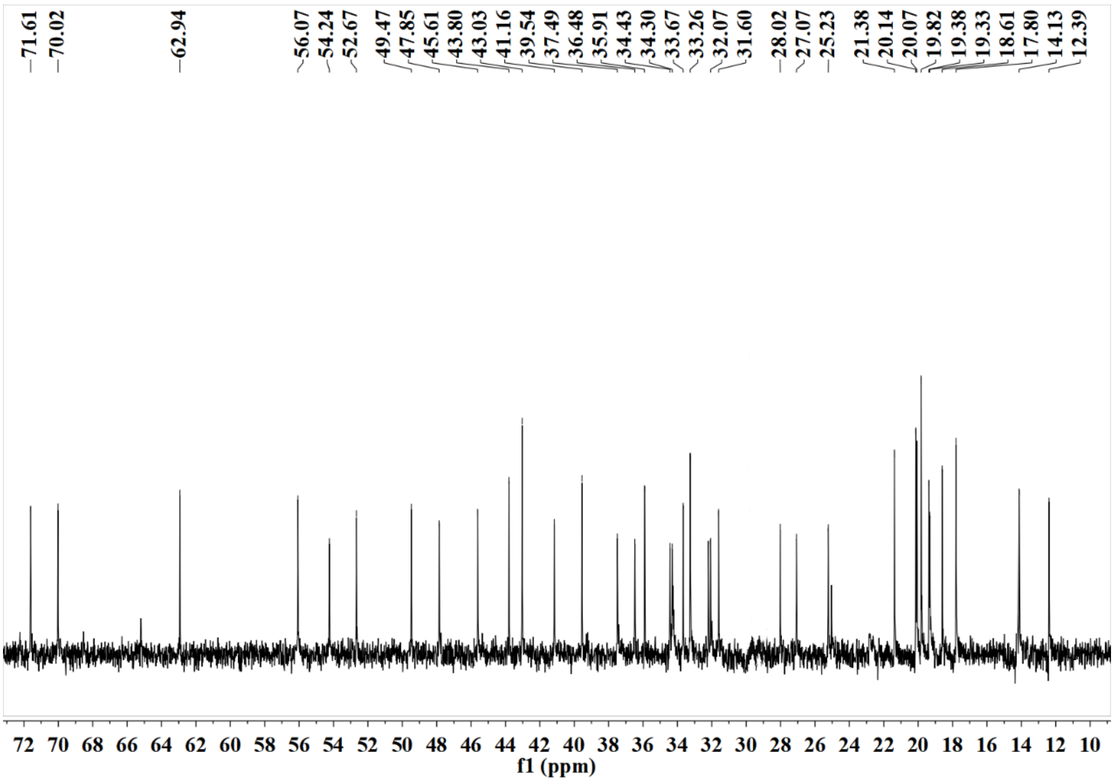

Figure S20. Partial enlarged ^{13}C NMR (150 MHz, $CDCl_3$) spectrum of compound **2**

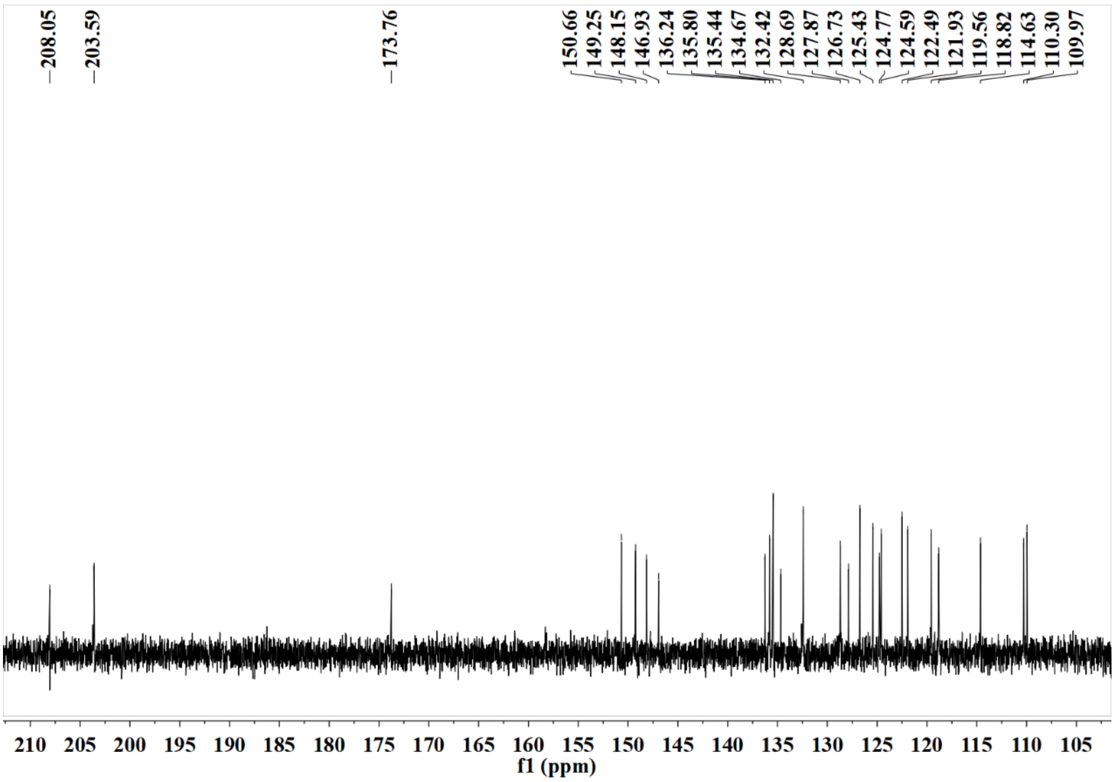

Figure S21. Partial enlarged ^{13}C NMR (150 MHz, $CDCl_3$) spectrum of compound **2**

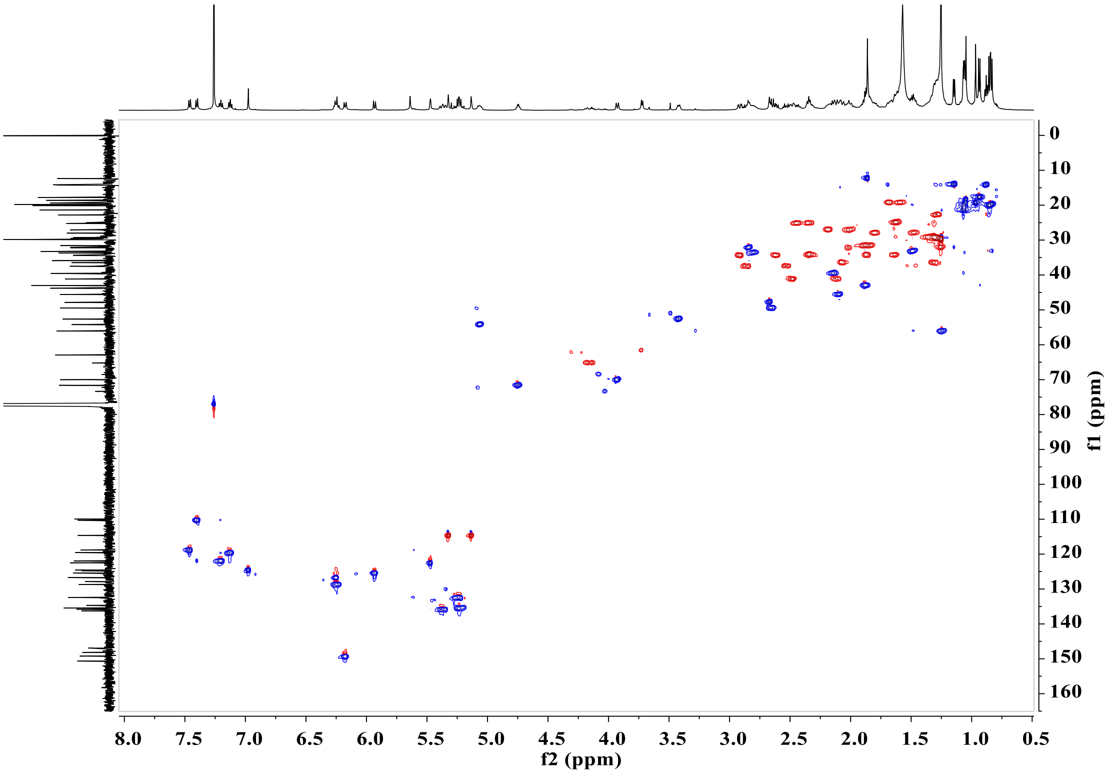

Figure S22. HSQC (600 MHz, $CDCl_3$) spectrum of compound **2**

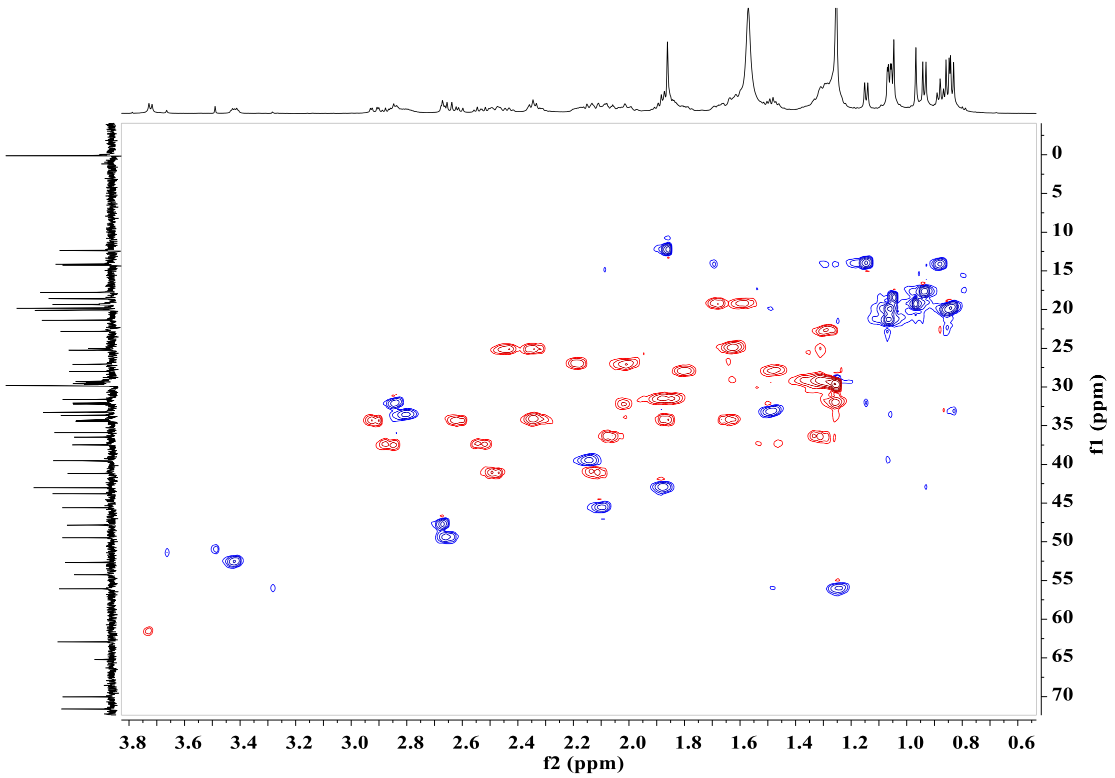

Figure S23. Partial enlarged HSQC (600 MHz, $CDCl_3$) spectrum of compound **2**

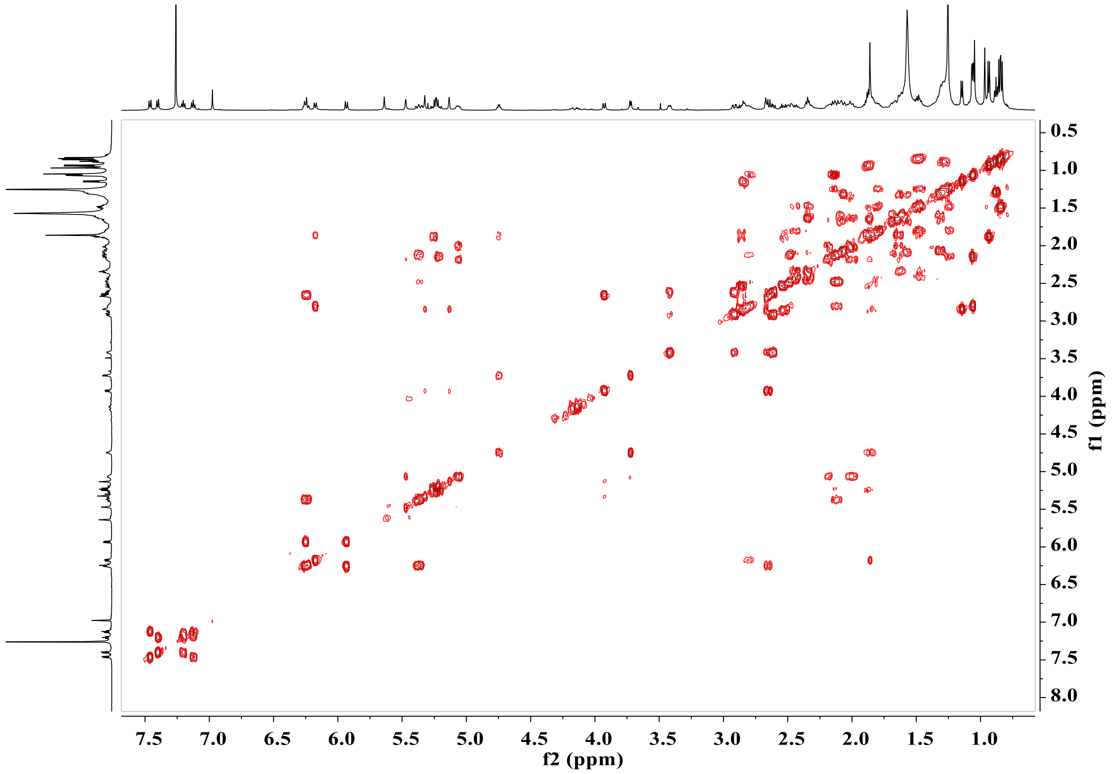

Figure S24. ^1H-^1H COSY (600 MHz, $CDCl_3$) spectrum of compound **2**

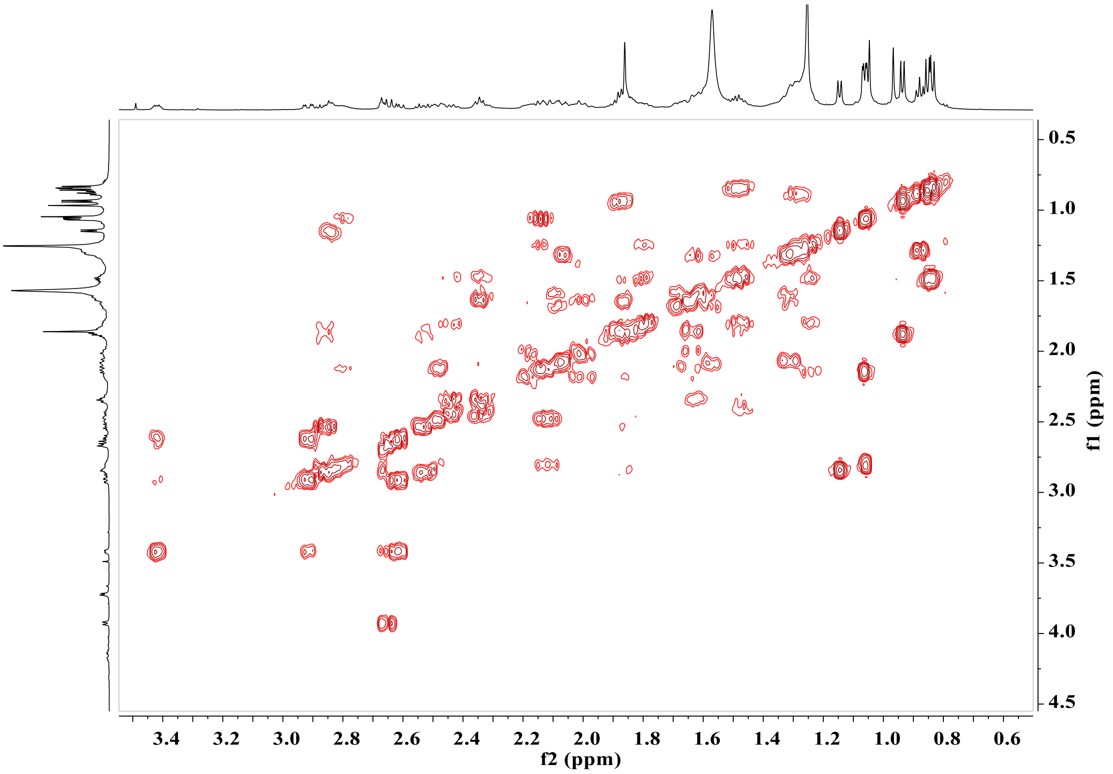

Figure S25. Partial enlarged ^{1}H-^{1}H COSY (600 MHz, $CDCl_3$) spectrum of compound **2**

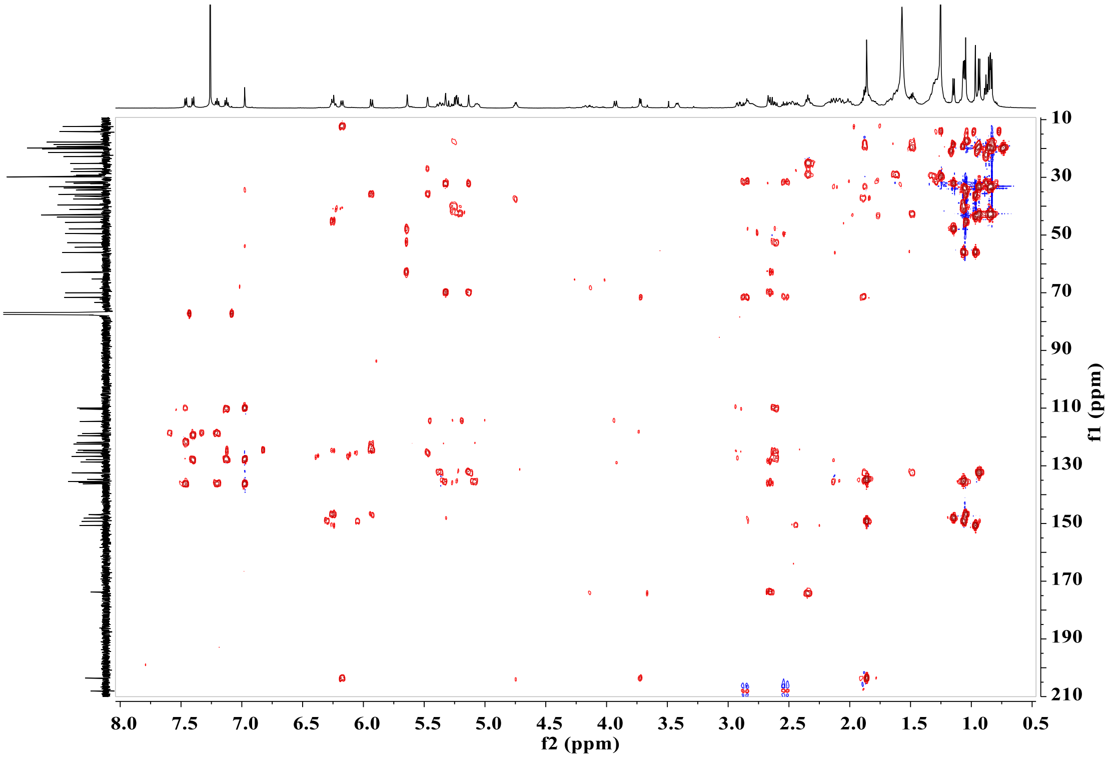

Figure S26. HMBC (600 MHz, $CDCl_3$) spectrum of compound **2**

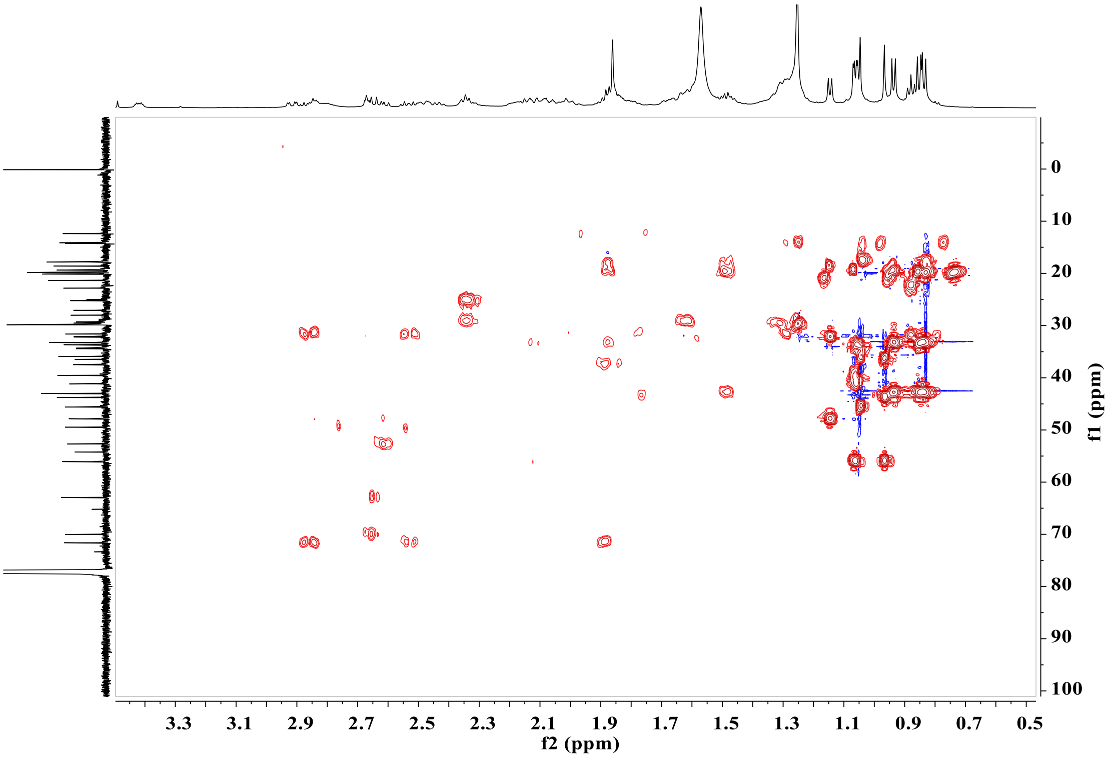

Figure S27. Partial enlarged HMBC (600 MHz, $CDCl_3$) spectrum of compound **2**

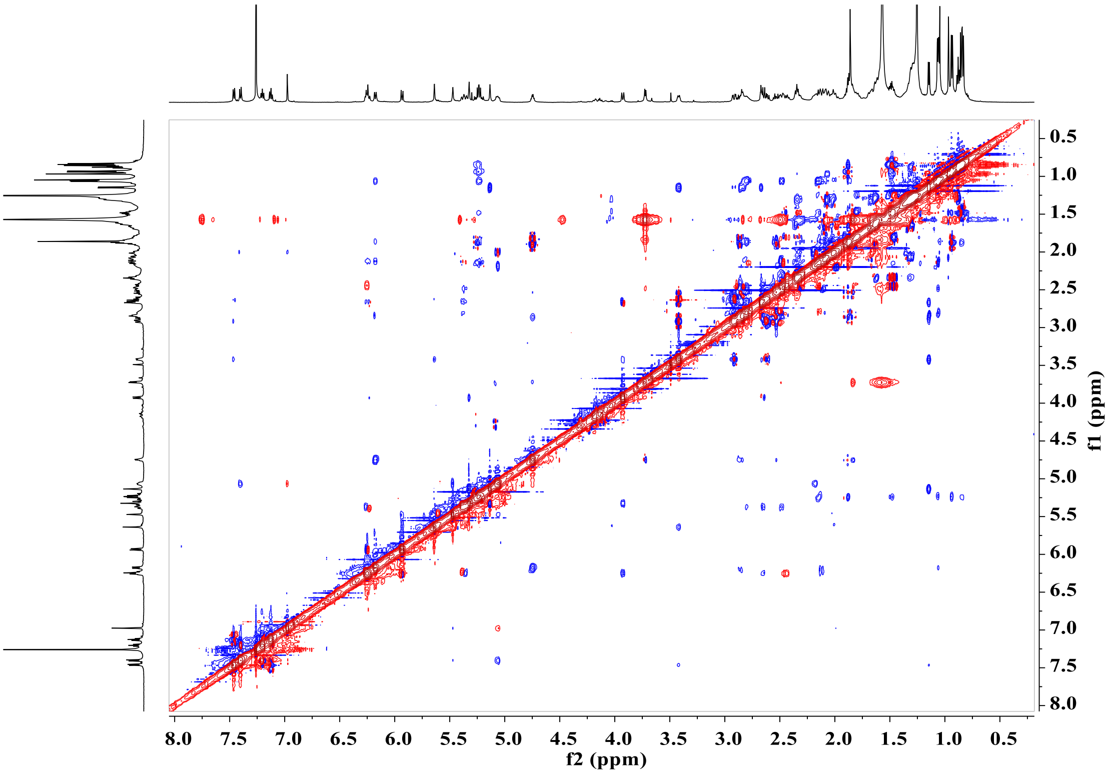

Figure S28. NOESY (600 MHz, $CDCl_3$) spectrum of compound **2**

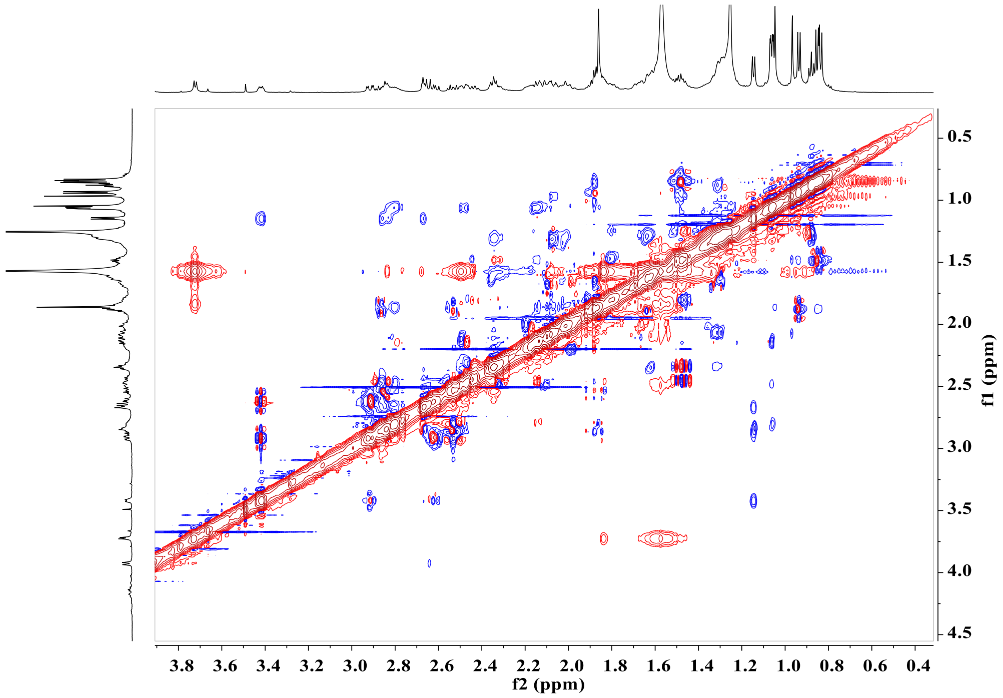

Figure S29. Partial enlarged NOESY (600 MHz, $CDCl_3$) spectrum of compound **2**

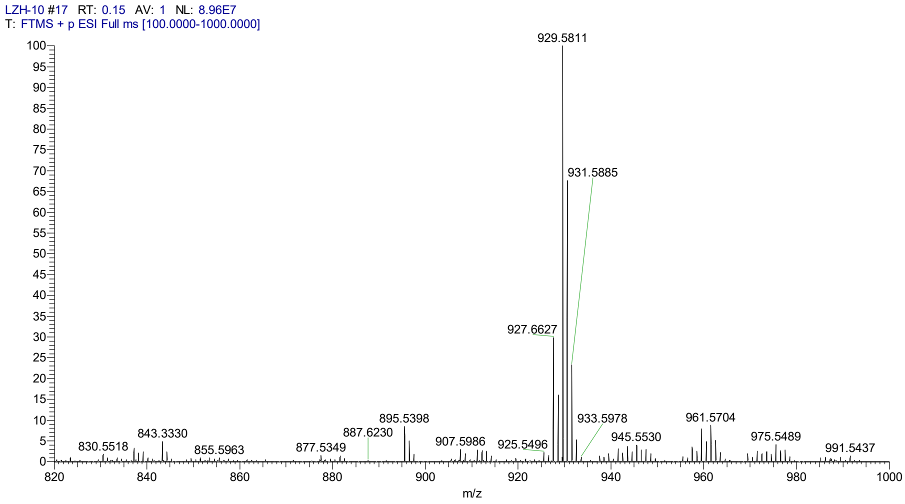

Figure S30. HRESIMS spectrum of compound **2**

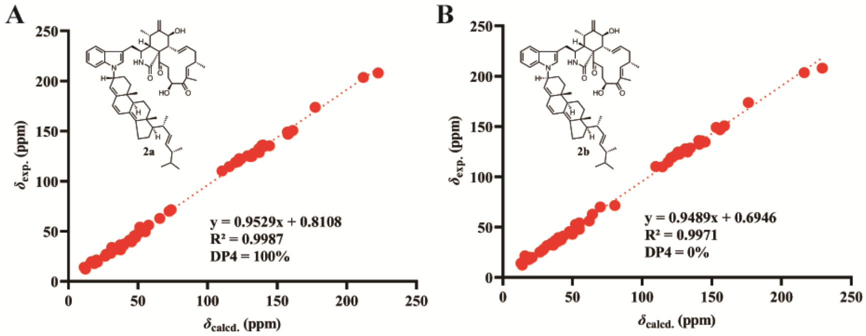

Figure S31. (A) Linear Regression analysis and DP4 analysis of the experimental and calculated ^{13}C NMR chemical shifts of **2a**. (B) Linear Regression analysis and DP4 analysis of the experimental and calculated ^{13}C NMR chemical shifts of **2b**.

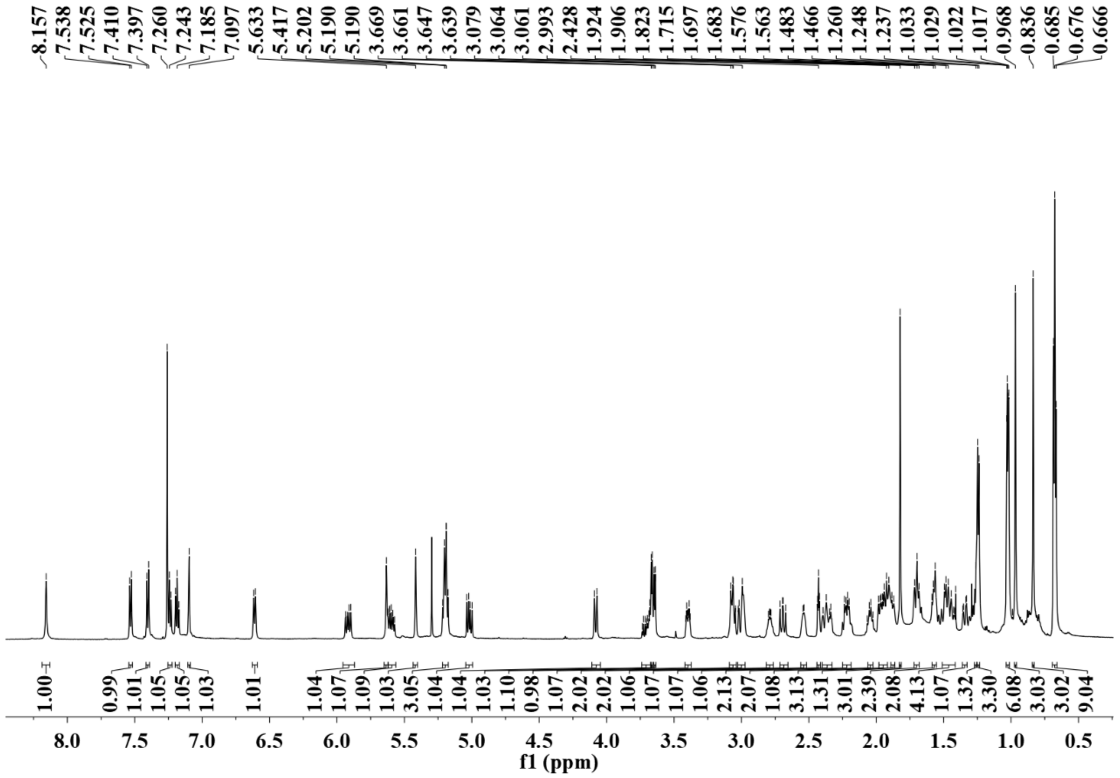

Figure S32. ^{1}H NMR (600 MHz, $CDCl_3$) spectrum of compound **3**

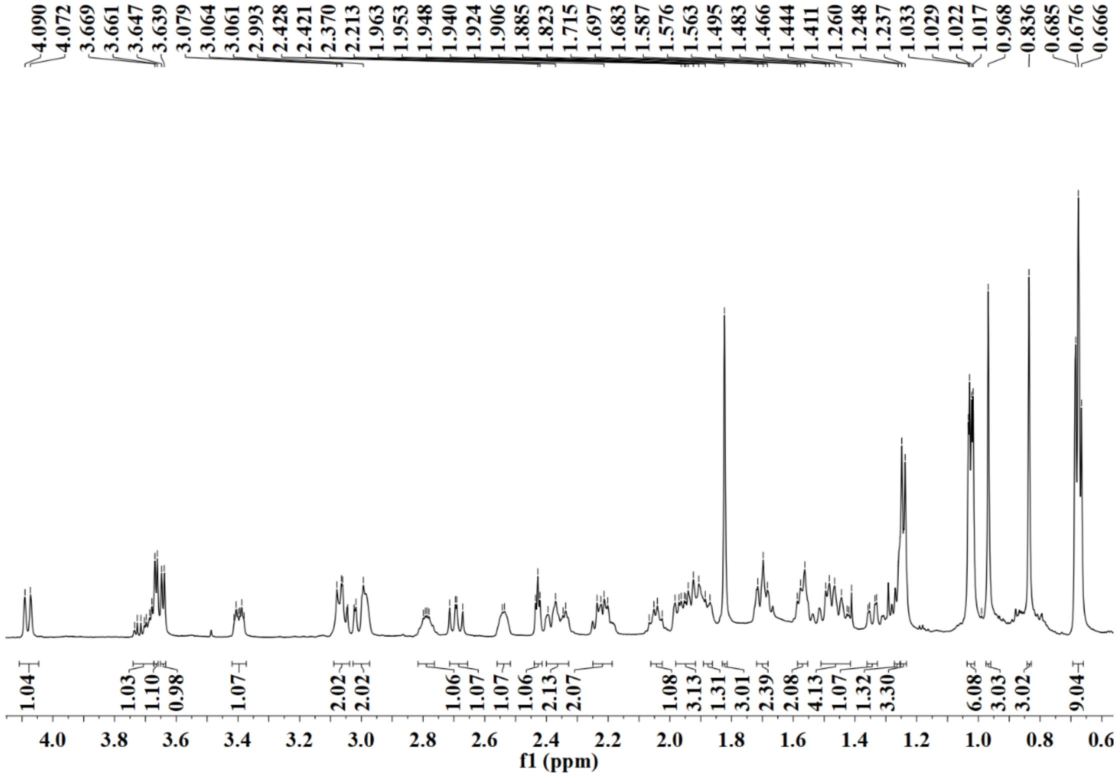

Figure S33. Partial enlarged ^{1}H NMR (600 MHz, $CDCl_3$) spectrum of compound **3**

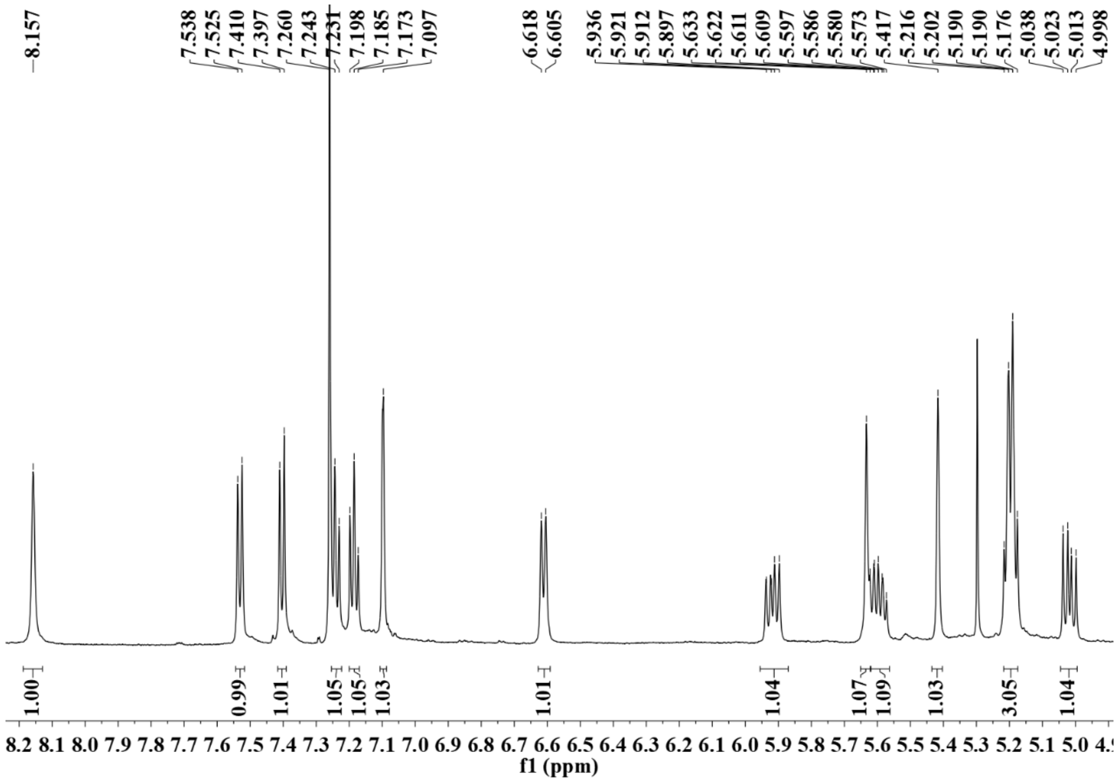

Figure S34. Partial enlarged ^{1}H NMR (600 MHz, $CDCl_3$) spectrum of compound **3**

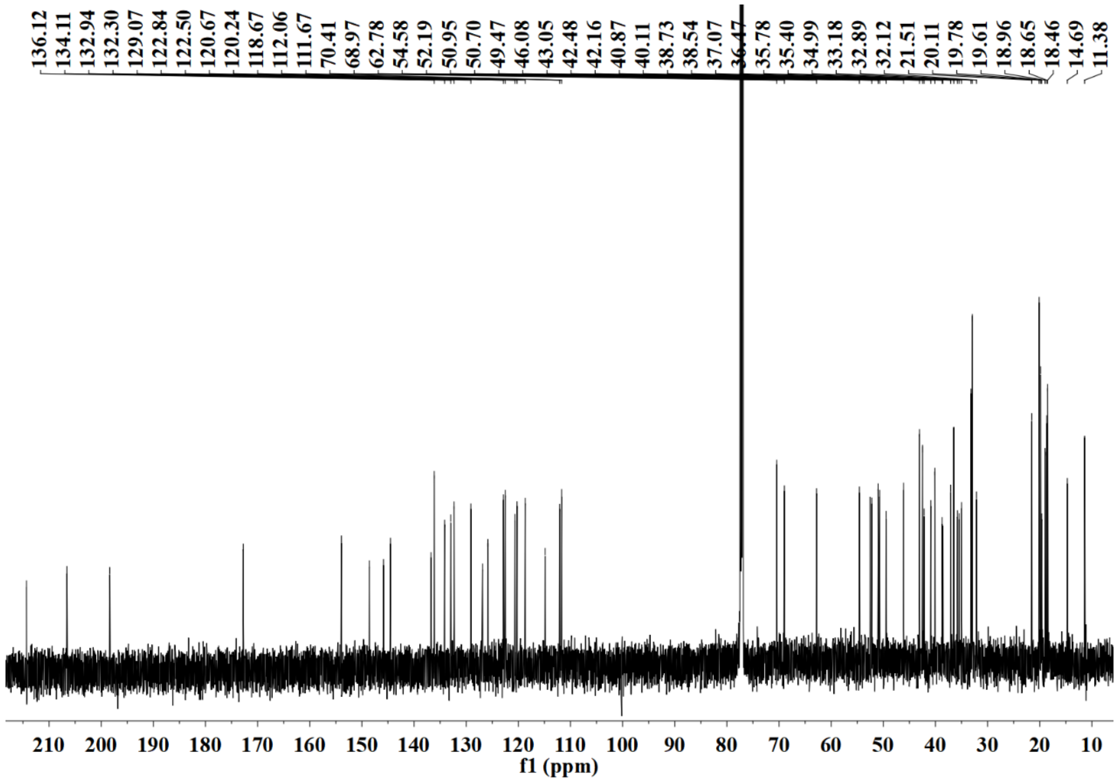

Figure S35. ^{13}C NMR (150 MHz, $CDCl_3$) spectrum of compound **3**

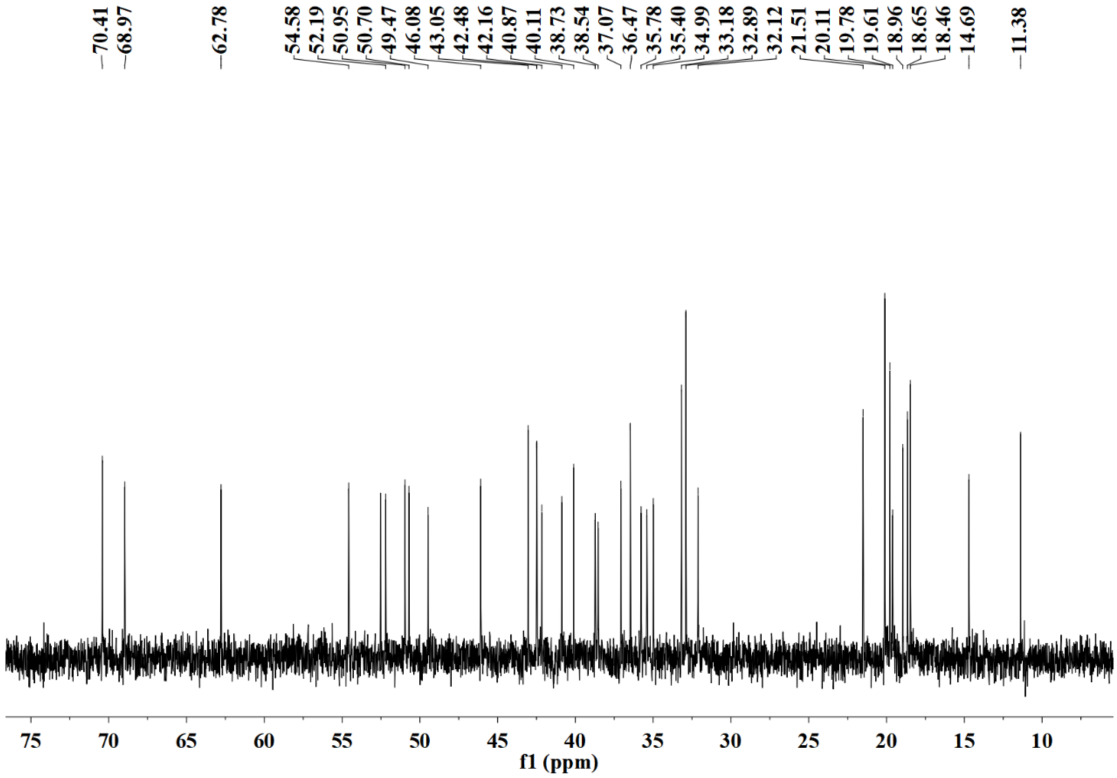

Figure S36. Partial enlarged ^{13}C NMR (150 MHz, $CDCl_3$) spectrum of compound **3**

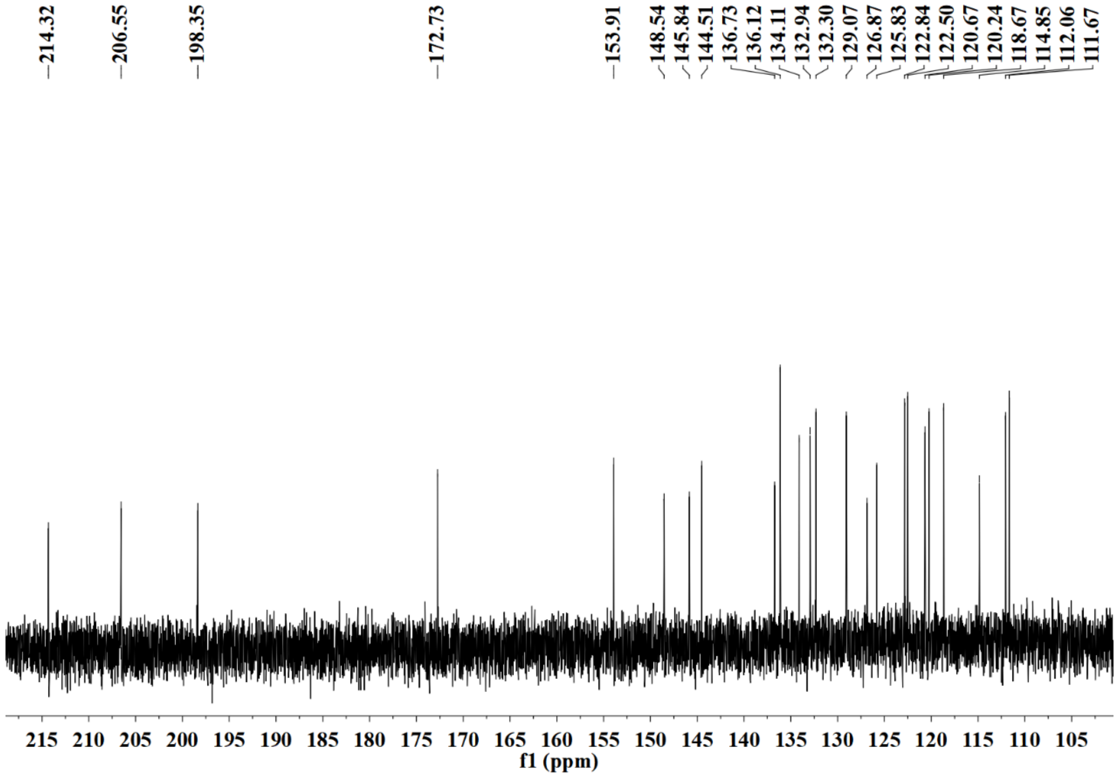

Figure S37. Partial enlarged ^{13}C NMR (150 MHz, $CDCl_3$) spectrum of compound **3**

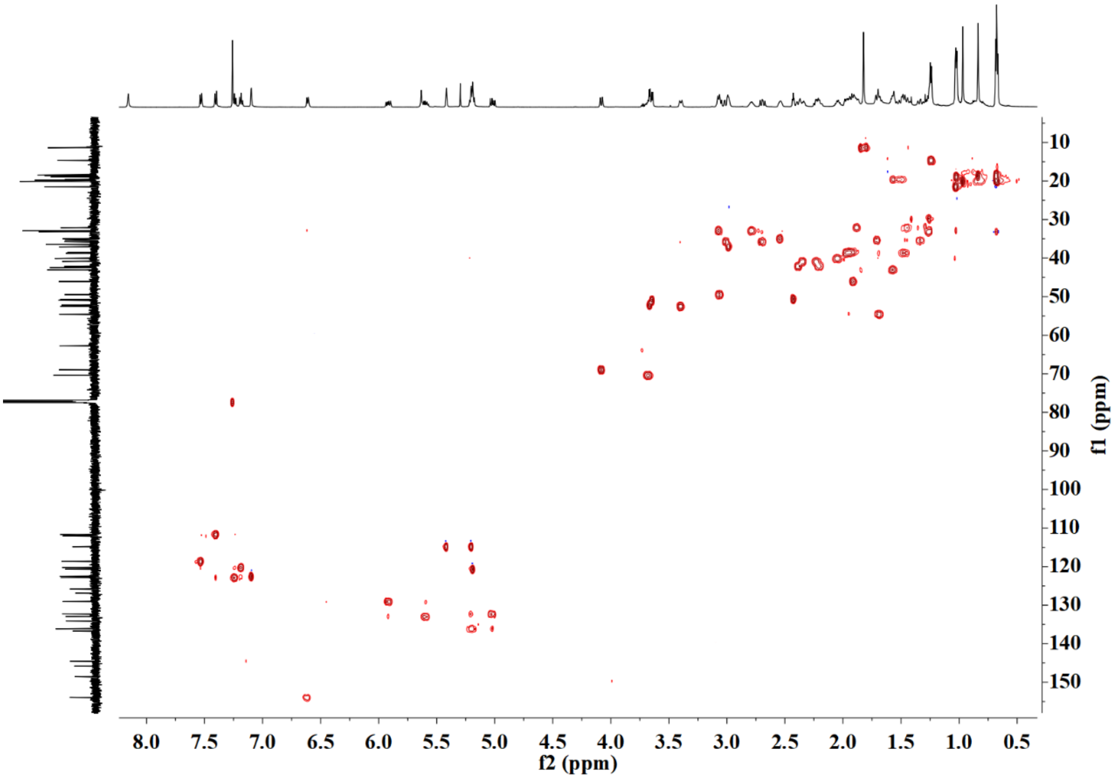

Figure S38. HSQC (600 MHz, $CDCl_3$) spectrum of compound **3**

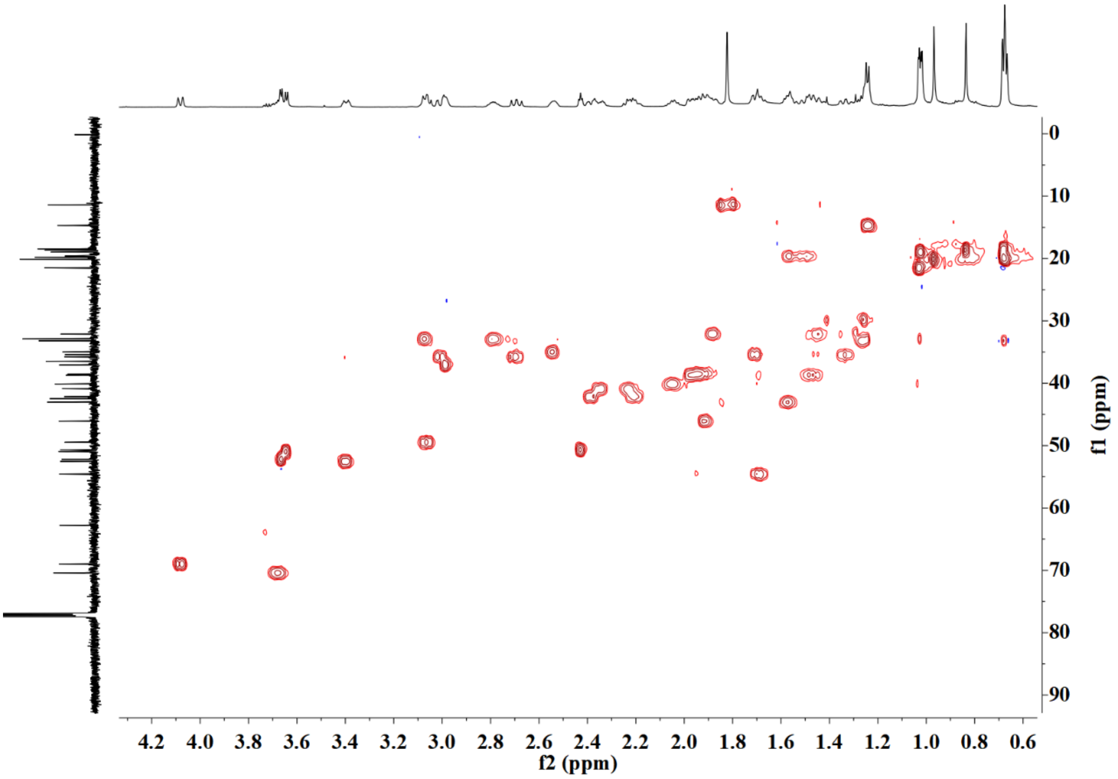

Figure S39. Partial enlarged HSQC (600 MHz, $CDCl_3$) spectrum of compound **3**

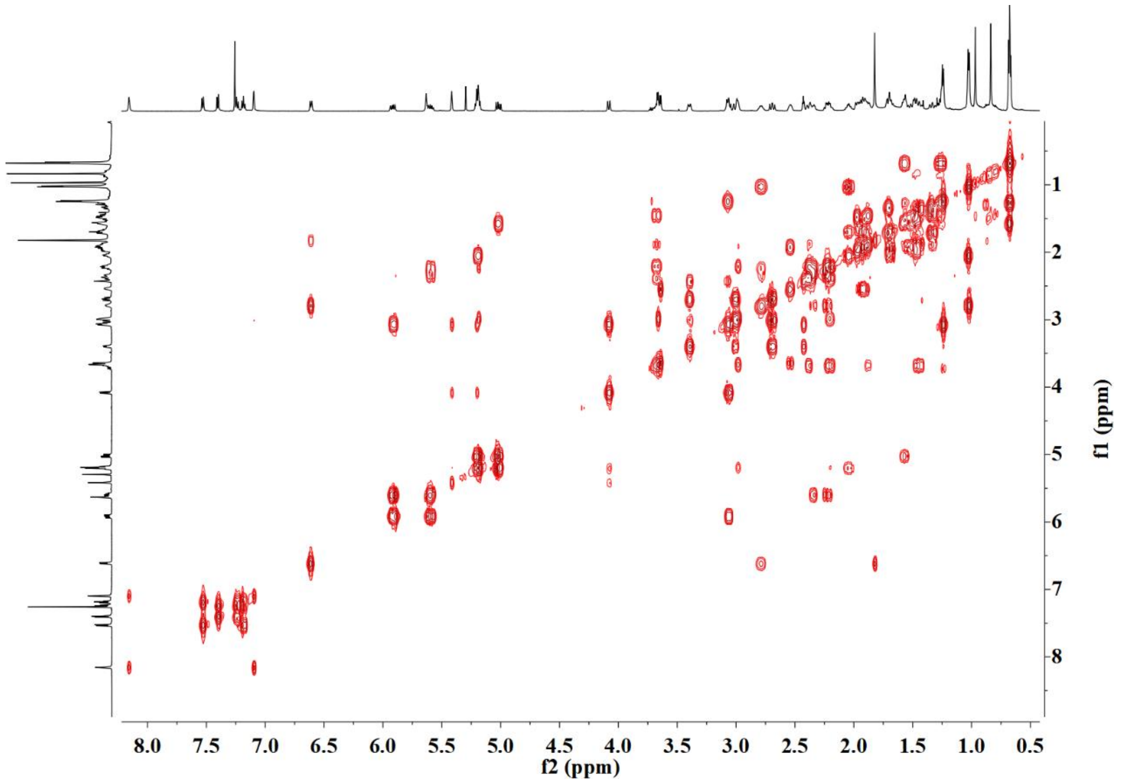

Figure S40. ^1H-^1H COSY (600 MHz, $CDCl_3$) spectrum of compound **3**

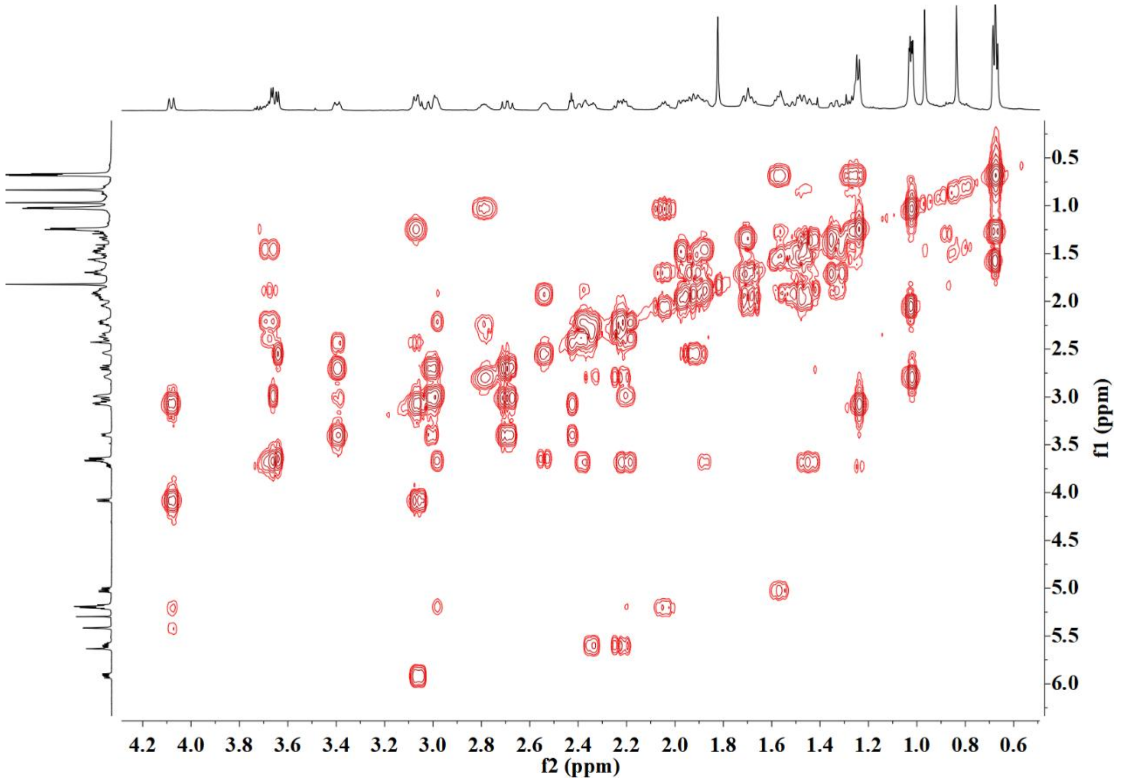

Figure S41. Partial enlarged ^1H-^1H COSY (600 MHz, $CDCl_3$) spectrum of compound **3**

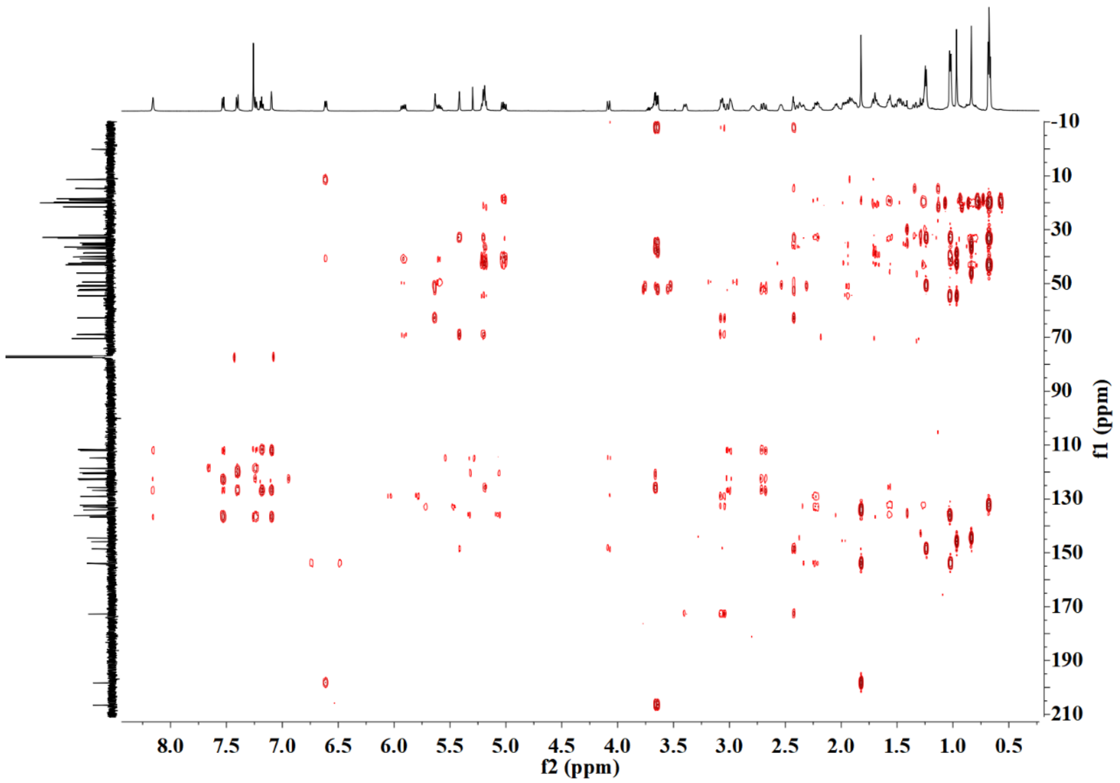

Figure S42. HMBC (600 MHz, $CDCl_3$) spectrum of compound **3**

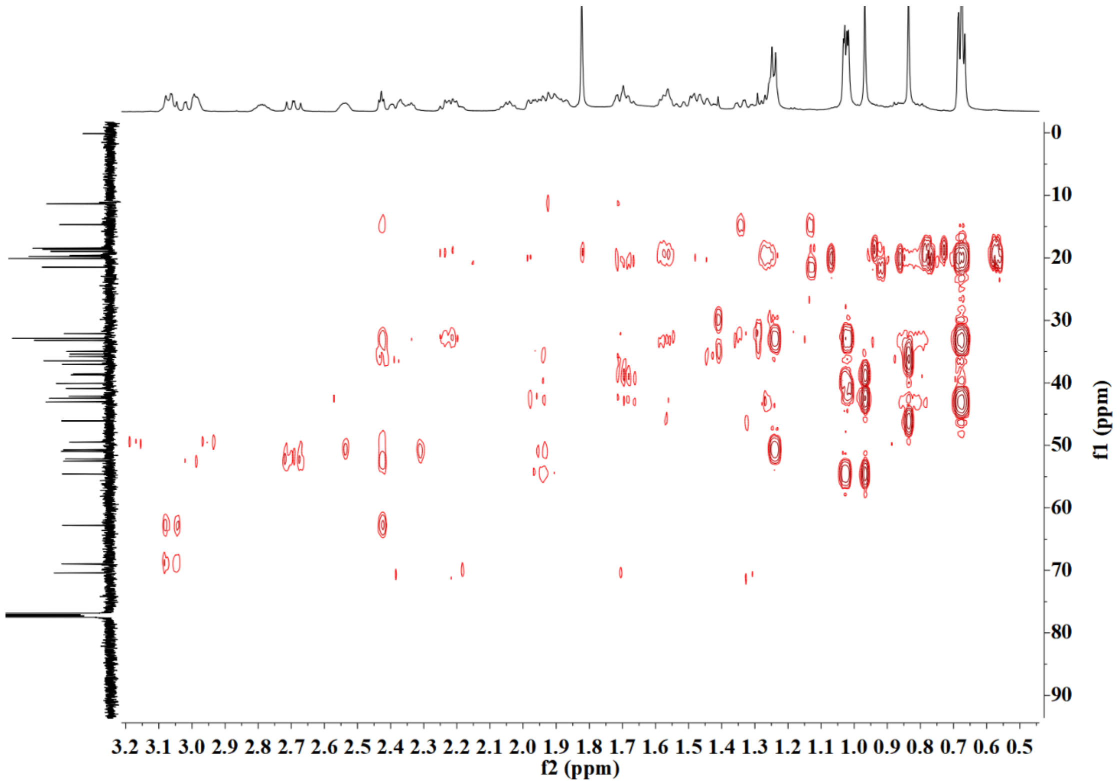

Figure S43. Partial enlarged HMBC (600 MHz, $CDCl_3$) spectrum of compound **3**

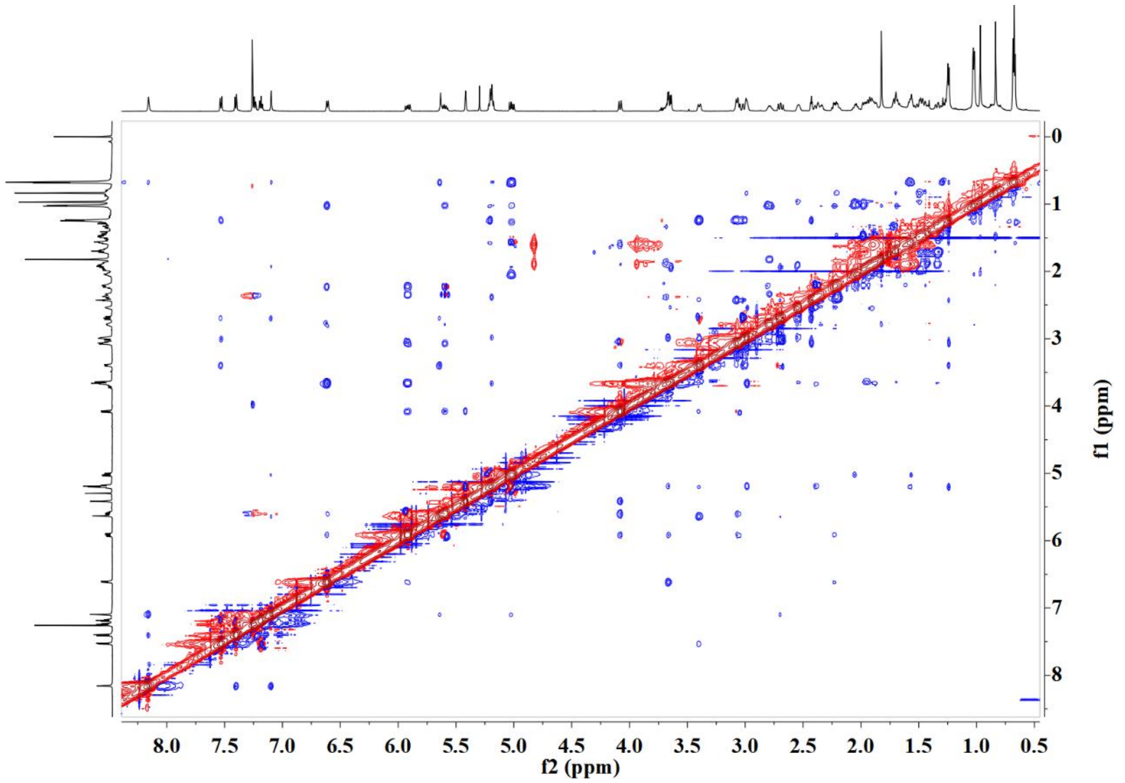

Figure S44. NOESY (600 MHz, $CDCl_3$) spectrum of compound **3**

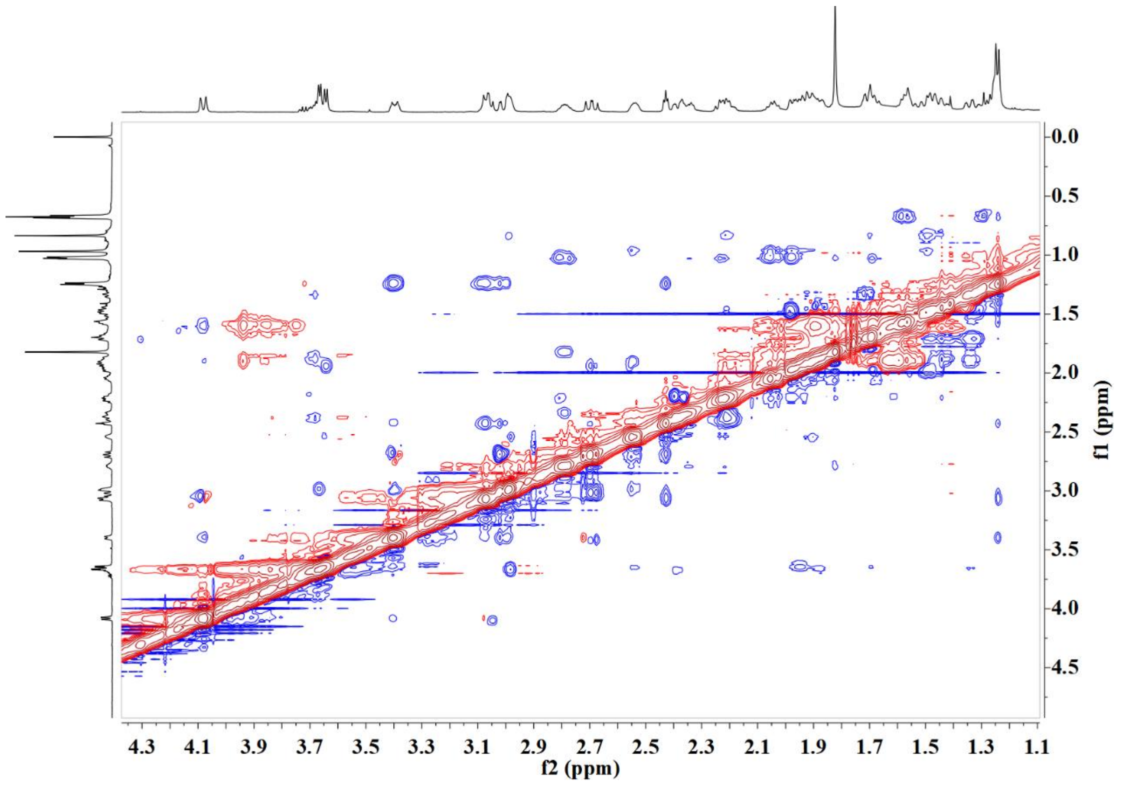

Figure S45. Partial enlarged NOESY (600 MHz, $CDCl_3$) spectrum of compound **3**

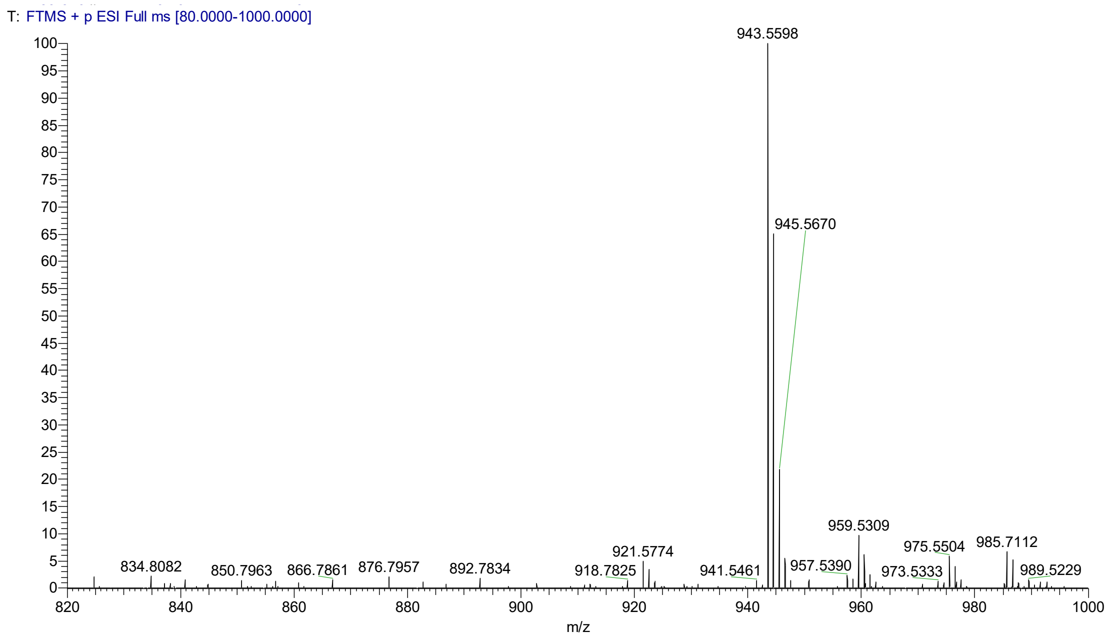

Figure S46. HRESIMS spectrum of compound **3**

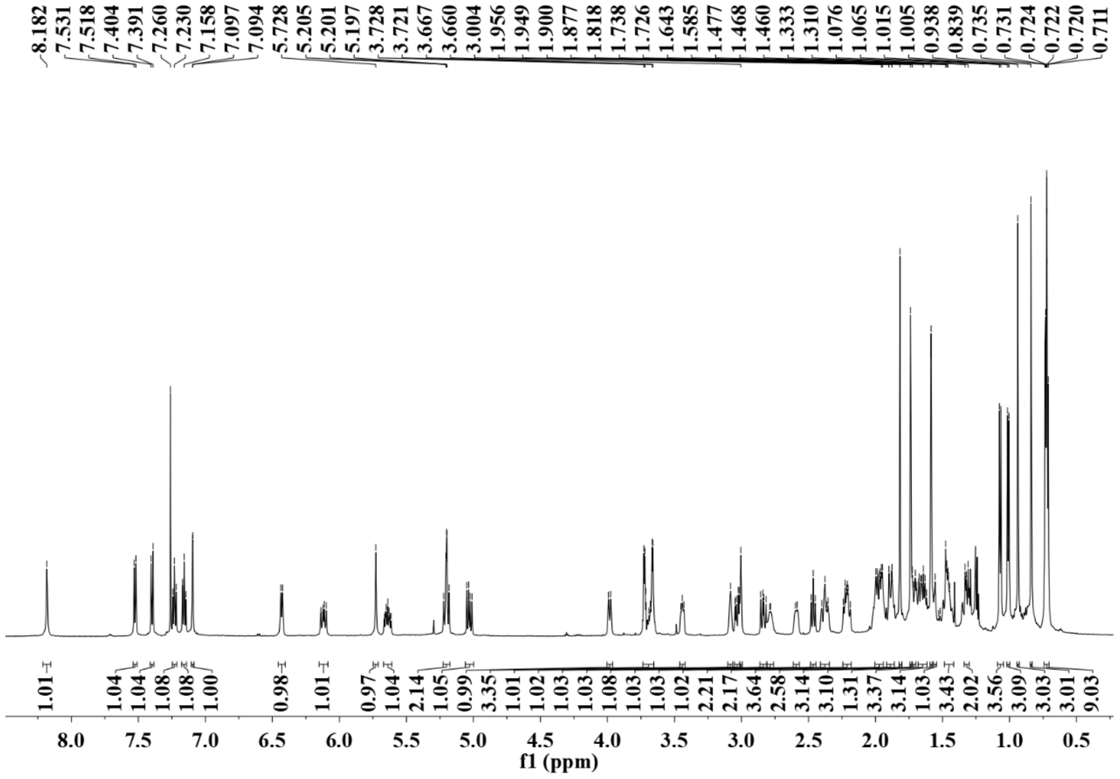

Figure S47. ^{1}H NMR (600 MHz, $CDCl_3$) spectrum of compound **4**

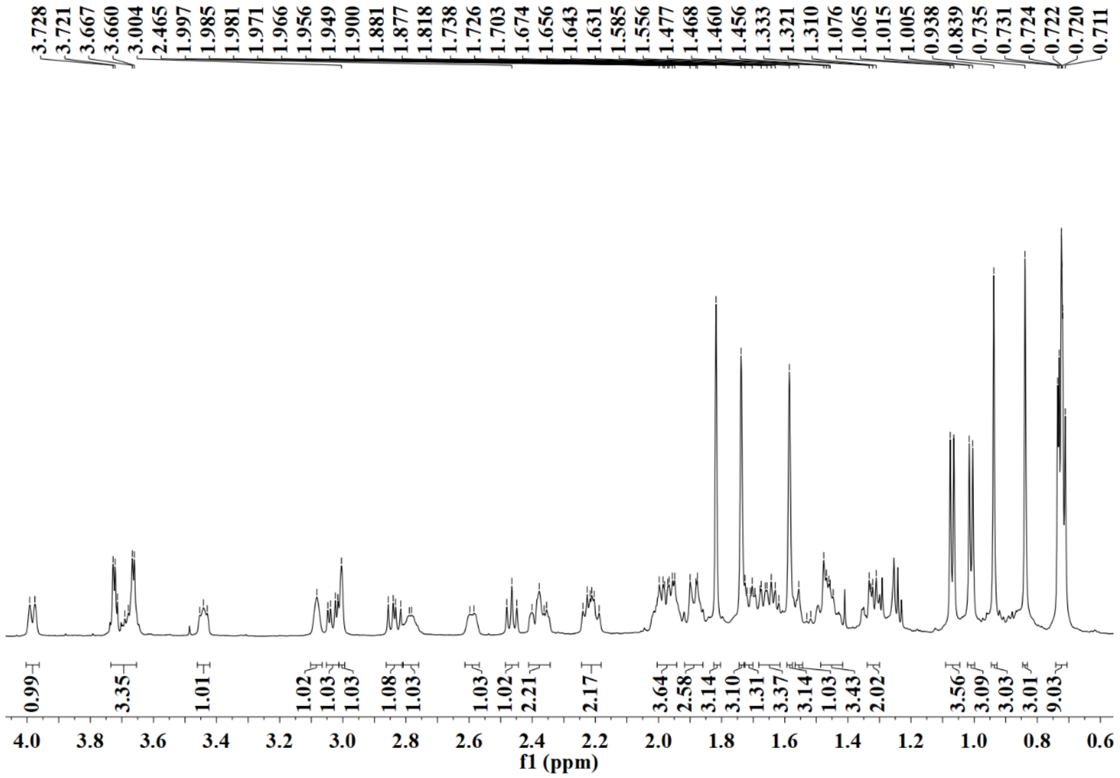

Figure S48. Partial enlarged ^{1}H NMR (600 MHz, $CDCl_3$) spectrum of compound **4**

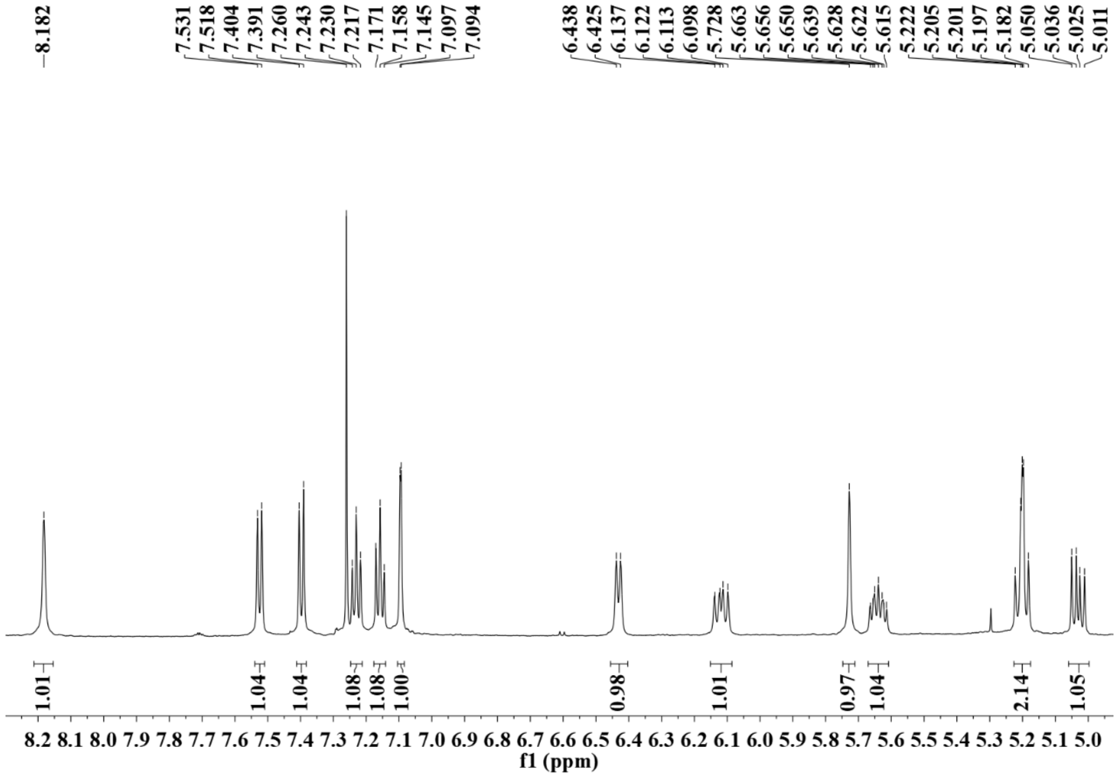

Figure S49. Partial enlarged ^{1}H NMR (600 MHz, $CDCl_3$) spectrum of compound **4**

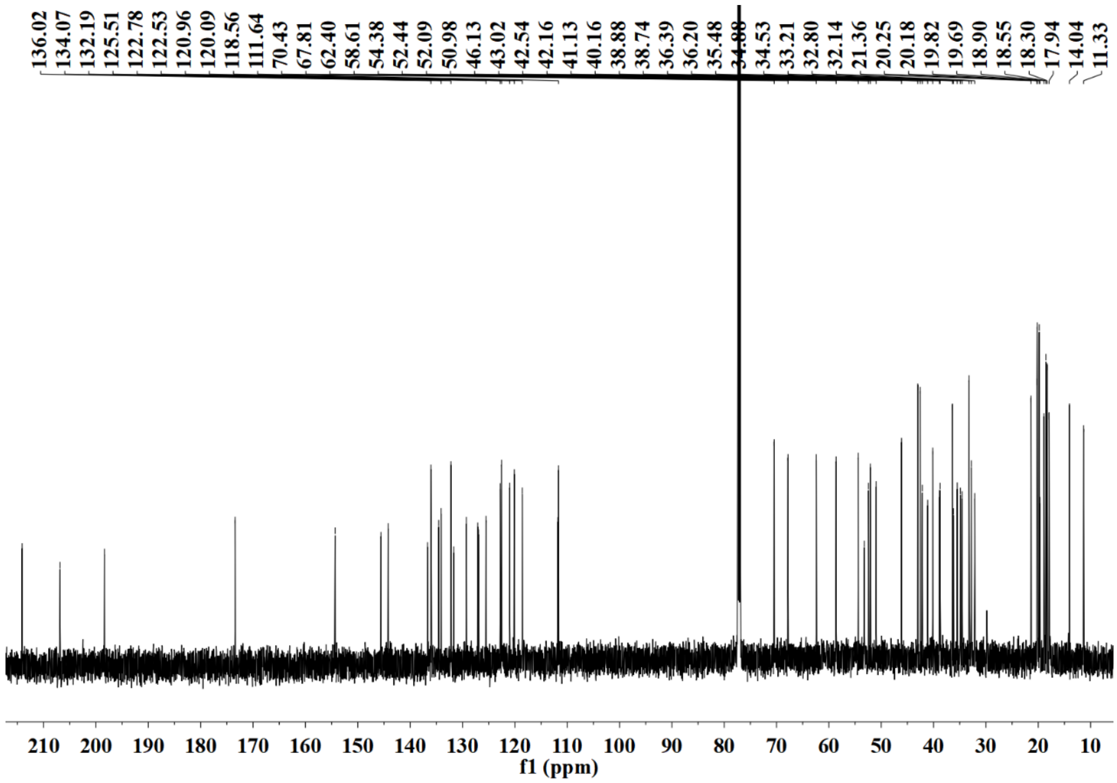

Figure S50. ^{13}C NMR (150 MHz, $CDCl_3$) spectrum of compound **4**

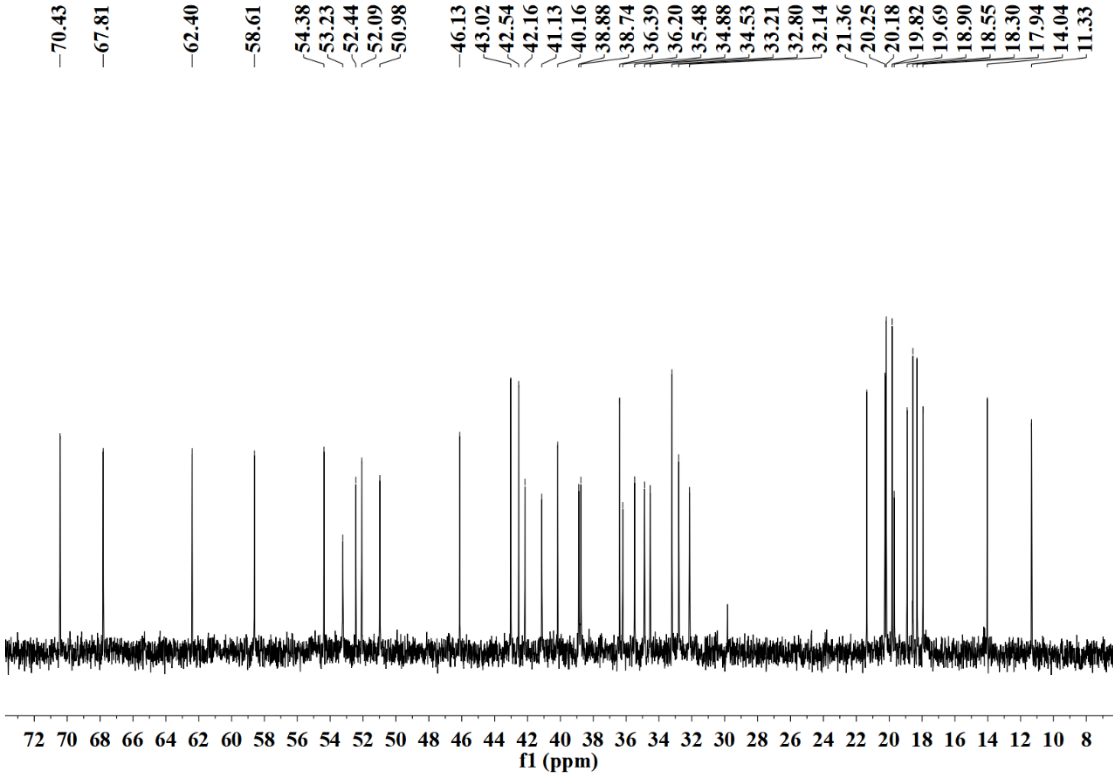

Figure S51. Partial enlarged ^{13}C NMR (150 MHz, $CDCl_3$) spectrum of compound **4**

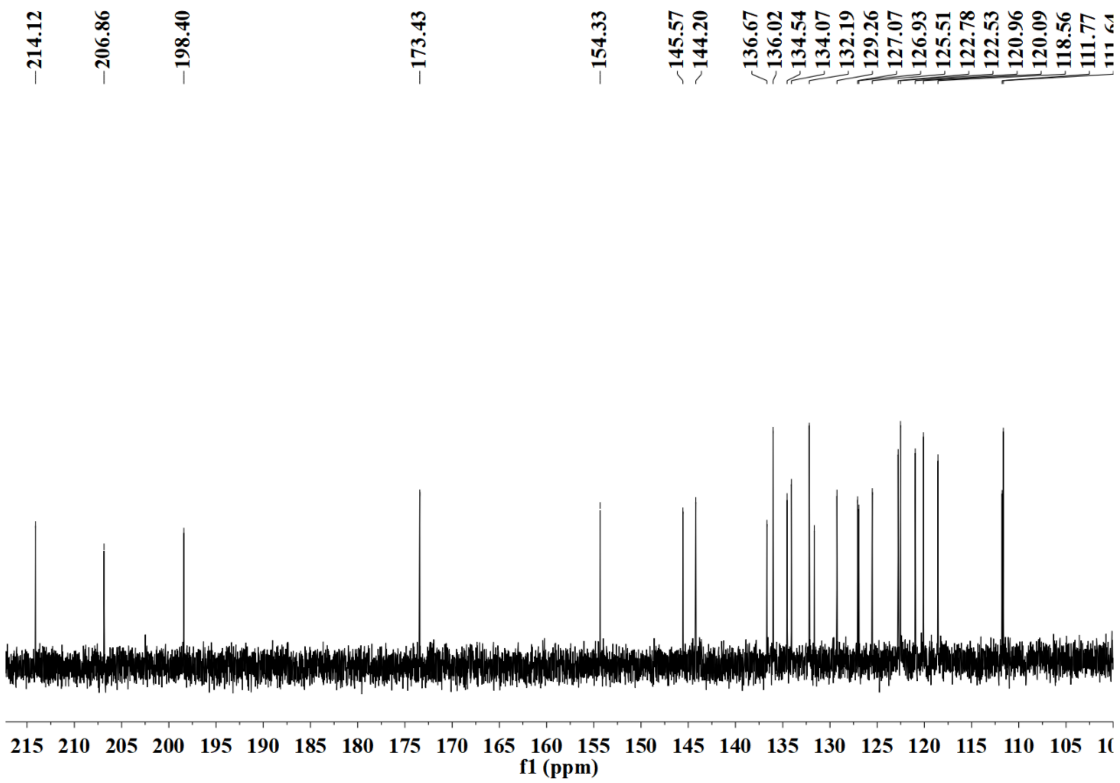

Figure S52. Partial enlarged ^{13}C NMR (150 MHz, $CDCl_3$) spectrum of compound **4**

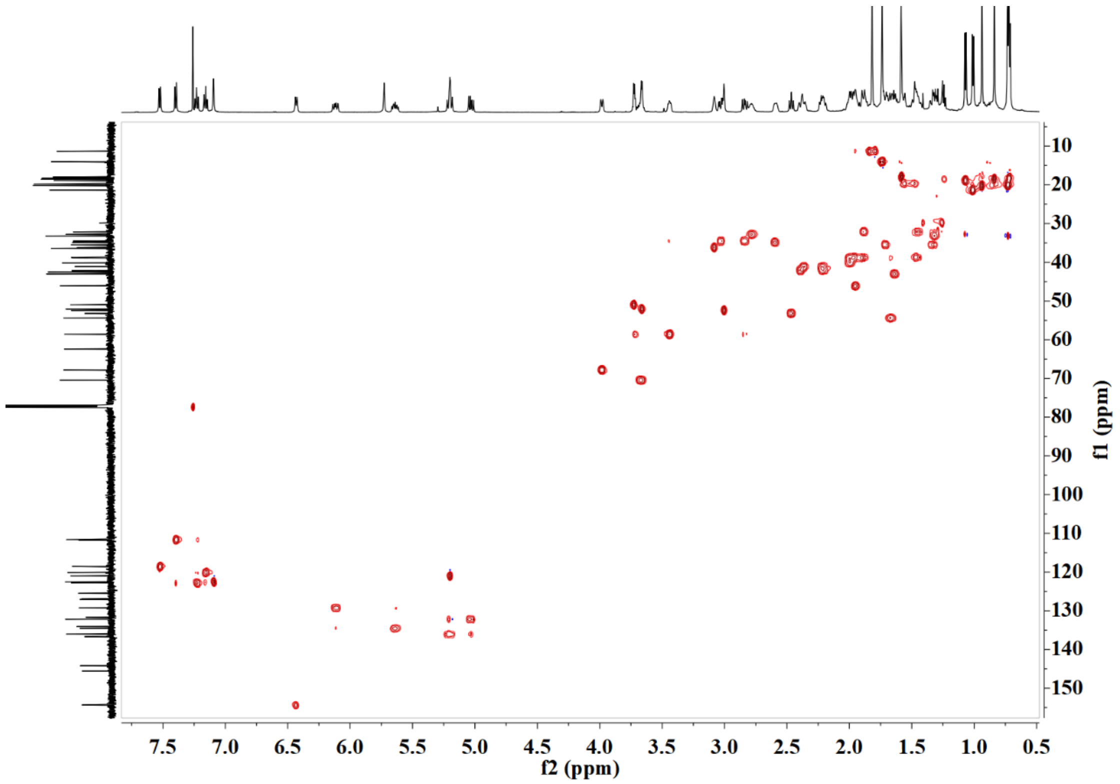

Figure S53. HSQC (600 MHz, $CDCl_3$) spectrum of compound **4**

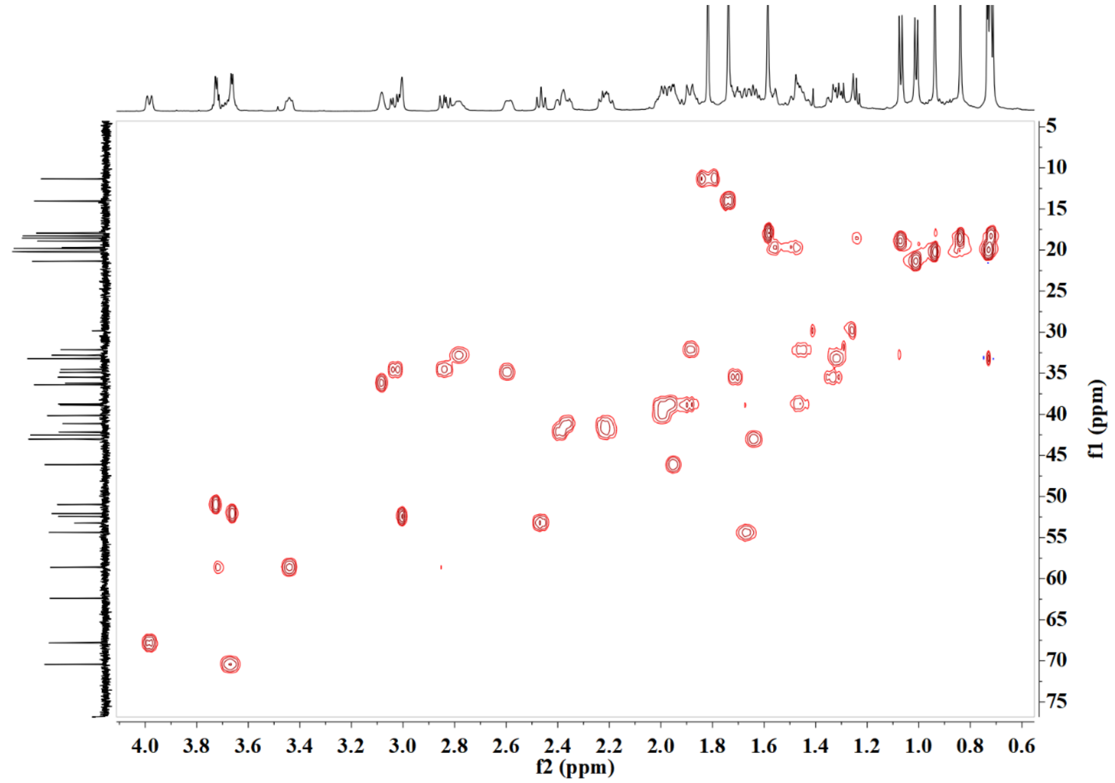

Figure S54. Partial enlarged HSQC (600 MHz, $CDCl_3$) spectrum of compound **4**

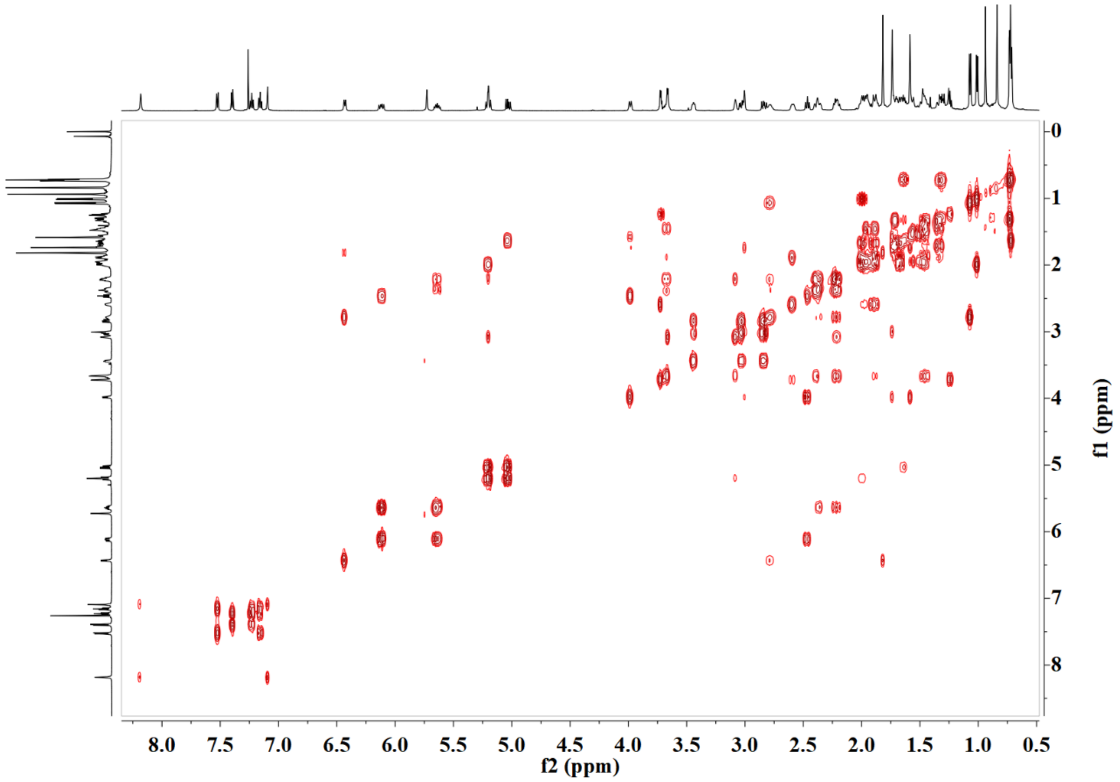

Figure S55. ^1H-^1H COSY (600 MHz, $CDCl_3$) spectrum of compound **4**

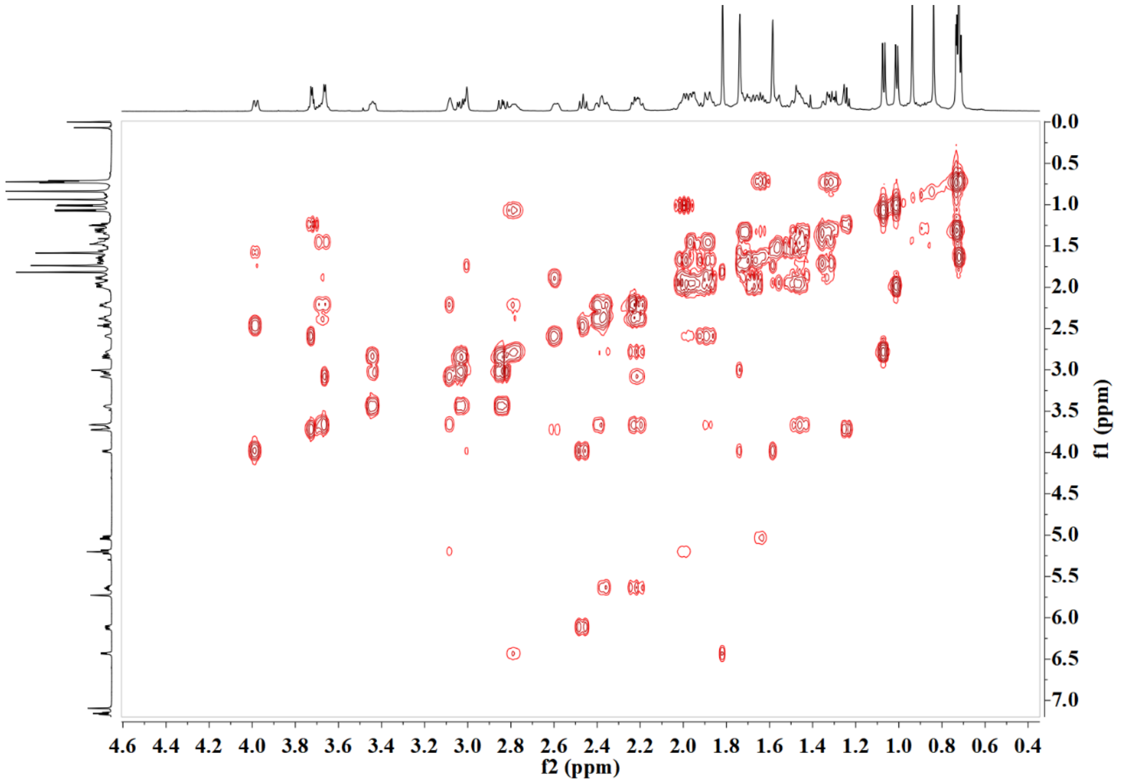

Figure S56. Partial enlarged ^{1}H-^{1}H COSY (600 MHz, $CDCl_3$) spectrum of compound **4**

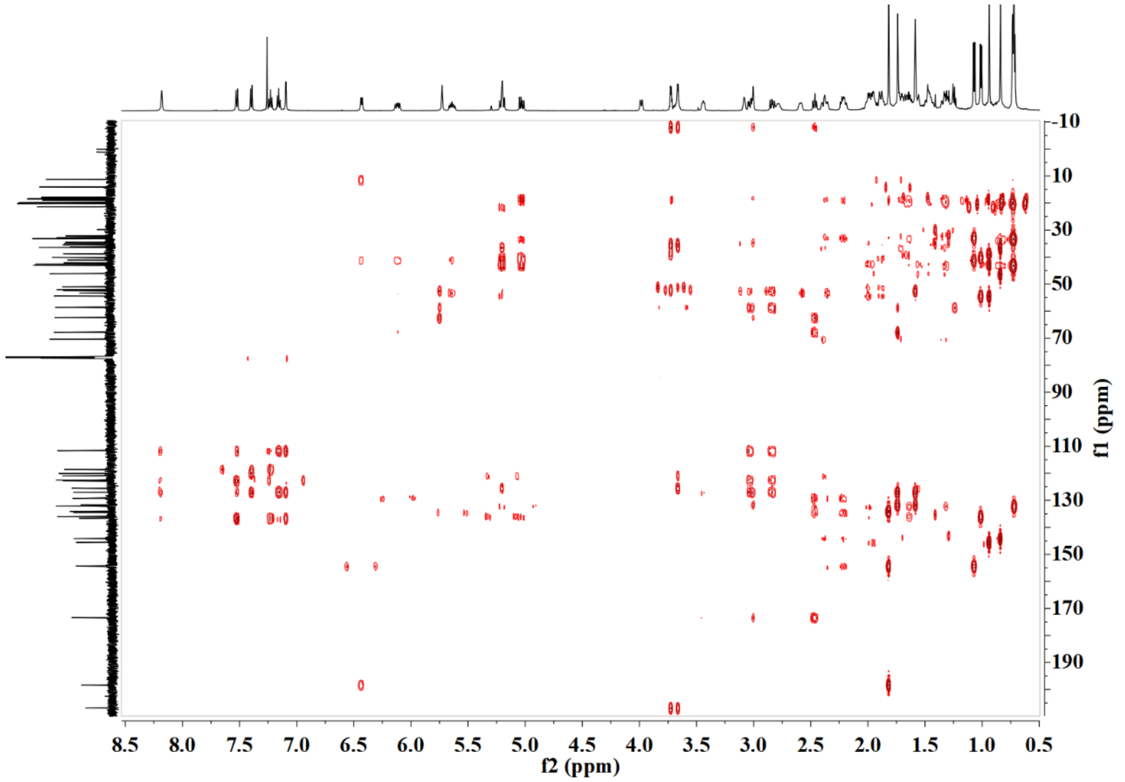

Figure S57. HMBC (600 MHz, $CDCl_3$) spectrum of compound **4**

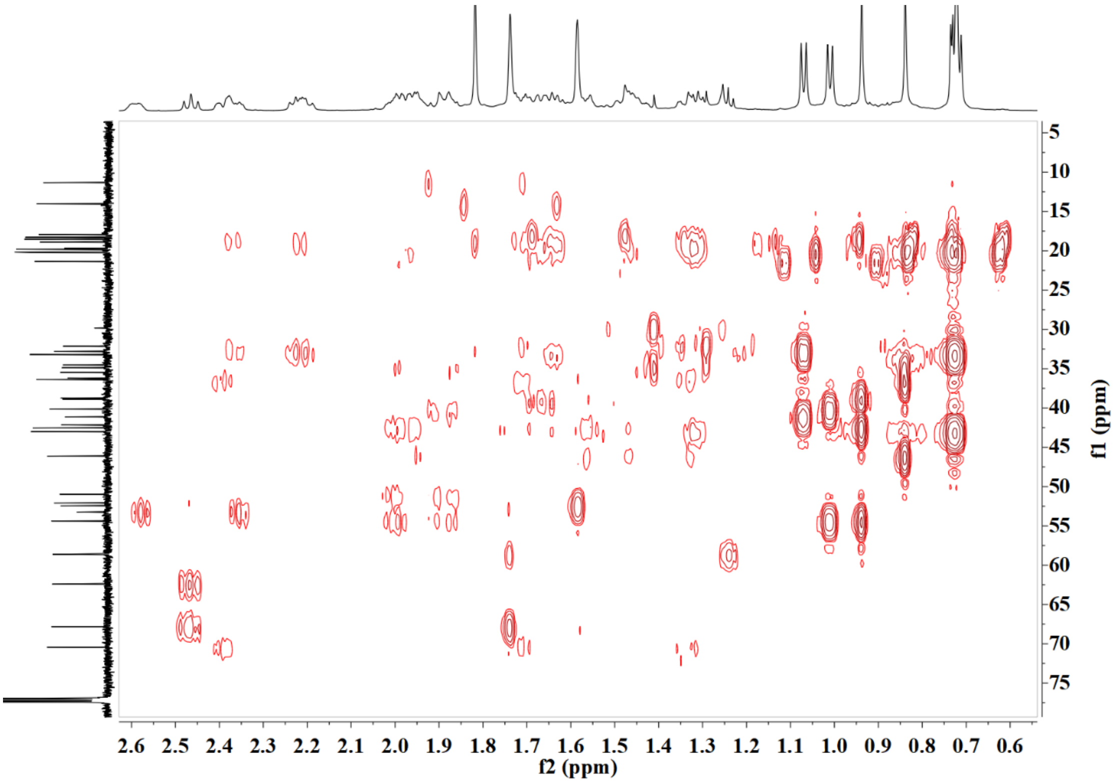

Figure S58. Partial enlarged HMBC (600 MHz, $CDCl_3$) spectrum of compound **4**

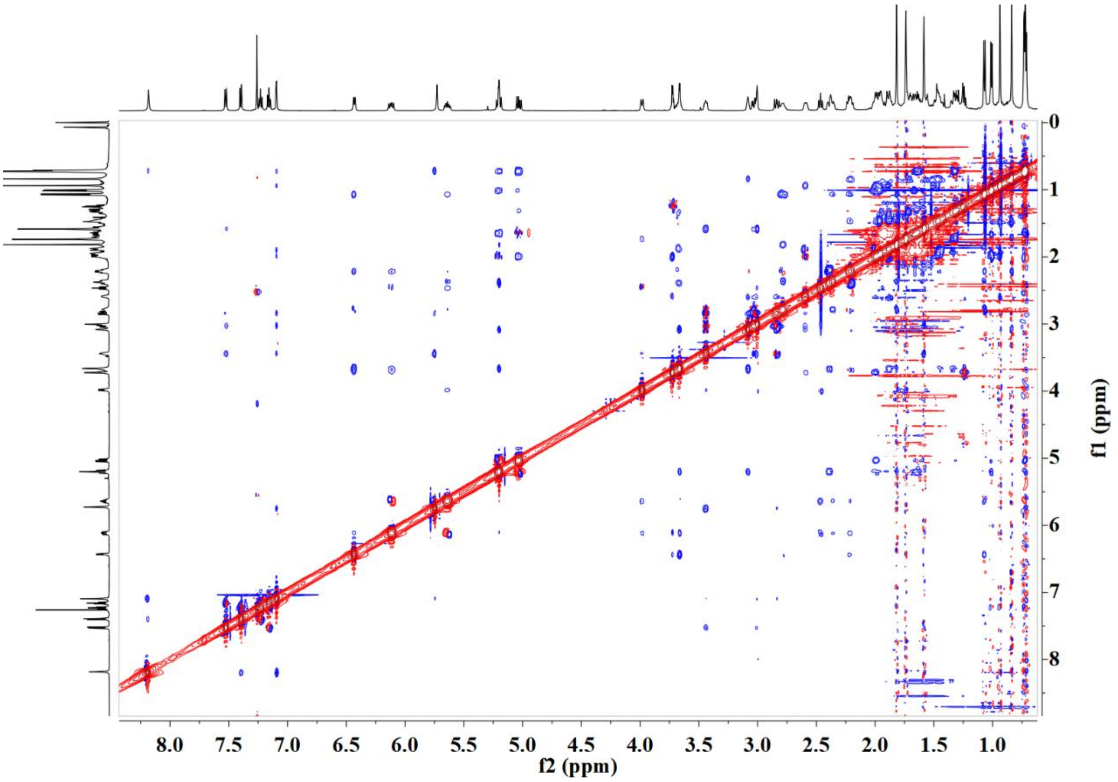

Figure S59. NOESY (600 MHz, $CDCl_3$) spectrum of compound **4**

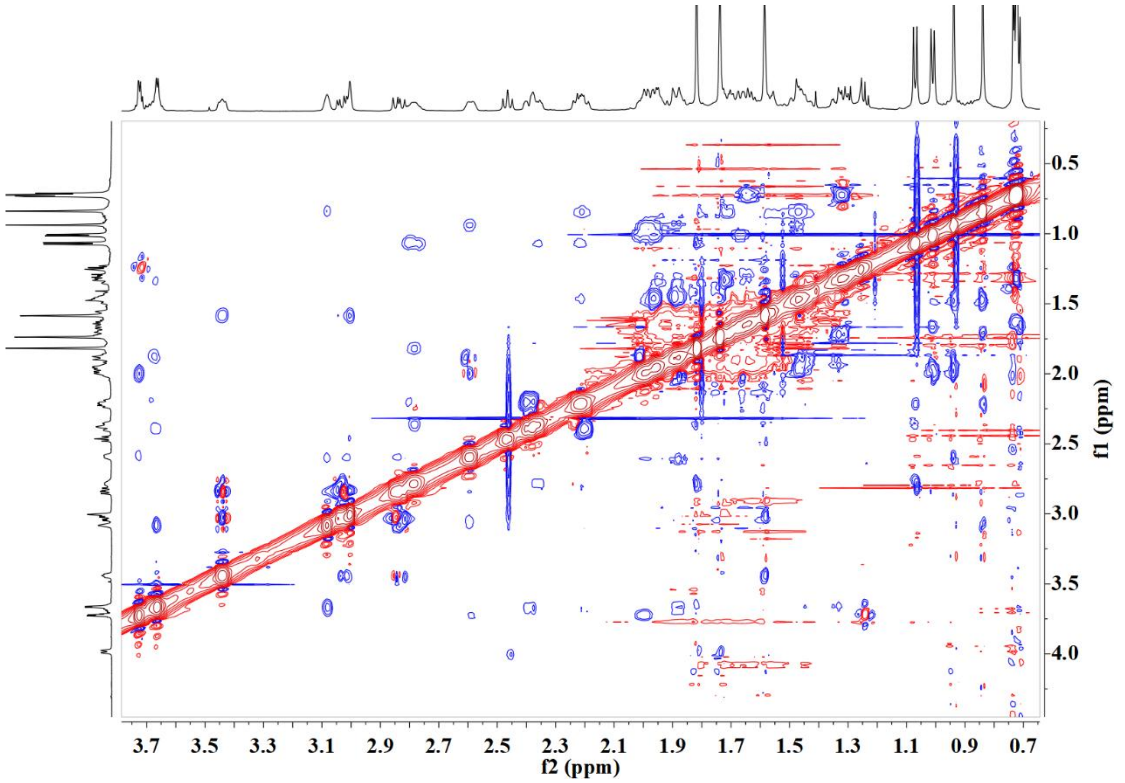

Figure S60. Partial enlarged NOESY (600 MHz, $CDCl_3$) spectrum of compound **4**

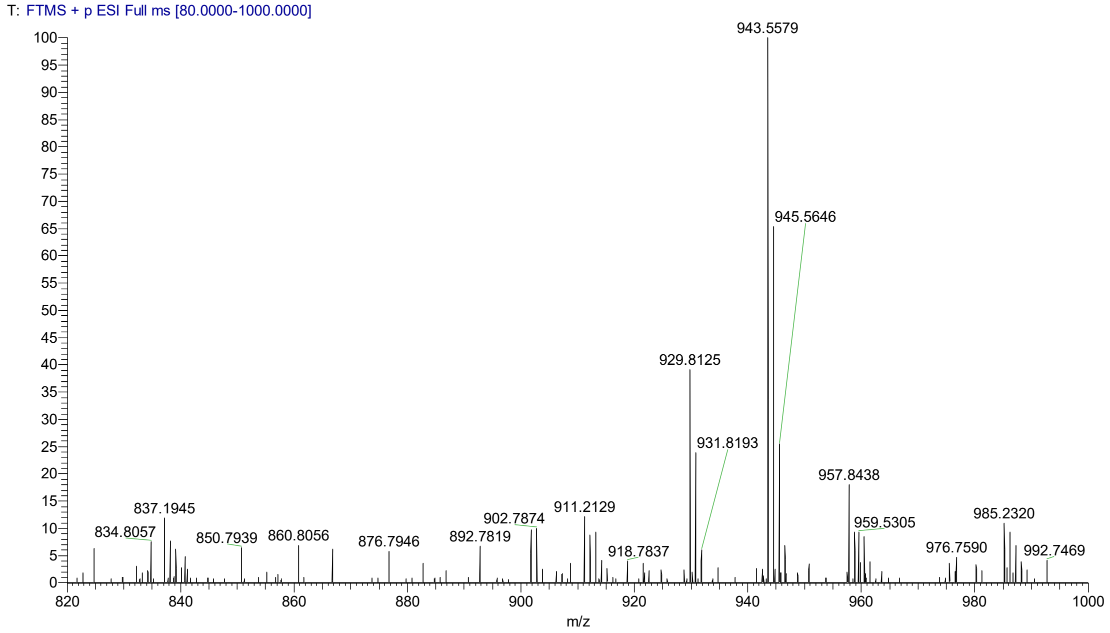

Figure S61. HRESIMS spectrum of compound **4**

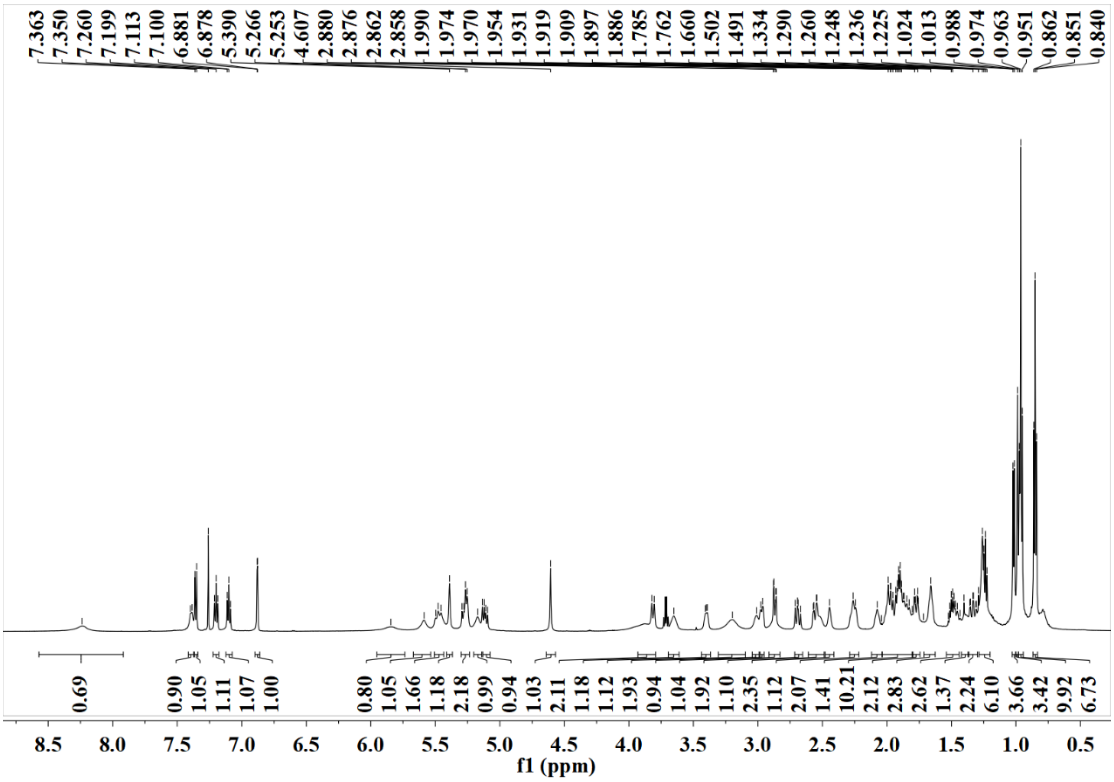

Figure S62. ^{1}H NMR (600 MHz, $CDCl_3$) spectrum of compound **5**

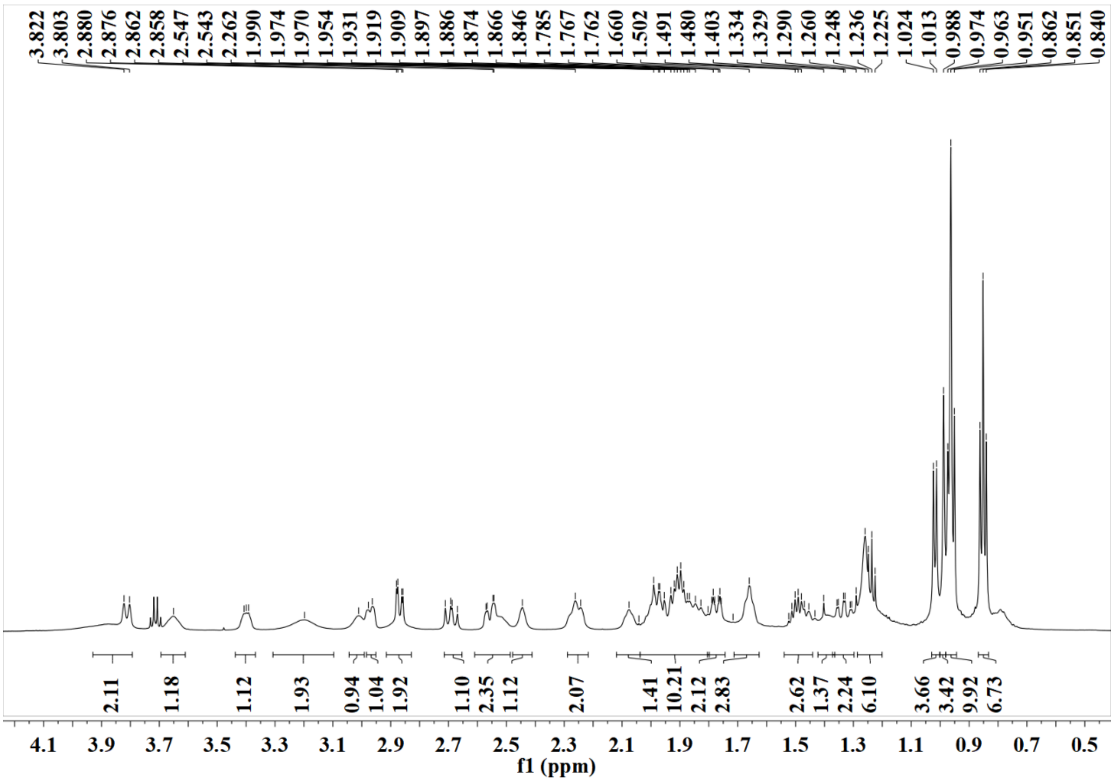

Figure S63. Partial enlarged ^{1}H NMR (600 MHz, $CDCl_3$) spectrum of compound **5**

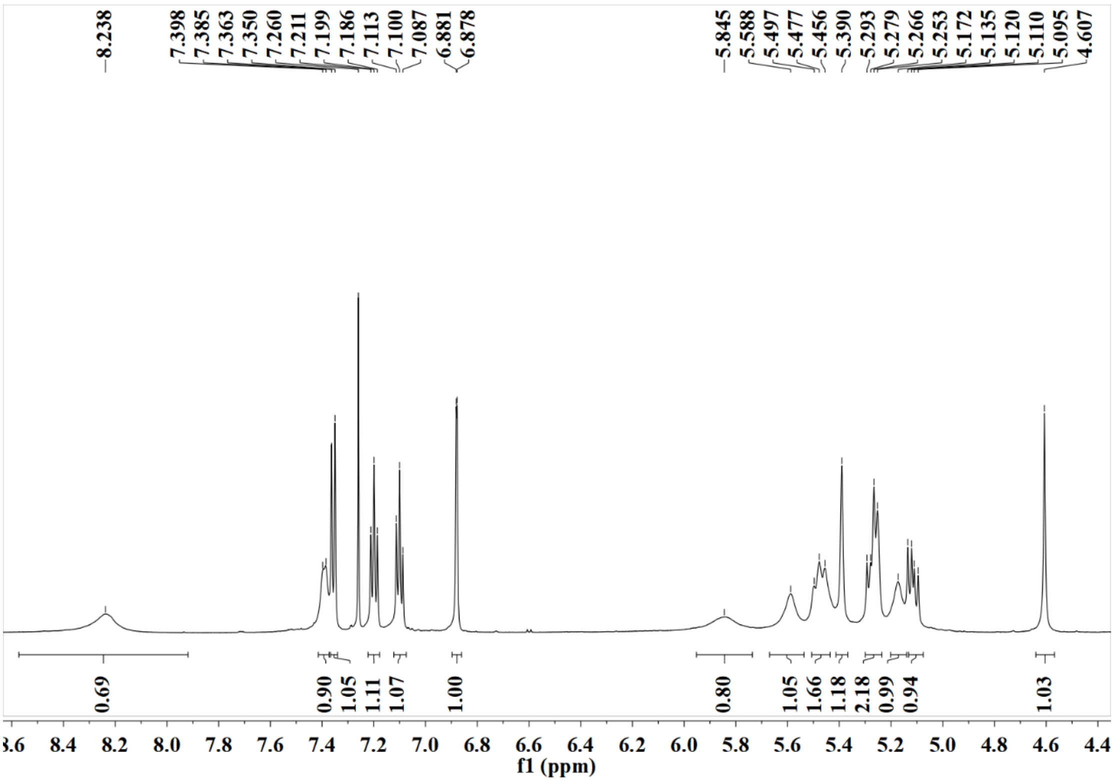

Figure S64. Partial enlarged ^{1}H NMR (600 MHz, $CDCl_3$) spectrum of compound **5**

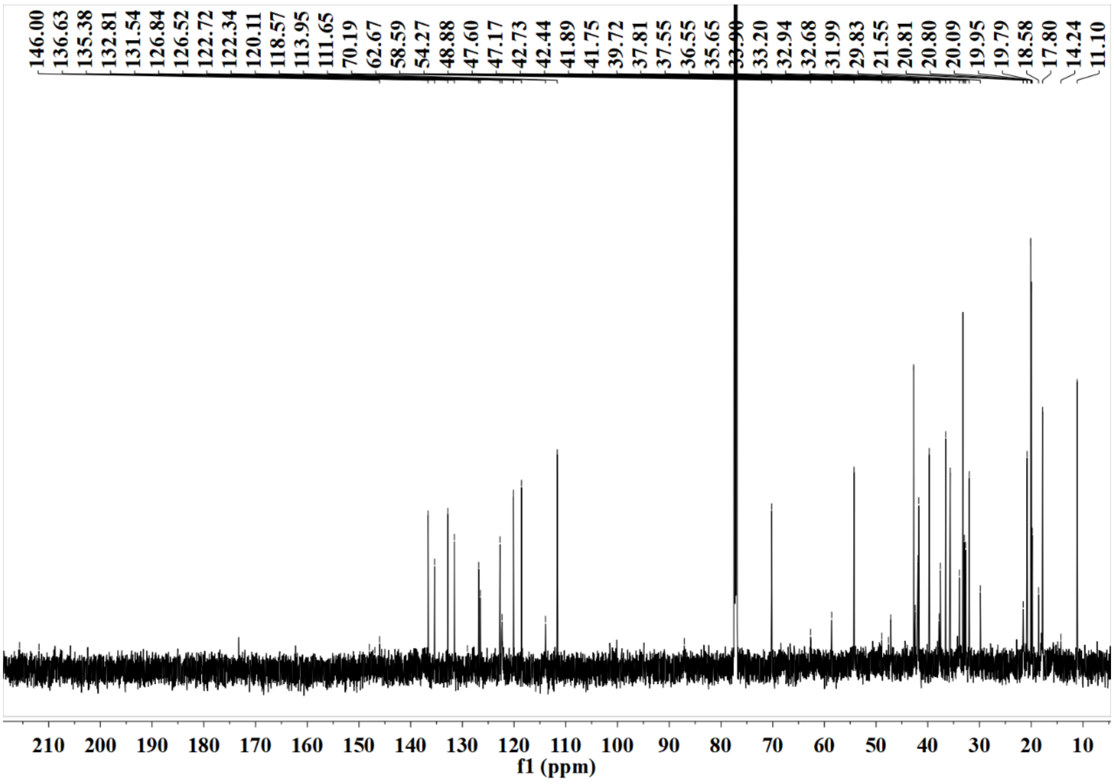

Figure S65. ^{13}C NMR (150 MHz, $CDCl_3$) spectrum of compound **5**

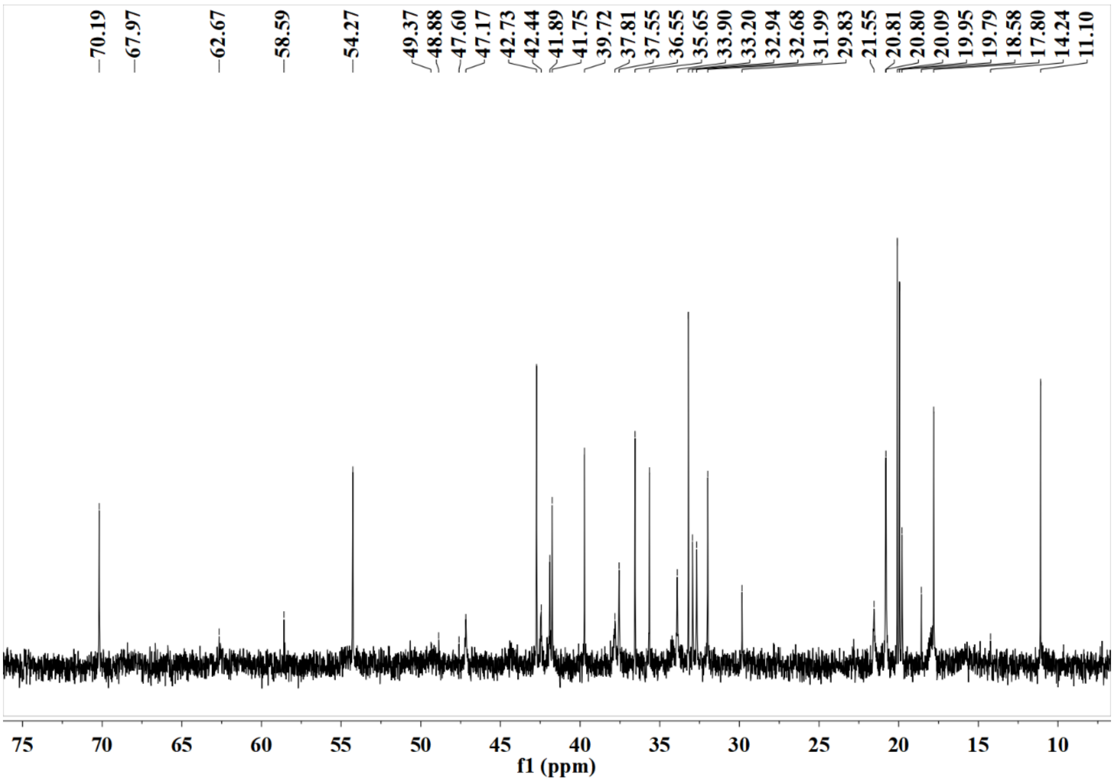

Figure S66. Partial enlarged ^{13}C NMR (150 MHz, $CDCl_3$) spectrum of compound **5**

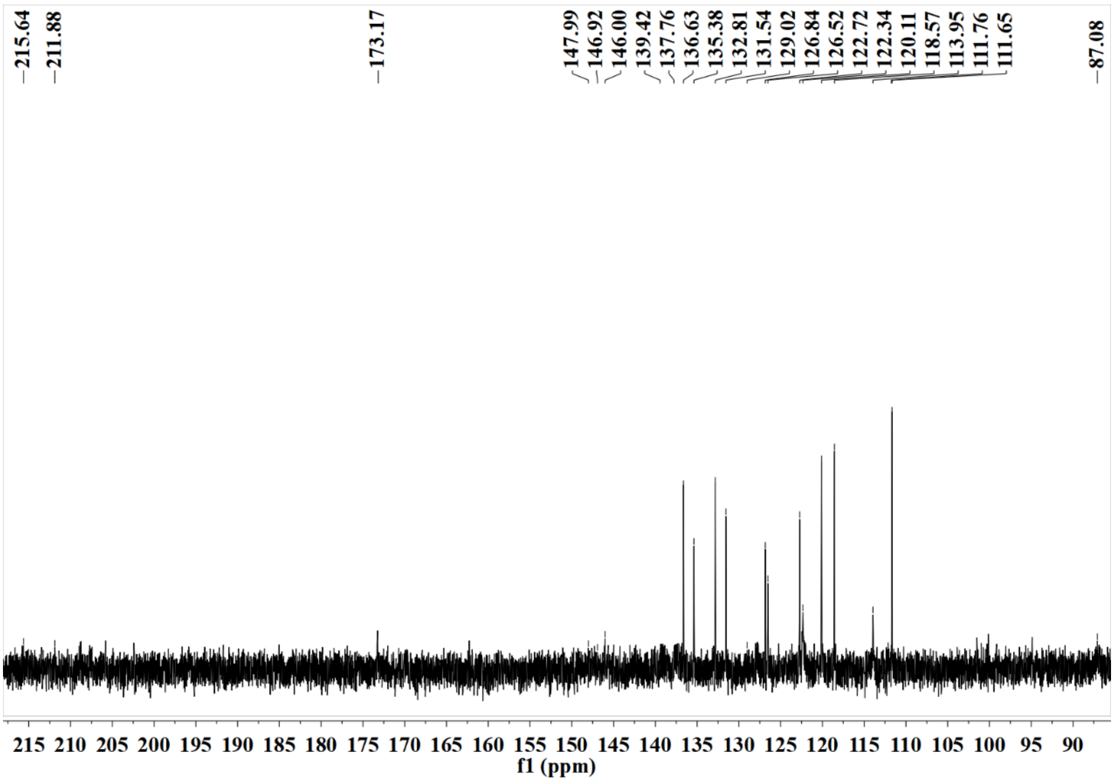

Figure S67. Partial enlarged ^{13}C NMR (150 MHz, $CDCl_3$) spectrum of compound **5**

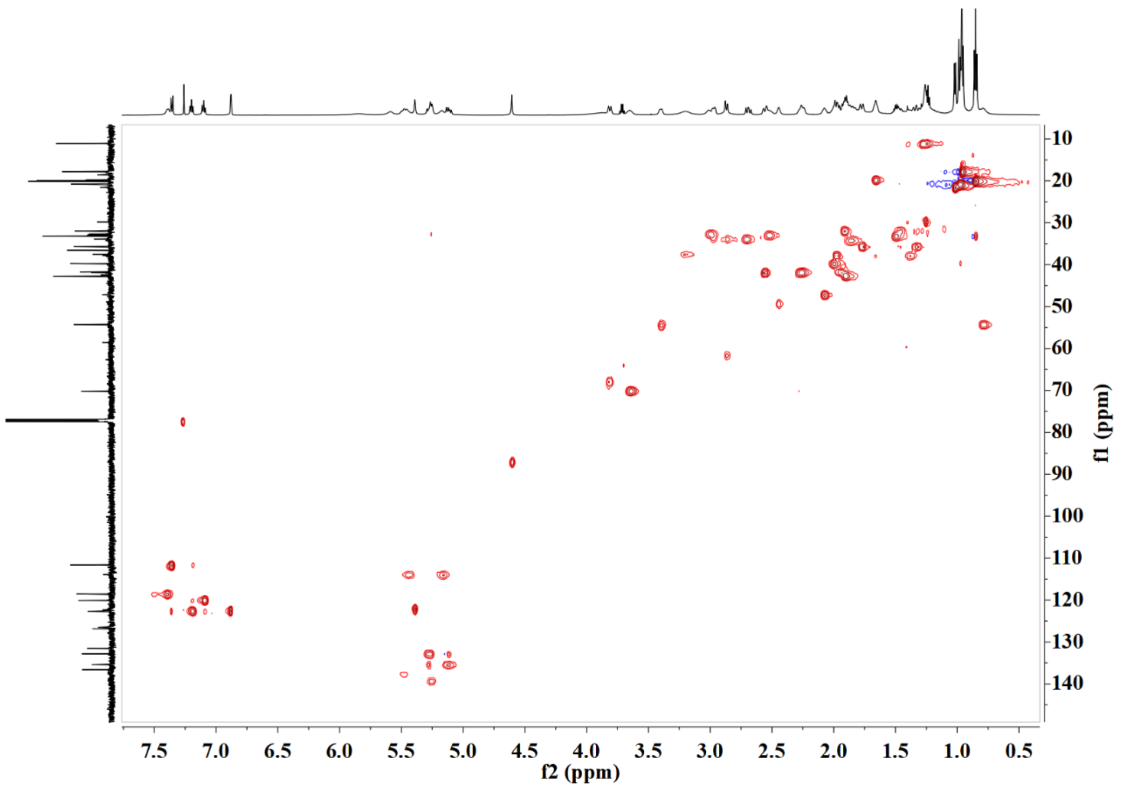

Figure S68. HSQC (600 MHz, $CDCl_3$) spectrum of compound **5**

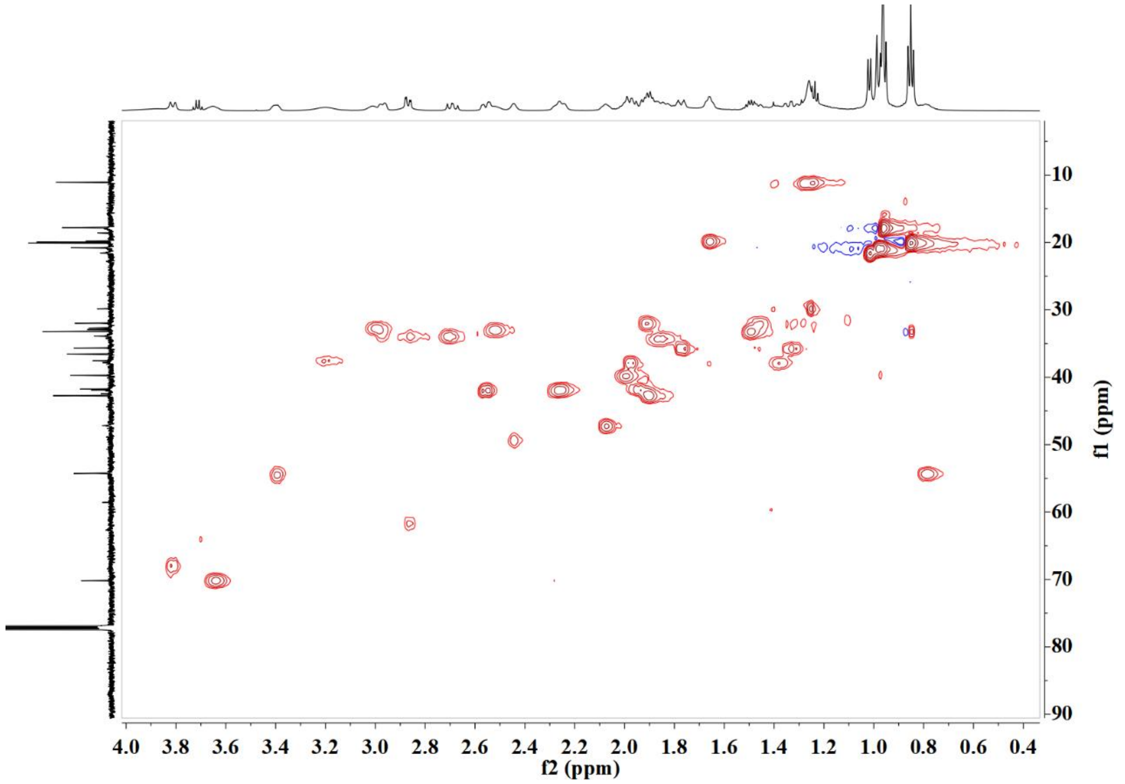

Figure S69. Partial enlarged HSQC (600 MHz, $CDCl_3$) spectrum of compound **5**

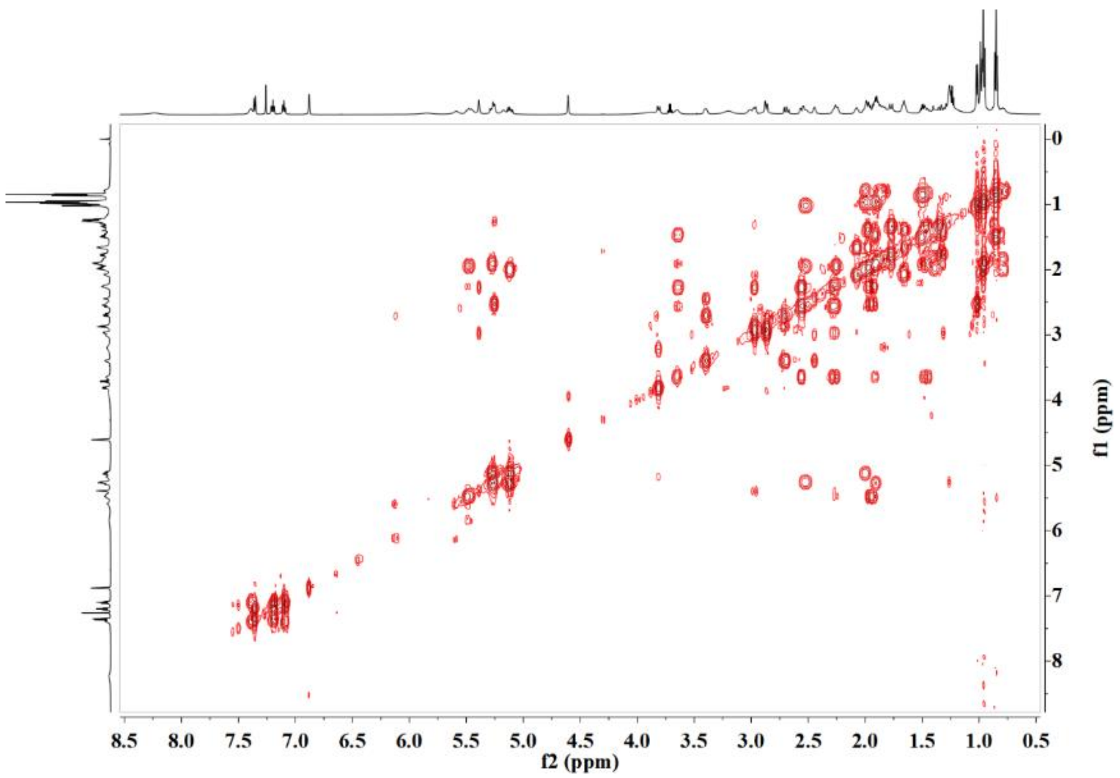

Figure S70. ^{1}H-^{1}H COSY (600 MHz, $CDCl_3$) spectrum of compound **5**

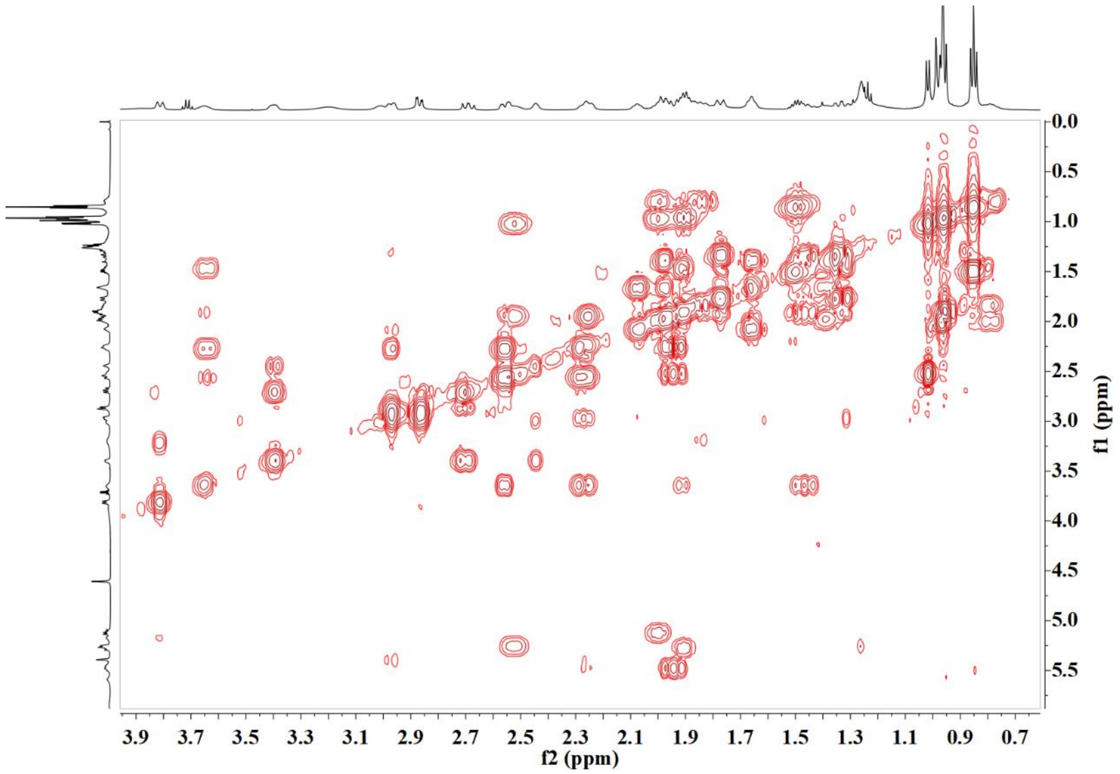

Figure S71. Partial enlarged ^{1}H-^{1}H COSY (600 MHz, $CDCl_3$) spectrum of compound **5**

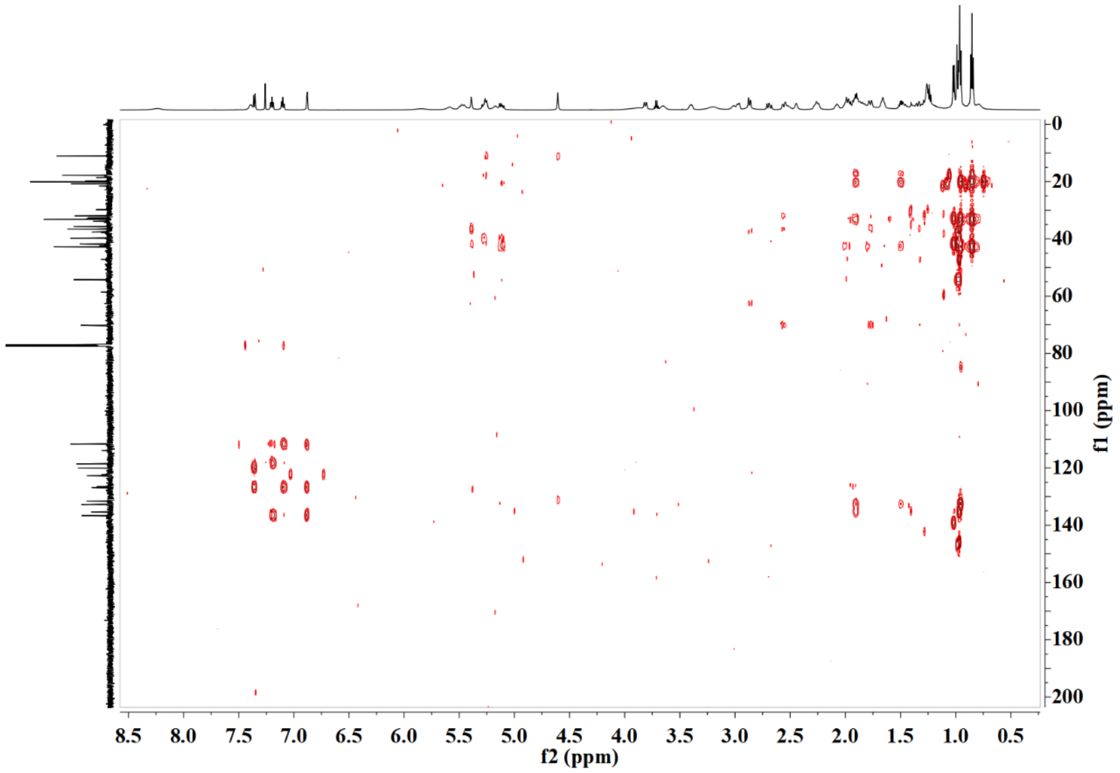

Figure S72. HMBC (600 MHz, $CDCl_3$) spectrum of compound **5**

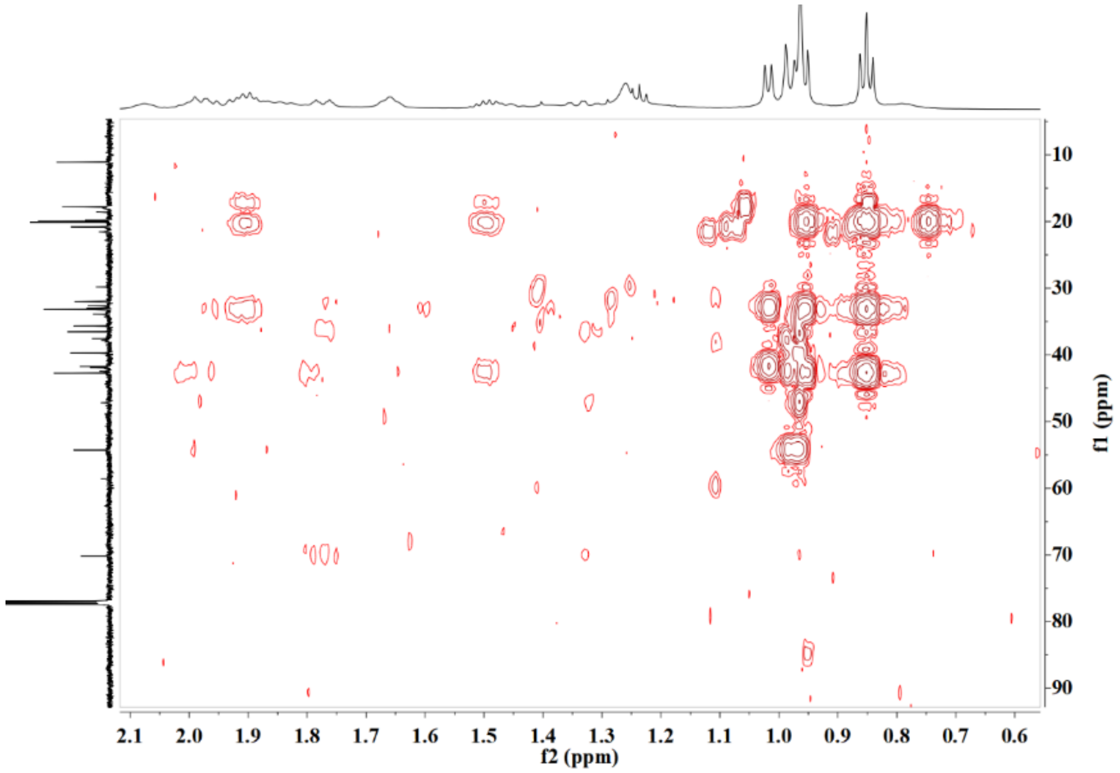

Figure S73. Partial enlarged HMBC (600 MHz, $CDCl_3$) spectrum of compound **5**

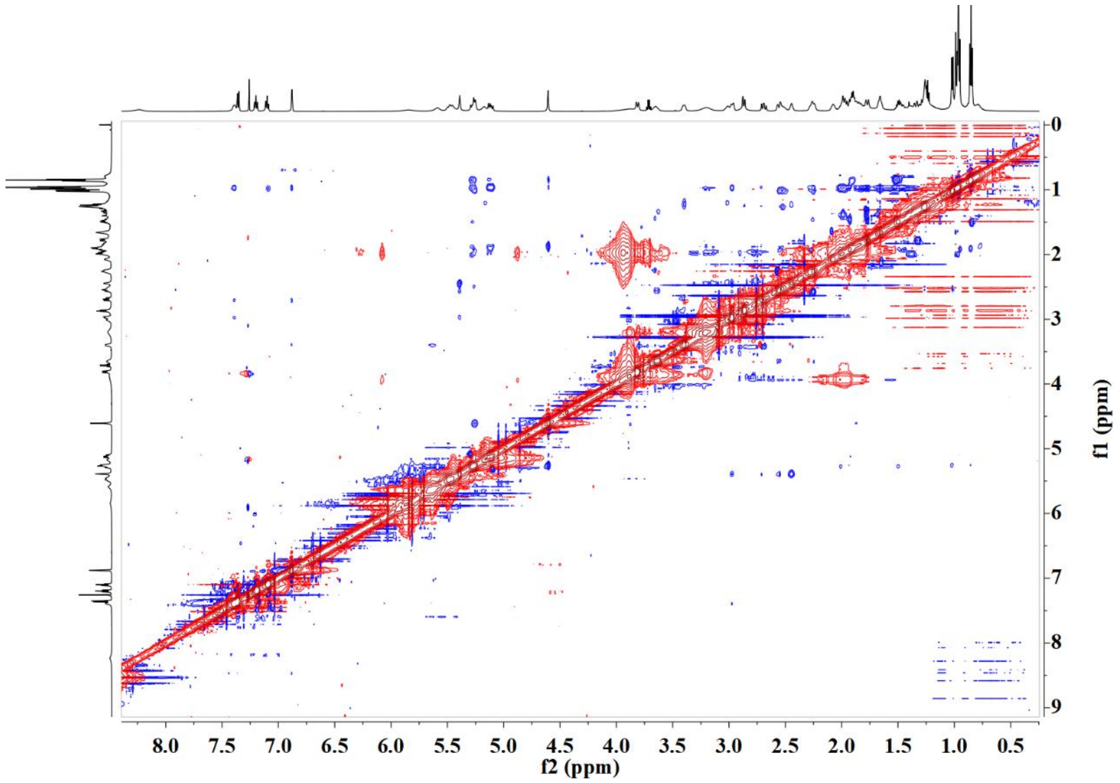

Figure S74. NOESY (600 MHz, $CDCl_3$) spectrum of compound **5**

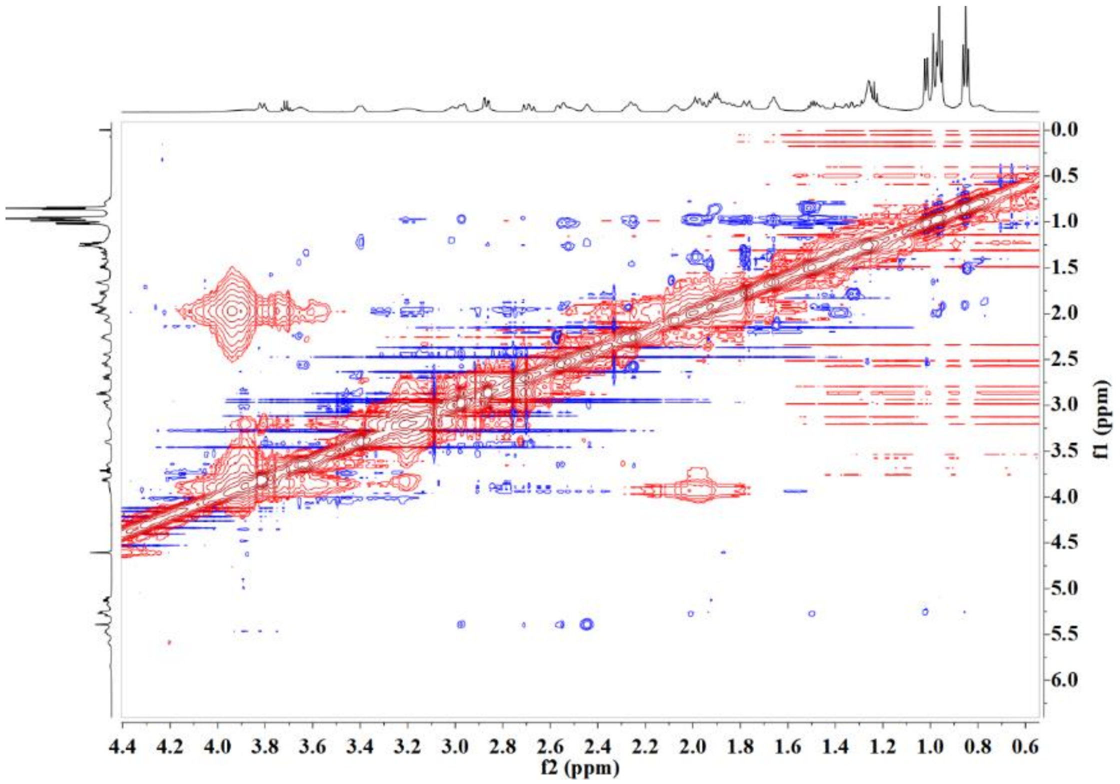

Figure S75. Partial enlarged NOESY (600 MHz, $CDCl_3$) spectrum of compound **5**

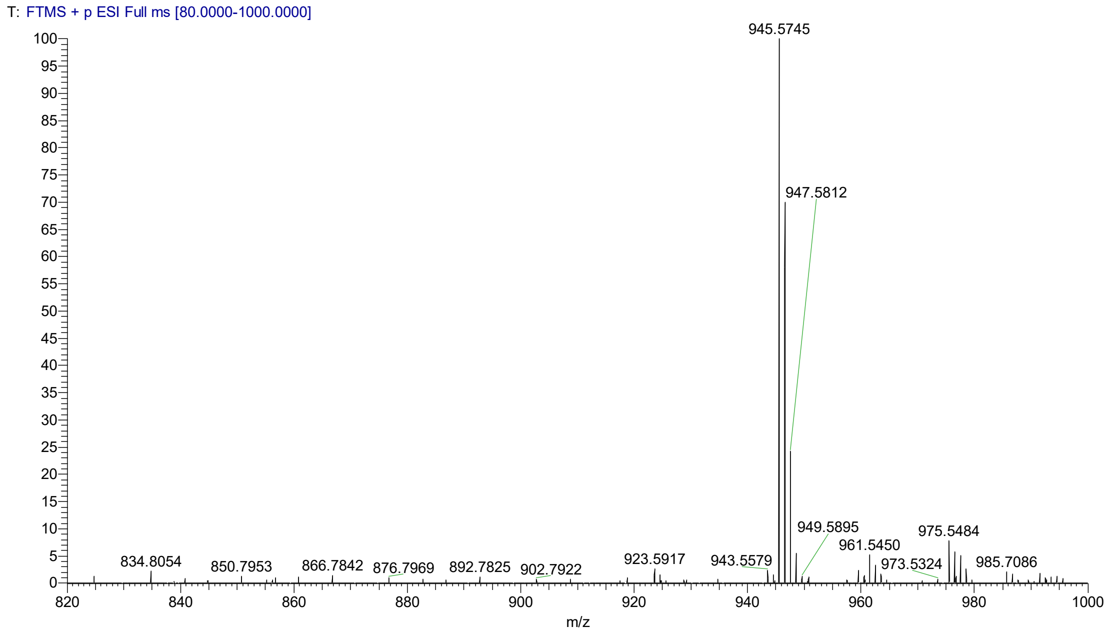

Figure S76. HRESIMS spectrum of compound **5**

Table S1. Comparison of ^{1}H and ^{13}C NMR data of the stereocenters and adjacent atoms of **1** with ergochaeglobosin E and chaetoglobosin Fex

No.	**1**		Ergochaeglobosin E		chaetoglobosin Fex	
	δ_H (*J* in Hz)	δ_C	δ_H (*J* in Hz)	δ_C	δ_H (*J* in Hz)	δ_C
1		173.6		173.8		174.1
3	3.50, m	52.5	3.40, m	52.8	3.45, m	52.5
4	2.71, m	48.2	2.66, m	47.7	2.80, m	47.7
5	2.85, m	32.2	2.85, m	32.3	2.43, d (13.2)	33.6
6		148.1		148.2		148.4
7	3.94, d (10.8)	70.2	3.90,d (10.6)	70.0	3.91, d (10.8)	70.1
8	2.66, m	49.3	2.63, m	49.6	1.71, m	49.7
9		62.8		63.0		62.9
10a	2.85, dd (14.4, 4.8)	33.3	2.93, dd (14.4, 3.6)	34.5	2.92, dd (14.4, 3.6)	32.3
10b	2.69, dd (14.4, 9.6)		2.56, dd (14.4, 9.3)		2.68, m	
11	1.10, d (6.6)	14.0	1.18,d (6.6)	14.2	1.15, d (6.6)	14.05
13	6.31, dd (13.8, 9.6)	128.8	6.23, dd (15.1, 9.9)	128.7	6.20, dd (15.0, 9.6)	128.7
15a	2.49, m	41.1	2.48, m	41.2	2.37, m	41.2
15b	2.12, m		2.12, m		1.80, m	
16	2.80, m	33.7	2.80, m	33.3	2.05, m	33.9
17	6.19, d (9.6)	149.4	6.16, d (9.5)	149.2	6.12, d (9.0)	149.2
19		203.7		203.6		203.7
20	4.78, m	71.5	4.71, t (5.1)	71.7	4.69, m	71.7
21a	1.91, m	31.6	1.86, m	31.6	2.61, m	31.6
21b	1.91, m		1.86, m		2.07, m	
23		208.0		208.0		208.0
24	1.06, d (6.6)	20.1	1.05, d (6.5)	20.1	1.02, d (6.6)	20.0

Table S2. Comparison of ^{1}H and ^{13}C NMR data of the stereocenters and adjacent atoms of **1** with ergochaeglobosin E and ergosta-4,6,8(14),22-tetraen-3*β*-ol

No.	**1**		Ergochaeglobosin E		ergosta-4,6,8(14),22-tetraen-3*β*-ol	
	δ_H (*J* in Hz)	δ_C	δ_H (*J* in Hz)	δ_C	δ_H (*J* in Hz)	δ_C
1″a	1.67, m	31.7	1.52, m	30.4	1.69, ddd (13.0, 3.5, 3.5)	33.6
1″b	1.40, m		1.30, m		1.38, ddd (13.0, 13.0, 2.5)	
5″		146.8		149.1		145.2
8″		124.7		124.7		124.8
9″	2.12, m	46.1	2.15, m	45.5	1.95, m	45.5
10″		35.9		36.0		35.8
11″a	1.64, m	19.7	1.59, m	19.7	1.58, m	19.1
11″b	1.57, m		1.59, m		1.51, m	
12″a	2.05, m	36.7	2.00, m	36.5	2.01,ddd (12.6, 3.5, 3.5)	36.4
12″b	1.31, m		1.28, m		1.25,ddd (13.0, 13.0, 3.4)	
13″		43.9		43.9		43.6
14″		150.6		151.0		149.8
16″a	1.80, m	28.0	1.78, m	28.0	1.76, m	27.8
16″a	1.48, m		1.47, m		1.20, ddd (11.8, 9.2, 7.6)	
17″	1.24, m	56.2	1.23, m	56.1	1.20, ddd (11.8, 9.2, 7.6)	55.9
18″	0.96, s	19.4	0.93, s	19.3	0.92, s	19.2
19″	0.95, s	18.3	0.91, s	17.8	0.89, s	18.3
20″	2.14, m	39.6	2.11, m	39.5	2.10, m	39.4
21″	1.06, d (6.6)	21.4	1.03, d (6.7)	21.4	1.04, d (6.7)	21.2
22″	5.24, dd (15.6, 7.2)	135.4	5.21, dd (15.2, 7.4)	135.4	5.20, dd (15.4,7)	135.3
23″	5.24, dd (15.6, 7.2)	132.4	5.25, dd (15.2, 7.0)	132.5	5.23, dd (15.4,7)	132.2
24″	1.88, m	43.0	1.88, m	43.0	1.86, m	42.9
25″	1.49, m	33.3	1.48, m	33.3	1.46, m	33.1
28″	0.94, d (7.8)	17.8	0.94, d (6.7)	17.7	0.92, d (6.8)	17.6

Table S3. The coordinates for the lowest-energy conformer of **(**3''*R*)**-1** in calculation

Atom	X	Y	Z	Atom	X	Y	Z
C	-2.07574	7.1761	0.43439	H	1.24145	6.3108	0.38927
C	-0.68044	7.28094	0.60326	H	-3.7298	5.95925	-0.20826
C	0.16479	6.23026	0.26316	H	1.13898	3.72032	-0.80264
C	-0.42058	5.0685	-0.24905	H	-3.97282	3.72192	-1.96875
C	-1.82548	4.93578	-0.42589	H	-3.28348	2.10692	-1.88573
C	-2.65239	6.01832	-0.0742	H	-4.51481	3.69316	0.41648
N	0.15219	3.88794	-0.67012	H	-2.81328	2.17111	1.40055
C	-0.8285	3.02238	-1.13183	H	-7.5054	0.0028	0.1578
C	-2.06431	3.62569	-0.98671	H	-6.71113	0.89149	2.93703
C	-3.4067	3.03812	-1.31938	H	-7.73442	2.13037	-0.36992
C	-4.28976	2.74436	-0.08267	H	-5.81978	-1.42844	2.29866
N	-3.62709	1.87577	0.87737	H	-3.17924	-1.30326	-0.87689
C	-5.60345	1.97762	-0.43065	H	-4.23209	-1.91413	0.37001
C	-4.22568	0.6901	1.14996	H	-8.3201	-2.1857	0.68651
C	-5.42684	0.54801	0.193	H	-8.40308	-3.65001	2.79909
O	-3.84677	-0.11949	1.99038	H	-6.64751	-3.58271	2.89147
C	-6.72466	0.04597	0.92592	H	-5.63135	-3.00143	-1.53549
C	-7.21191	1.08111	1.97593	H	-4.30301	-2.64535	-2.62548
C	-6.9727	2.52208	1.5546	H	-8.31022	-4.95266	0.71273
C	-6.89637	2.69741	0.05887	H	-5.3879	-4.25047	0.83494
C	-5.14355	-0.48273	-0.94109	H	-3.56609	-4.11235	-0.09936
C	-6.62854	-1.31424	1.57934	H	-6.72854	4.53654	2.19922
C	-4.20723	-1.65174	-0.69117	H	-6.8258	3.26376	3.53214
C	-7.50445	-2.30977	1.40151	H	-8.96646	1.50413	2.71433
C	-7.49056	-3.59692	2.18673	H	-2.76433	-5.04887	-2.49356
C	-4.5489	-2.85181	-1.58028	H	-7.89872	-6.57888	-0.76215
C	-7.41509	-4.89833	1.34753	H	-7.17869	-6.23549	-2.35038
C	-6.19038	-4.88474	0.46517	H	-6.52053	-7.52465	-1.36143
C	-5.98599	-5.55407	-0.68758	H	-7.41602	-7.06278	1.70672
C	-3.80375	-4.15147	-1.17157	H	-8.30784	-6.12338	2.92095
C	-4.71307	-5.36713	-1.43815	H	-6.53485	-6.13194	2.92749
C	-6.83413	3.48931	2.4672	H	-6.97733	4.14455	-1.55833
O	-8.62066	0.83805	2.09905	H	-7.99737	4.55371	-0.16897
O	-5.72305	-0.35581	-2.00434	H	-6.25186	4.8004	-0.0845
O	-2.59193	-4.31113	-1.87273	H	0.75373	2.40124	-3.40637
O	-4.3925	-6.13115	-2.34425	H	1.10619	-0.22859	0.80955
C	-6.95872	-6.52126	-1.31547	H	1.23542	1.50677	0.55679
C	-7.41895	-6.12983	2.27907	H	-0.49039	-0.41011	-1.10007
C	-7.03102	4.13065	-0.46381	H	-1.08836	0.67656	0.14277
C	-0.44617	1.66754	-1.69251	H	3.01036	1.92195	-4.01256
C	0.81957	1.78044	-2.51227	H	5.25335	1.26865	-3.36587

Atom	X	Y	Z	Atom	X	Y	Z
C	2.01257	1.23163	-2.19508	H	5.52505	-0.32916	2.3785
C	2.21844	0.32248	-0.97797	H	5.5867	1.21888	1.54387
C	1.07588	0.54983	0.03862	H	3.25734	0.36147	1.77663
C	-0.30964	0.55814	-0.61773	H	3.68068	-1.09437	0.89943
C	3.17966	1.46066	-3.04109	H	9.20338	0.60299	-1.18724
C	4.43405	1.11977	-2.66731	H	8.79368	-1.09582	-1.07673
C	4.73462	0.57854	-1.34885	H	7.14307	-0.66118	-2.70132
C	3.5992	0.69667	-0.33622	H	7.25711	1.07222	-2.46269
C	5.95709	0.09665	-1.02878	H	8.88145	-1.55656	1.34745
C	6.35017	-0.42088	0.35449	H	10.1151	1.26086	1.36599
C	5.37524	0.149	1.40504	H	12.2161	0.25972	1.32327
C	3.90777	-0.02579	0.98416	H	10.77443	-2.46223	1.45997
C	7.82754	0.10787	0.45952	H	12.77925	-3.44871	0.50289
C	8.38093	-0.08571	-0.97428	H	11.49546	-1.34542	-1.32325
C	7.18154	0.11751	-1.92813	H	12.33326	-2.81237	-1.8588
C	8.78264	-0.47996	1.53175	H	10.8209	-2.93896	-0.94581
C	10.14617	0.16883	1.39881	H	14.48577	-2.15472	-0.69813
C	11.355	-0.40873	1.3726	H	14.50591	-1.6896	1.00568
C	11.70285	-1.87654	1.47212	H	13.75925	-0.62559	-0.19941
C	12.55477	-2.39594	0.27262	H	2.33001	-1.84234	-0.62186
C	11.75529	-2.37142	-1.03783	H	1.28341	-1.39668	-1.97308
C	13.89988	-1.67281	0.09336	H	3.03647	-1.33489	-2.16574
C	2.21911	-1.14967	-1.46322	H	6.61419	-2.36407	1.34568
C	6.31932	-1.971	0.36536	H	5.31704	-2.34746	0.14072
C	8.29035	-0.29099	2.983	H	6.99639	-2.3973	-0.38254
C	12.39491	-2.1543	2.82402	H	8.09921	0.76678	3.20276
H	7.75057	1.19095	0.64922	H	9.05045	-0.64309	3.68854
H	3.52542	1.77385	-0.10165	H	7.36906	-0.84664	3.17984
H	-5.68876	1.87124	-1.51519	H	11.72252	-1.92465	3.65752
H	-1.26515	1.37893	-2.36541	H	13.29448	-1.5415	2.95237
H	-2.70854	8.017	0.70524	H	12.69128	-3.20761	2.90483
H	-0.25746	8.19844	1.00302				

Table S4. The coordinates for the lowest-energy conformer of **(**3''*S*)-**1** in calculation

Atom	X	Y	Z	Atom	X	Y	Z
C	6.47573	-6.09708	0.45173	H	5.07661	-2.39044	2.60937
C	5.5985	-6.89901	-0.30499	H	6.50175	-1.74564	0.65014
C	4.32133	-6.45579	-0.63322	H	6.78641	-0.30005	2.68377
C	3.94238	-5.18672	-0.18626	H	4.1352	-0.68077	-0.4872
C	4.8098	-4.35748	0.5756	H	4.75126	2.08075	-1.54639
C	6.09344	-4.83507	0.89249	H	7.46973	2.47339	-0.23474
N	2.7622	-4.49239	-0.34712	H	5.36344	0.18929	-2.26262
C	2.83952	-3.26397	0.29319	H	5.44233	3.94702	0.79322
C	4.0892	-3.14095	0.87197	H	3.66627	1.37142	2.86547
C	4.61695	-1.99717	1.69045	H	3.4215	2.9536	2.2183
C	5.67544	-1.1102	0.98255	H	4.00371	4.2384	-1.9046
N	6.19437	-0.09126	1.89009	H	4.42094	6.69911	-1.10685
C	5.10675	-0.27462	-0.20049	H	4.29864	6.10348	0.54975
C	5.83614	1.19127	1.62897	H	1.13889	0.90184	2.04772
C	4.92326	1.17159	0.3874	H	1.50651	1.65534	3.5952
O	6.17388	2.17631	2.27673	H	2.13626	6.0311	-1.62125
C	5.34911	2.26305	-0.64657	H	1.82534	5.16811	1.30827
C	6.83745	2.08637	-1.04744	H	1.08255	3.90425	2.61395
C	7.18656	0.62512	-1.30254	H	8.75284	-0.78799	-1.56288
C	5.98877	-0.28131	-1.48968	H	9.27079	0.94482	-1.18726
C	3.42954	1.41452	0.74898	H	6.78637	3.7245	-2.08493
C	5.06362	3.67144	-0.18979	H	-1.14621	2.28866	2.32814
C	3.05947	1.91888	2.1352	H	1.06944	2.72735	-1.92353
C	4.37344	4.5507	-0.92529	H	-0.40516	3.68196	-1.84821
C	3.95772	5.92391	-0.47848	H	1.11669	4.44998	-2.35094
C	1.57412	1.79448	2.51123	H	2.53894	8.29297	-0.64759
C	2.41423	6.11577	-0.56488	H	2.26493	7.656	0.98293
C	1.70257	5.05247	0.23238	H	0.93654	7.68514	-0.18896
C	0.9592	4.02137	-0.22243	H	6.96267	-2.2551	-1.34076
C	0.6292	2.98831	2.22414	H	5.37732	-2.27205	-2.11227
C	0.27233	3.10498	0.72863	H	6.78449	-1.63356	-2.98342
C	8.4624	0.23554	-1.34597	H	1.93362	-1.46219	0.88511
O	7.09459	2.81574	-2.24958	H	0.62545	-3.55367	1.83151
O	2.58115	1.18819	-0.09713	H	-0.22411	-0.67918	-2.14213
O	-0.57876	2.79647	2.93986	H	0.03294	-0.21896	-0.46797
O	-0.65168	2.39098	0.34198	H	1.28412	-2.66293	-1.83731
C	0.67436	3.71829	-1.67267	H	2.0973	-1.14485	-1.47918
C	2.01314	7.51947	-0.07606	H	-1.69375	-4.12913	2.03239
C	6.29906	-1.69091	-2.00344	H	-4.01154	-3.51987	1.66056
C	1.65149	-2.33711	0.28094	H	-2.11269	-0.50918	0.55484
C	0.43691	-2.96726	0.93172	H	-4.48403	1.29514	-1.83609
C	-0.83913	-2.79231	0.52826	H	-4.20902	1.05782	-0.1149

Atom	X	Y	Z	Atom	X	Y	Z
C	-1.2223	-1.97886	-0.71308	H	-2.13577	0.73483	-1.42857
C	-0.03694	-1.0839	-1.14014	H	-2.89579	-0.53332	-2.37987
C	1.3074	-1.81999	-1.13479	H	-6.36157	0.47654	0.6008
C	-1.94162	-3.37695	1.28517	H	-7.95115	-1.27786	1.05753
C	-3.23144	-3.01477	1.09642	H	-7.94308	-1.83445	-0.60333
C	-3.61341	-1.94588	0.18294	H	-6.28381	-3.36819	0.06638
C	-2.45273	-1.09146	-0.31833	H	-5.98727	-2.5095	1.56613
C	-4.90481	-1.68025	-0.11813	H	-8.05984	0.22988	-1.92728
C	-5.36273	-0.53484	-1.02018	H	-8.695	1.56393	0.77246
C	-4.25487	0.53607	-1.081	H	-10.36321	0.27145	-1.44109
C	-2.87639	-0.07048	-1.38587	H	-12.06125	1.70666	-0.32434
C	-6.6812	-0.08624	-0.29093	H	-13.1499	0.16064	1.19833
C	-7.30453	-1.42181	0.18756	H	-13.36198	-0.09489	-1.29169
C	-6.11806	-2.36706	0.48641	H	-12.05262	-1.28986	-1.27276
C	-7.71272	0.79168	-1.04881	H	-13.5623	-1.62553	-0.42309
C	-8.91608	1.05708	-0.16849	H	-11.22233	-0.83637	2.4262
C	-10.1821	0.76676	-0.48531	H	-10.66592	-1.62194	0.94065
C	-11.42648	1.07534	0.32063	H	-12.20689	-2.09718	1.67537
C	-12.28698	-0.19552	0.61559	H	-0.73613	-3.59123	-2.11915
C	-12.84441	-0.83352	-0.66709	H	-2.4119	-3.62253	-1.56289
C	-11.55164	-1.24368	1.46413	H	-1.8945	-2.4203	-2.75727
C	-1.59129	-2.96064	-1.8537	H	-4.78889	-1.53403	-2.88775
C	-5.67636	-1.08023	-2.43737	H	-6.45467	-1.85075	-2.41435
C	-7.15389	2.14424	-1.53812	H	-6.01941	-0.27942	-3.10322
C	-11.15094	1.88483	1.59569	H	-7.95812	2.74651	-1.97474
H	7.46564	-6.47418	0.69397	H	-6.37955	2.01885	-2.30004
H	5.92378	-7.88116	-0.63681	H	-6.7194	2.71853	-0.71031
H	3.64329	-7.07536	-1.21458	H	-10.66958	2.83997	1.35897
H	6.78045	-4.23081	1.48054	H	-10.49649	1.34915	2.29194
H	1.91901	-4.85412	-0.76699	H	-12.08819	2.10266	2.1207
H	3.79467	-1.35137	2.02288				

Table S5. The coordinates for the lowest-energy conformer of **(**3''*R*)-**2** in calculation

Atom	X	Y	Z	Atom	X	Y	Z
C	-8.95954	0.04676	0.35437	H	-5.36604	-1.53276	2.75877
C	-9.01082	1.37117	-0.12463	H	-5.26205	-2.5665	0.4914
C	-7.89353	2.19806	-0.08103	H	-4.23215	-3.85975	2.44922
C	-6.71218	1.67179	0.4569	H	-3.03283	-0.79755	0.0308
C	-6.63642	0.33153	0.93651	H	-1.06597	-2.79715	-1.60822
C	-7.78568	-0.47671	0.88205	H	-2.74483	-5.20462	-0.78734
N	-5.47113	2.26027	0.64119	H	-2.83853	-1.74156	-2.13405
C	-4.63225	1.31759	1.20655	H	-0.36197	-5.05477	0.3635
C	-5.28974	0.12809	1.40664	H	-0.17131	-1.63736	2.79329
C	-4.68821	-1.10964	2.00223	H	0.18013	-3.14463	1.9822
C	-4.34548	-2.23938	0.99419	H	1.01656	-3.92229	-2.13678
N	-3.758	-3.37373	1.69806	H	1.98793	-6.2959	-1.85523
C	-3.25397	-1.86316	-0.05244	H	1.66681	-6.19441	-0.12834
C	-2.43743	-3.60348	1.49338	H	1.78749	-1.79798	0.45334
C	-1.96863	-2.66744	0.35855	H	1.58143	-0.43023	1.53795
O	-1.76531	-4.43636	2.09521	H	3.69472	-4.54135	-1.72255
C	-1.38858	-3.51306	-0.84361	H	2.58455	-4.17193	1.04965
C	-2.5101	-4.38649	-1.48739	H	2.43566	-3.06832	2.75517
C	-3.78207	-3.59828	-1.77554	H	-5.79375	-3.70938	-2.44923
C	-3.67895	-2.1115	-1.52789	H	-4.8758	-5.31026	-2.35216
C	-0.8416	-1.68179	0.77532	H	-1.21555	-5.38793	-2.5264
C	-0.22425	-4.41793	-0.50863	H	3.64468	-0.74979	3.44294
C	0.17542	-2.06186	1.83858	H	5.52125	-3.38376	-1.18587
C	0.87504	-4.56482	-1.26458	H	5.4146	-1.67611	-0.70687
C	1.94639	-5.59682	-1.00691	H	6.36261	-2.78964	0.25881
C	1.56889	-1.52276	1.48935	H	4.1008	-6.71635	0.41131
C	3.37808	-5.03818	-0.7954	H	5.38794	-5.83008	-0.41997
C	3.3849	-4.03021	0.32729	H	4.33394	-6.9274	-1.33396
C	4.24136	-3.00677	0.52504	H	-4.72482	-0.22584	-1.7683
C	2.68436	-2.04919	2.42964	H	-5.09543	-1.4259	-3.01972
C	4.02197	-2.07346	1.66394	H	-5.8107	-1.56661	-1.40866
C	-4.87517	-4.23355	-2.20423	H	-5.98642	4.22804	0.32381
O	-2.05165	-4.92963	-2.72321	H	-4.2798	4.38701	2.10704
O	-0.7561	-0.61694	0.18579	H	-2.9811	3.14367	-2.54243
O	2.81729	-1.25347	3.58428	H	-3.16061	2.10847	-1.13403
O	4.86945	-1.23908	1.97476	H	-4.77116	4.66904	-1.65352
C	5.44404	-2.70526	-0.33336	H	-5.36299	3.02499	-1.87083
C	4.36114	-6.19588	-0.51778	H	-2.07238	5.21334	2.59439
C	-4.90065	-1.29081	-1.95035	H	0.31167	5.04598	2.19485
C	-5.08488	3.61558	0.19553	H	-1.10637	2.31598	0.03266
C	-3.99498	4.159	1.08025	H	1.51075	1.72304	-2.62302
C	-2.70531	4.30432	0.70923	H	1.09384	1.30397	-0.96374

Atom	X	Y	Z	Atom	X	Y	Z
C	-2.19492	4.00616	-0.70538	H	-0.89927	1.92658	-2.2946
C	-3.22873	3.15054	-1.47477	H	-0.22621	3.51584	-2.62059
C	-4.66873	3.63992	-1.28697	H	3.08753	1.79004	0.18358
C	-1.70939	4.76961	1.66921	H	4.45848	3.40091	1.3341
C	-0.37904	4.64952	1.45526	H	4.54544	4.45568	-0.06105
C	0.16779	3.97837	0.28509	H	2.68715	5.59329	0.83491
C	-0.85121	3.21382	-0.55574	H	2.36576	4.30744	1.98372
C	1.49646	3.94648	0.03037	H	4.97803	2.93217	-1.92613
C	2.13676	3.19992	-1.1391	H	5.46298	0.82757	0.27766
C	1.15473	2.13876	-1.6745	H	7.23713	2.88157	-1.14633
C	-0.25642	2.70956	-1.87798	H	7.72302	0.78171	1.0629
C	3.42645	2.62588	-0.44928	H	9.6504	1.91717	-1.01605
C	3.88049	3.77597	0.48542	H	10.90969	1.50427	1.08025
C	2.5888	4.50343	0.92844	H	10.18609	-0.10621	1.23167
C	4.59621	2.0973	-1.32161	H	11.29978	0.24764	-0.09723
C	5.72173	1.62299	-0.42649	H	9.75065	-0.41866	-1.88983
C	6.97693	2.08121	-0.44679	H	8.09108	0.20373	-1.96709
C	8.09927	1.61652	0.45247	H	8.5511	-0.93197	-0.69159
C	9.30926	1.08902	-0.37364	H	-2.90334	5.92557	-1.49962
C	10.49142	0.66666	0.5137	H	-1.23872	5.9741	-0.91243
C	8.90067	-0.07757	-1.28674	H	-1.61109	5.19201	-2.45819
C	-1.97147	5.35431	-1.43644	H	3.19312	4.98534	-1.891
C	2.51388	4.20768	-2.25602	H	3.00679	3.7005	-3.09392
C	4.20146	0.9564	-2.28218	H	1.62536	4.71324	-2.64614
C	8.49193	2.7659	1.40488	H	3.50385	1.29032	-3.05556
H	-9.85216	-0.57122	0.3082	H	3.72726	0.12811	-1.74079
H	-9.94059	1.75477	-0.53585	H	5.09223	0.5627	-2.78423
H	-7.94445	3.21679	-0.45449	H	9.22138	2.44543	2.15535
H	-7.75922	-1.49937	1.25148	H	7.6094	3.13982	1.93455
H	-3.61439	1.58284	1.45328	H	8.92748	3.60688	0.84897
H	-3.76481	-0.84694	2.53501				

Table S6. The coordinates for the lowest-energy conformer of (3"*S*)-**2** in calculation

Atom	X	Y	Z	Atom	X	Y	Z
C	5.24746	-3.21296	-3.55491	H	5.17421	-4.46015	0.68091
C	4.17927	-2.59405	-4.23576	H	5.54212	-3.25008	2.7737
C	3.01	-2.24094	-3.57254	H	3.27467	-2.11281	3.06672
C	2.93084	-2.51733	-2.20081	H	6.17969	-2.09629	0.19696
C	4.00155	-3.13328	-1.49188	H	7.18491	0.81199	1.16563
C	5.1684	-3.48232	-2.19568	H	6.011	0.39685	3.94778
N	1.90674	-2.27857	-1.29855	H	8.07464	-1.11956	1.23396
C	2.3066	-2.74991	-0.06194	H	4.7622	2.1875	2.47807
C	3.58356	-3.26133	-0.1171	H	3.04716	-0.03871	-0.62848
C	4.37978	-3.79485	1.04225	H	3.51529	1.41448	0.19889
C	5.04281	-2.72182	1.95242	H	7.10943	3.16786	0.75983
N	4.0657	-1.80475	2.51541	H	6.259	5.24821	2.06897
C	6.05044	-1.7724	1.2316	H	4.65281	4.52995	2.07453
C	4.10448	-0.52148	2.07457	H	4.8425	1.99365	-2.02028
C	5.3764	-0.35488	1.22205	H	3.85233	0.76951	-2.78973
O	3.27715	0.34152	2.3561	H	6.22997	5.49979	-0.36685
C	6.31707	0.75178	1.83133	H	3.75256	3.80349	-0.20439
C	6.84293	0.32981	3.22747	H	2.25515	2.71564	-1.18573
C	7.39177	-1.09426	3.23741	H	8.18485	-2.65378	4.44548
C	7.46309	-1.7637	1.88228	H	7.67347	-1.11498	5.32986
C	5.1276	0.10282	-0.23975	H	7.56052	2.1098	3.51151
C	5.68645	2.12376	1.90573	H	1.7576	2.44504	-3.81793
C	3.8134	0.74775	-0.61743	H	5.68616	6.20394	-2.39165
C	6.19241	3.23422	1.34983	H	5.38478	5.15407	-3.79332
C	5.57971	4.60529	1.48935	H	4.20716	6.37688	-3.35768
C	3.87916	1.47978	-1.95879	H	3.71135	6.6377	0.97656
C	5.27791	5.34435	0.15948	H	4.50904	7.3059	-0.45465
C	4.35749	4.53893	-0.73026	H	5.34623	7.31174	1.11023
C	4.19815	4.65549	-2.06511	H	8.16725	-3.53099	0.83826
C	2.7038	2.47764	-2.16011	H	9.15669	-3.08002	2.23527
C	3.23696	3.77598	-2.79163	H	7.6003	-3.87845	2.47952
C	7.75981	-1.65608	4.39127	H	0.78286	-0.95169	-2.39887
O	7.89704	1.20429	3.63229	H	0.65967	-0.35958	0.12223
O	6.0426	-0.01416	-1.03406	H	-2.44678	-3.04184	-2.69042
O	1.68368	1.94539	-2.97859	H	-1.85992	-1.39247	-2.87776
O	2.90303	4.02876	-3.94587	H	-0.28325	-3.68238	-1.57022
C	4.91685	5.65434	-2.93844	H	-0.02993	-3.07668	-3.19889
C	4.67525	6.73412	0.46338	H	-1.06612	0.21193	1.67001
C	8.12899	-3.14319	1.86267	H	-3.42668	0.47678	2.16168
C	0.58784	-1.7132	-1.62853	H	-3.36232	-0.15509	-1.50198
C	-0.0138	-1.00328	-0.44301	H	-6.8473	-1.50236	-2.30972
C	-1.31755	-1.04473	-0.09785	H	-6.01463	0.01397	-1.98483

Atom	X	Y	Z	Atom	X	Y	Z
C	-2.34786	-1.9129	-0.82927	H	-4.47674	-1.74116	-2.85867
C	-1.80641	-2.28009	-2.23063	H	-4.87748	-2.79309	-1.51511
C	-0.35985	-2.78529	-2.19522	H	-7.42109	0.95547	-0.35561
C	-1.80885	-0.25089	1.02275	H	-7.93781	1.21969	1.98554
C	-3.12646	-0.08163	1.27873	H	-8.16686	-0.50002	2.21818
C	-4.16405	-0.59732	0.39612	H	-5.90153	-0.56738	2.87152
C	-3.66308	-1.07129	-0.96523	H	-5.58048	1.01952	2.1984
C	-5.47527	-0.56717	0.72674	H	-9.51161	-1.18785	0.25773
C	-6.60823	-1.03473	-0.18571	H	-9.85364	1.84796	-0.14538
C	-6.11647	-1.0271	-1.64726	H	-11.35176	-0.28107	1.46424
C	-4.76471	-1.74116	-1.80115	H	-11.61343	2.77562	1.16523
C	-7.72116	0.02324	0.14971	H	-14.03894	2.55053	1.48606
C	-7.56483	0.2396	1.67626	H	-12.97037	1.19837	-1.05134
C	-6.06055	0.05718	1.98245	H	-14.55883	1.96609	-0.87855
C	-9.19418	-0.26472	-0.24694	H	-13.08407	2.94253	-0.77324
C	-10.08834	0.85332	0.24445	H	-15.23746	0.44774	1.03364
C	-11.12679	0.71438	1.07437	H	-14.19544	0.22909	2.44351
C	-12.02325	1.83171	1.55394	H	-13.71956	-0.44069	0.87284
C	-13.4811	1.73252	1.00478	H	-3.29578	-3.8687	-0.49757
C	-13.52591	1.97343	-0.51076	H	-1.66443	-3.74288	0.17024
C	-14.19258	0.41791	1.36425	H	-3.01083	-2.94835	0.98973
C	-2.59638	-3.19321	0.00773	H	-6.23881	-3.17333	0.15007
C	-7.06306	-2.45847	0.2279	H	-7.41664	-2.48766	1.26397
C	-9.41296	-0.44848	-1.76309	H	-7.87851	-2.81443	-0.4132
C	-11.99454	1.91895	3.09273	H	-8.91	-1.34063	-2.14656
H	6.14623	-3.47853	-4.10455	H	-9.03869	0.41594	-2.3257
H	4.26979	-2.39091	-5.29927	H	-10.48194	-0.54913	-1.98006
H	2.19097	-1.76509	-4.10358	H	-12.32876	0.985	3.55974
H	6.00052	-3.95901	-1.68303	H	-12.64582	2.72469	3.45309
H	1.63289	-2.66725	0.77955	H	-10.97822	2.11771	3.45012
H	3.73926	-4.418	1.68272				

Table S7. The coordinates for the lowest-energy conformer of (21*S*,22*S*)-**3** in calculation

Atom	X	Y	Z	Atom	X	Y	Z
C	-9.39257	0.96789	0.12839	H	1.91732	0.95344	-2.43738
C	-9.83064	2.1539	0.75306	H	-10.04691	0.10072	0.11694
C	-9.02776	3.28764	0.79025	H	-10.81459	2.1827	1.21284
C	-7.76895	3.20694	0.1854	H	-9.36682	4.20191	1.27043
C	-7.30374	2.02217	-0.44939	H	-7.81782	-0.02831	-0.949
C	-8.14161	0.89246	-0.4702	H	-6.82248	5.10349	0.39667
N	-6.77151	4.15331	0.06417	H	-4.82923	4.20105	-0.84338
C	-5.70626	3.60587	-0.62534	H	-5.62383	0.85614	-2.505
C	-5.98215	2.30213	-0.96326	H	-4.26357	1.92599	-2.18089
C	-5.07284	1.36067	-1.70185	H	-5.2431	-0.32341	-0.35527
C	-4.4386	0.27872	-0.79871	H	-1.69224	-2.89934	-0.25483
C	-3.39518	-0.6227	-1.51703	H	-4.28871	-1.94908	1.00196
C	-2.20505	-0.78286	-0.52124	H	-2.99443	-2.4603	-2.45968
C	-2.25967	-2.142	0.29883	H	-3.96418	1.70544	0.77083
C	-3.72178	-2.68616	0.40314	H	-5.03491	-2.7879	-3.73721
C	-4.42098	-2.85067	-0.93822	H	-5.83813	-1.43547	-2.93505
C	-3.89963	-1.97367	-2.06179	H	-4.44253	-1.14496	-3.99685
N	-3.64228	0.89302	0.25861	H	-5.96238	-3.85444	-1.99791
C	-2.38157	0.42628	0.41807	H	-5.72242	-4.35224	-0.23305
O	-1.54166	0.87832	1.19016	H	-2.21538	-1.3715	2.37923
H	-3.00365	-0.05859	-2.36812	H	-0.27913	-3.58887	1.54674
C	-4.86226	-1.82449	-3.2472	H	-0.14593	-3.99521	3.93047
C	-5.42431	-3.72318	-1.06451	H	-1.18236	-2.62724	4.32152
C	-1.72201	-2.07944	1.71281	H	1.61185	-2.19606	3.15255
C	-0.79645	-2.90463	2.22134	H	-0.11616	-0.48732	5.03373
C	-0.36314	-2.9519	3.66566	H	0.92584	2.54844	3.82912
C	0.90491	-2.09674	3.97711	H	-0.57968	2.11082	3.02647
C	0.50723	-0.65359	4.15027	H	-0.34403	1.70086	4.73986
C	0.73609	0.43394	3.3834	H	0.91491	-2.53082	6.12685
C	0.15228	1.77707	3.7664	H	2.48201	-2.00216	5.48803
C	1.59016	-2.5966	5.2638	H	1.89621	-3.64367	5.15626
C	1.23415	1.26454	0.94132	H	0.36592	-1.11004	0.42949
C	1.51018	0.30018	2.11591	H	0.29839	1.44778	-0.88607
C	-0.85493	-0.68031	-1.2976	H	0.07046	-3.40047	-1.37105
O	-0.87827	-0.55833	-2.51141	H	0.45771	-5.64359	-1.18628
O	1.2794	2.45822	1.183	H	1.7727	-5.95036	-2.31565
C	0.49858	-0.72036	-0.5782	H	1.93209	-6.27685	0.70857
C	1.46715	-1.67993	-1.37223	H	4.0459	-6.73526	-1.45221
C	2.91799	-1.40271	-1.06488	H	4.23415	-7.09123	0.2641
C	3.32658	-0.13812	-1.19422	H	3.96456	-4.72081	0.8616
C	2.3241	0.96568	-1.41742	H	5.28299	-4.89505	-0.28556
C	1.04905	0.75386	-0.49443	H	5.7613	-2.68863	0.38334

Atom	X	Y	Z	Atom	X	Y	Z
C	1.12291	-3.14022	-1.25482	H	5.75239	-2.19384	-1.2987
C	2.01284	-4.14188	-1.18049	H	6.46065	-0.35379	0.20767
C	3.52624	-3.89269	-1.09461	H	4.9312	-0.45092	1.08384
C	3.76544	-2.48885	-0.45011	H	3.47488	2.6618	-2.13512
C	1.54576	-5.58105	-1.29988	H	2.5647	3.04025	-0.67871
C	2.2284	-6.53942	-0.3153	H	5.64344	-0.48583	-2.67828
C	3.74585	-6.41056	-0.4435	H	5.06118	1.15537	-2.93731
C	4.19362	-4.96546	-0.18586	H	6.54705	0.88389	-2.0128
C	5.24022	-2.05034	-0.34006	H	5.84008	3.07036	-1.32888
C	5.39316	-0.57827	0.09775	H	4.40236	4.17651	1.15645
C	4.71833	0.39506	-0.89614	H	5.47621	5.39733	-1.43825
C	3.13981	2.25602	-1.17196	H	5.32953	7.39764	0.0651
C	4.37315	1.82264	-0.33448	H	4.17038	7.28672	-2.07471
C	5.53985	0.49548	-2.20541	H	1.74038	7.0203	-2.2713
C	5.47584	2.91206	-0.30412	H	2.42815	5.5522	-1.55151
C	4.89669	4.22288	0.18512	H	1.57476	6.72491	-0.537
C	4.97529	5.38716	-0.4661	H	7.39659	3.36137	0.62316
C	4.44984	6.73701	-0.024	H	7.21021	1.65359	0.1949
C	3.5756	7.3717	-1.15203	H	6.36524	2.30853	1.60536
C	2.25659	6.62155	-1.38964	H	4.46002	6.3523	2.11886
C	6.68255	2.53172	0.57901	H	2.89487	6.04379	1.35846
C	3.76573	6.70818	1.34983	H	3.43125	7.70727	1.64722
C	3.32213	8.86926	-0.9206	H	2.81985	9.31536	-1.78739
C	4.10658	-3.96653	-2.52908	H	4.26005	9.41538	-0.76046
O	1.77321	-7.88026	-0.48162	H	2.68021	9.04291	-0.04875
O	-3.73617	-3.94082	1.065	H	3.80862	-4.88845	-3.03953
O	2.42889	-0.49755	1.96253	H	3.75005	-3.12505	-3.13296
H	3.38294	-2.58262	0.5801	H	5.20181	-3.94073	-2.51998
H	4.03833	1.67405	0.70294	H	2.01186	-8.15508	-1.38209
H	1.29229	-1.40829	-2.42452	H	-3.20072	-3.83994	1.87065

Table S8. The coordinates for the lowest-energy conformer of (21*S*,22*S*)-**4** in calculation

Atom	X	Y	Z	Atom	X	Y	Z
C	-9.27091	1.84322	0.44976	H	2.4325	0.48735	-2.54488
C	-9.64185	3.10858	0.94988	H	-9.92541	0.99265	0.61844
C	-8.83746	4.22492	0.75435	H	-10.57479	3.2137	1.49665
C	-7.64546	4.04529	0.04435	H	-9.12571	5.20028	1.13784
C	-7.24822	2.77908	-0.4672	H	-7.81401	0.68951	-0.63733
C	-8.08599	1.66966	-0.25366	H	-6.6816	5.93906	-0.10288
N	-6.66427	4.95149	-0.30384	H	-4.82505	4.8476	-1.40204
C	-5.67549	4.30018	-1.01724	H	-5.79313	1.29755	-2.47523
C	-5.98552	2.96692	-1.14412	H	-4.41337	2.39488	-2.4804
C	-5.15678	1.91747	-1.83005	H	-5.16819	0.45897	-0.24079
C	-4.42017	0.97847	-0.85407	H	-1.73613	-2.20335	0.05533
C	-3.46555	-0.04073	-1.55021	H	-4.5253	-1.3197	0.84001
C	-2.25504	-0.18226	-0.57974	H	-3.73589	2.61046	0.41614
C	-2.3919	-1.40172	0.40879	H	-5.16066	-0.65745	-3.7349
C	-3.82578	-2.03063	0.36381	H	-3.55019	-1.28998	-4.0508
C	-4.28101	-2.31559	-1.0577	H	-4.88753	-2.40313	-3.7223
C	-4.09968	-1.34868	-1.97673	H	-5.26601	-3.77877	-2.3255
N	-3.5002	1.71525	0.00545	H	-4.2083	-4.46978	-1.07832
C	-2.27308	1.16639	0.17053	H	-2.59783	-0.32047	2.34335
O	-1.34184	1.65339	0.80304	H	-0.65705	-2.67329	2.08991
H	-3.07331	0.4598	-2.44069	H	-0.94944	-2.69542	4.54026
C	-4.45073	-1.44311	-3.44132	H	-1.72863	-1.11428	4.53377
C	-4.9142	-3.66189	-1.29905	H	1.18729	-1.47442	3.66682
C	-2.04992	-1.12811	1.85568	H	-0.19339	0.93828	4.95338
C	-1.21842	-1.88101	2.58951	H	-0.02055	2.96612	4.14897
C	-0.93648	-1.70784	4.05777	H	1.38923	3.31979	3.12619
C	0.44998	-1.05236	4.34988	H	-0.17867	3.02887	2.38015
C	0.35487	0.43823	4.14974	H	0.16558	-0.96099	6.52224
C	0.76753	1.24035	3.14325	H	1.86138	-0.88823	6.01255
C	0.47436	2.7253	3.20186	H	0.98496	-2.42869	5.95808
C	0.89156	-1.34864	5.79548	H	0.22943	-0.83006	0.34302
C	1.48177	1.44423	0.61669	H	0.82039	1.50623	-1.33662
C	1.45195	0.66196	1.94895	H	-0.73051	-3.00692	-1.67086
C	-0.92323	-0.24321	-1.38909	H	-1.07062	-5.23704	-1.36482
O	-0.93203	-0.03319	-2.58995	H	0.25008	-6.01452	-2.22853
O	1.78352	2.62432	0.66641	H	-0.19417	-6.10781	0.77506
C	0.40541	-0.54413	-0.69229	H	2.00909	-7.34863	-0.93999
C	1.08825	-1.76584	-1.4255	H	1.80251	-7.59857	0.79292
C	2.5076	-1.92138	-0.94925	H	2.1414	-5.20697	1.26259
C	3.29269	-0.84786	-1.0681	H	3.52901	-5.85605	0.40206
C	2.70427	0.47669	-1.4813	H	4.60227	-3.84561	0.98522
C	1.31572	0.73867	-0.73516	H	4.96519	-3.53744	-0.70175

Atom	X	Y	Z	Atom	X	Y	Z
C	0.31415	-3.0547	-1.36268	H	6.00467	-1.8511	0.79198
C	0.84011	-4.26414	-1.11257	H	4.42054	-1.40494	1.43152
C	2.3234	-4.46882	-0.76895	H	4.40269	1.65127	-2.14052
C	2.89209	-3.14729	-0.15919	H	3.49971	2.45411	-0.86657
C	-0.01196	-5.5076	-1.28695	H	6.74493	-0.92981	-1.4628
C	0.17669	-6.5364	-0.17312	H	5.54332	-2.01406	-2.18044
C	1.6614	-6.86738	-0.01668	H	5.54474	-0.30563	-2.60533
C	2.46918	-5.59996	0.28893	H	6.66163	1.43095	-1.03523
C	4.40251	-3.15482	0.15724	H	5.37102	3.15565	1.16186
C	4.94287	-1.75826	0.535	H	7.0546	3.73769	-1.32479
C	4.73237	-0.72798	-0.59943	H	7.36052	5.84165	-0.01286
C	3.8486	1.47947	-1.20869	H	5.68032	7.30522	-0.99127
C	4.78053	0.78723	-0.17767	H	7.68206	6.55211	-2.30571
C	5.69775	-1.0081	-1.77859	H	6.71446	5.24996	-3.021
C	6.15533	1.49347	-0.06185	H	6.29142	6.93422	-3.33403
C	5.95921	2.95855	0.26424	H	7.34356	-0.17199	0.7504
C	6.46563	3.97761	-0.43726	H	6.59643	0.85107	1.98615
C	6.33397	5.45564	-0.13595	H	8.00638	1.43476	1.0878
C	5.73784	6.26128	-1.33571	H	4.56002	5.37531	1.15519
C	6.66095	6.24556	-2.56483	H	5.54695	6.83222	1.35355
C	7.078	0.85968	1.00023	H	6.0953	5.28866	2.02683
C	5.58843	5.75172	1.17304	H	3.92438	6.44638	-2.52733
C	4.31913	5.81672	-1.72066	H	3.62233	5.88373	-0.87859
C	3.06527	-4.85516	-2.0737	H	4.31336	4.77855	-2.07207
O	-0.60222	-7.67695	-0.53154	H	2.5693	-5.68244	-2.59128
O	-3.83003	-3.2459	1.09822	H	3.09645	-4.00434	-2.76299
O	2.08447	-0.39012	1.96551	H	4.09532	-5.16928	-1.87122
H	2.36469	-3.02977	0.80358	H	-0.49089	-8.3387	0.169
H	4.29914	0.8503	0.80932	H	-3.38405	-3.0587	1.94203
H	1.10238	-1.46366	-2.4842	H	-5.7653	-3.81418	-0.62431

Table S9. The coordinates for the lowest-energy conformer of (21*S*,22*S*)-**5** in calculation

Atom	X	Y	Z	Atom	X	Y	Z
C	0.15444	-4.01825	-3.05942	H	1.18287	-3.83706	-2.75983
C	-0.10889	-4.89373	-4.13281	H	0.71949	-5.37312	-4.64687
C	-1.41032	-5.1564	-4.54352	H	-1.61387	-5.83363	-5.36904
C	-2.44727	-4.51787	-3.85516	H	-0.65635	-2.71576	-1.55195
C	-2.20926	-3.62619	-2.77143	H	-4.28685	-5.16489	-4.70906
C	-0.87813	-3.38544	-2.37867	H	-5.51486	-3.71798	-3.0629
N	-3.81365	-4.58925	-4.03028	H	-4.76828	-2.38381	-0.77153
C	-4.43543	-3.78479	-3.09533	H	-3.05701	-2.33123	-0.37426
C	-3.49843	-3.16821	-2.3018	H	-4.49342	-0.57535	-2.42567
C	-3.77451	-2.19051	-1.1924	H	-3.91041	3.19054	-0.19532
C	-3.73524	-0.7115	-1.64181	H	-3.89717	2.14801	-3.06095
C	-3.96985	0.317	-0.49325	H	-5.37909	1.6672	0.33245
C	-2.81056	1.39155	-0.60527	H	-1.98087	-0.83287	-2.92026
C	-3.37293	2.7887	-1.0585	H	-6.60318	-0.79371	-1.02166
C	-4.40638	2.62571	-2.20387	H	-6.26048	-0.6195	0.71151
C	-5.58467	1.75222	-1.78105	H	-7.44807	0.43064	-0.06827
C	-5.37994	0.95376	-0.50514	H	-7.56542	1.13729	-2.2465
N	-2.43193	-0.33164	-2.16437	H	-6.79613	2.36629	-3.39302
C	-1.8788	0.80817	-1.68031	H	-1.58107	3.48193	-2.18675
O	-0.82099	1.2909	-2.07745	H	-2.99483	5.32745	-0.18877
H	-3.86625	-0.21544	0.45621	H	-1.81079	6.97417	-1.71915
C	-6.48389	-0.06808	-0.2087	H	-0.6359	5.73355	-2.15711
C	-6.70241	1.73986	-2.5116	H	-1.03545	6.82595	0.67705
C	-2.31104	3.792	-1.4421	H	1.32122	5.22557	-0.49898
C	-2.26688	5.037	-0.94956	H	-0.83526	5.94791	2.55169
C	-1.27113	6.09432	-1.33694	H	-1.00371	4.19599	2.7021
C	-0.3737	6.56674	-0.15829	H	0.31828	5.03073	3.53208
C	0.57896	5.4767	0.26321	H	-0.26992	8.63784	-0.85554
C	0.63692	4.81015	1.42557	H	1.07903	7.62738	-1.40405
C	-0.27401	5.01245	2.61105	H	1.02579	8.19052	0.27364
C	0.4106	7.83111	-0.55786	H	2.33956	3.73416	0.7024
C	1.13616	2.36522	1.85607	H	-0.89702	1.20987	2.39549
C	1.73775	3.76603	1.61951	H	0.13345	2.37637	-0.02981
C	-2.18948	1.58282	0.82108	H	-2.87229	-0.75252	2.19644
O	-2.84958	2.2365	1.61249	H	-3.78161	-2.30348	3.58382
O	1.26518	1.88038	2.97333	H	-3.28223	-3.86064	2.93674
C	-0.87478	0.95971	1.33336	H	-2.45014	-2.72417	5.62652
C	-0.84733	-0.61212	1.28996	H	-1.70193	-5.35055	4.25474
C	0.54251	-1.13002	1.59969	H	-1.24941	-4.89594	5.89711
C	1.55986	-0.6075	0.90854	H	0.02239	-2.95075	5.10329
C	1.36222	0.59287	0.01617	H	0.63754	-4.41956	4.36163
C	0.40831	1.64221	0.73156	H	2.38513	-2.93659	3.89017

Atom	X	Y	Z	Atom	X	Y	Z
C	-1.89446	-1.23268	2.18198	H	2.30227	-3.4244	2.20765
C	-1.73945	-2.36327	2.88764	H	4.24009	-1.90534	2.64428
C	-0.38941	-3.08945	2.9762	H	3.13779	-0.65891	3.23635
C	0.74108	-2.02327	2.80433	H	3.10686	0.61875	-1.2632
C	-2.94369	-3.00838	3.54677	H	2.91943	2.132	-0.40855
C	-2.6692	-3.56285	4.94262	H	3.33204	-1.63708	-0.97612
C	-1.45754	-4.49269	4.8953	H	4.5133	-2.27316	0.17075
C	-0.22373	-3.74194	4.38027	H	2.85518	-2.90773	0.14786
C	2.18411	-2.56562	2.87858	H	5.66842	0.00114	1.50895
C	3.23072	-1.48946	2.52371	H	5.39247	0.54774	-1.51663
C	3.02975	-0.94461	1.09526	H	7.27057	-1.17563	0.17582
C	2.8122	1.04949	-0.29984	H	6.97454	-0.65098	-2.8517
C	3.67652	0.46107	0.84401	H	9.46362	-1.32876	-1.22089
C	3.46071	-2.00115	0.04878	H	9.17471	-0.9314	-4.2527
C	5.21749	0.5885	0.69572	H	10.71568	-1.01143	-3.38751
C	5.80876	0.10529	-0.60898	H	9.66174	-2.42715	-3.43631
C	6.83883	-0.7377	-0.72943	H	5.31683	2.69019	0.06602
C	7.46935	-1.18771	-2.02738	H	6.7244	2.15002	0.98592
C	8.98383	-0.82996	-2.07851	H	5.18659	2.4848	1.81705
C	9.6686	-1.33556	-3.35887	H	6.14301	-2.91737	-2.1312
C	5.63527	2.06472	0.90882	H	7.73628	-3.28512	-1.45337
C	7.21229	-2.69749	-2.21873	H	7.54638	-3.05082	-3.19943
C	9.20913	0.68305	-1.93207	H	8.7644	1.22596	-2.77691
C	-0.3341	-4.17697	1.87641	H	10.27985	0.91836	-1.91502
O	-3.85904	-4.23334	5.35669	H	8.75861	1.07423	-1.0151
O	-4.92072	3.88826	-2.60928	H	-1.1963	-4.84986	1.93503
O	2.57615	4.08933	2.70951	H	-0.33504	-3.72825	0.8773
H	0.61251	-1.34678	3.6674	H	0.5671	-4.7935	1.9691
H	3.4406	1.03743	1.748	H	-3.69899	-4.59003	6.24451
H	-1.08208	-0.9059	0.25435	H	-4.15308	4.479	-2.70459
H	0.83914	0.35819	-0.91453	H	2.38931	3.39876	3.37839

Table S10. The cytotoxic activities of compounds **1–12** against human non-small cell lung cancer A549 cells

Compound	Cytotoxicity (IC_{50} in μmol/L)
**1**	>50
**2**	>50
**3**	>50
**4**	>50
**5**	>50
**6**	>50
**7**	>50
**8**	>50
**9**	9.26
**10**	9.14
**11**	14.89
**12**	5.14
cisplatin	2.39
